# Hydrotalcite-Derived Copper-Based Oxygen Carrier Materials for Efficient Chemical-Looping Combustion of Solid Fuels with CO_2_ Capture

**DOI:** 10.1021/acs.energyfuels.2c02409

**Published:** 2022-08-26

**Authors:** Michael High, Clemens F. Patzschke, Liya Zheng, Dewang Zeng, Rui Xiao, Paul S. Fennell, Qilei Song

**Affiliations:** † Department of Chemical Engineering, Imperial College London, LondonSW7 2AZ, United Kingdom; ‡ School of Materials, Sun Yat-sen University, Guangzhou510275, China; § MOE Key Laboratory of Energy Thermal Conversion and Control, School of Energy and Environment, Southeast University, Nanjing210096, China

## Abstract

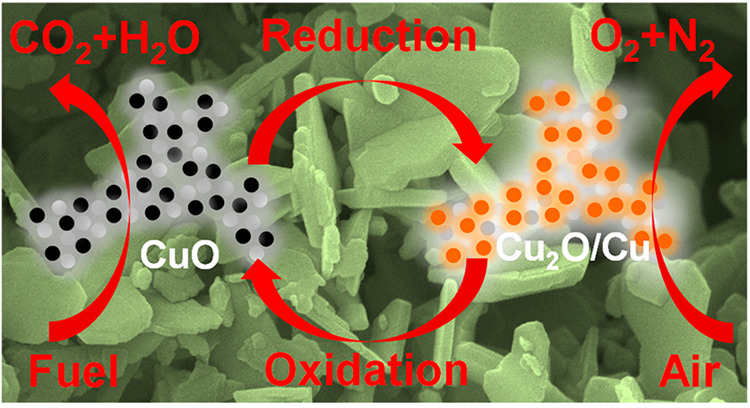

Chemical-looping combustion (CLC) is a promising technology that utilizes metal oxides as oxygen carriers for the combustion of fossil fuels to CO_2_ and H_2_O, with CO_2_ readily sequestrated after the condensation of steam. Thermally stable and reactive metal oxides are desirable as oxygen carrier materials for the CLC processes. Here, we report the performance of Cu-based mixed oxides derived from hydrotalcite (also known as layered double hydroxides) precursors as oxygen carriers for the combustion of solid fuels. Two types of CLC processes were demonstrated, including chemical looping oxygen uncoupling (CLOU) and *in situ* gasification (iG-CLC) in the presence of steam. The Cu-based oxygen carriers showed high performance for the combustion of two solid fuels (a lignite and a bituminous coal), maintaining high thermal stability, fast reaction kinetics, and reversible oxygen release and storage over multiple redox cycles. Slight deactivation and sintering of the oxygen carrier occurred after redox cycles at an very high operation temperature of 985 °C. We expect that our material design strategy will inspire the development of better oxygen carrier materials for a variety of chemical looping processes for the clean conversion of fossil fuels with efficient CO_2_ capture.

## 1 Introduction

Climate change has become one of the defining global challenges in the 21st century.^1^ CO_2_ emitted from the combustion of fossil fuels is considered as a major source of greenhouse gas emissions driving climate change. Over the next decades, the combustion of fossil fuels (oil, coal, natural gas, shale gas, etc.) is likely to still dominate the generation of electricity and conversion of energy in the world. While the global energy industry is transitioning from fossil fuels to more sustainable and renewable energy sources, an immediate solution to continue using fossil fuels while reducing CO_2_ emissions is carbon capture, utilization, and storage (CCUS). Carbon capture involves the separation of CO_2_ from flue gases emitted from combustion processes, e.g., coal-fired power plants. Current commercially available CO_2_ separation technologies, such as the amine solvent scrubbing process, are energy-intensive.^2^

**Figure 1:**
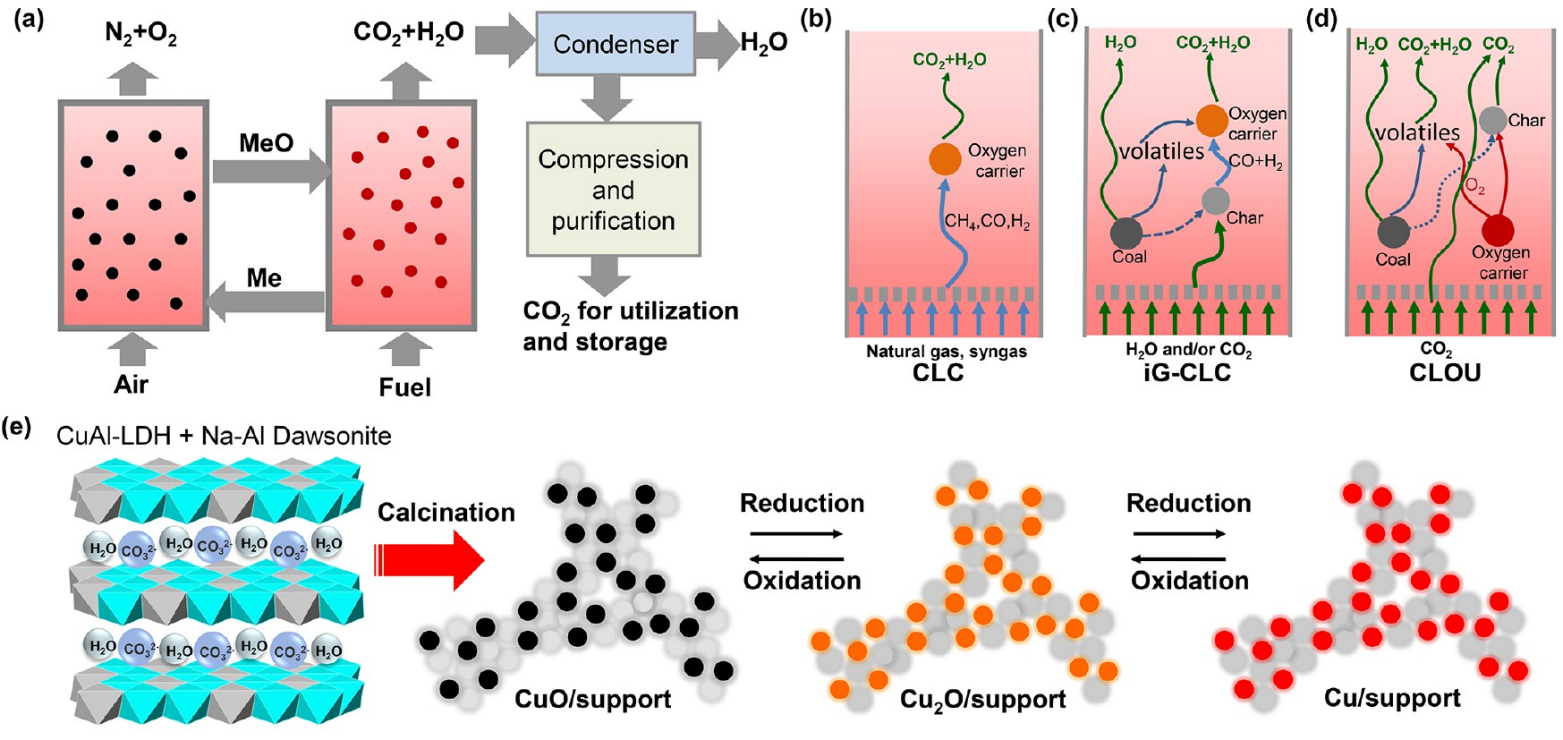
Chemical-looping combustion (CLC) of solid fuels using nanostructured Cu-based oxygen carrier materials. (a) Schematic diagram of the CLC process with inherent CO_2_ capture. The system requires oxygen storage materials (metal oxides, represented as MeO in oxidized state, and Me in reduced state) to be circulated between the two reactors. (b–d) Diagram showing the combustion of (b) natural gas or syngas, (c) solid fuels by the gaseous O_2_ released from metal oxides—the process is known as chemical looping oxygen uncoupling (CLOU)—and (d) solid fuels *via in situ* gasification and combined chemical looping combustion (iG-CLC), adapted from the literature.^15,16^ (e) Schematic diagram showing the calcination of LDH-based precursors (mixed with Na–Al Dawsonite) to generate copper-based oxygen carriers with a high degree of dispersion in the support. The calcined materials exhibit reversible phase change between CuO-Cu_2_O and Cu in chemical looping redox cycles.

An emergent CO_2_ capture technology is chemical-looping combustion (CLC), which shows great potential for efficient utilization of fuels (e.g., coal, natural gas and biomass) while offering a route for cost-effective sequestration of CO_2_.^3−11^ The CLC processes use oxygen carriers, typically metal oxide materials, to provide the oxygen required for oxidation of the fuel to CO_2_ and H_2_O. The reduced oxygen carriers are then typically regenerated by oxidation in air. The basic concept of CLC is illustrated in Figure 1a. It involves two interconnected fluidized bed reactors. One reactor, the fuel reactor, contains a metal oxide, MeO (the oxygen carriers), which oxidizes the fuel to mainly CO_2_ and steam (Figure 1b), yielding almost pure CO_2_ when the steam is condensed. The reduced oxygen carriers, Me, are then transferred to the air reactor, where it is reoxidized. The oxidized metal oxides are recycled to the first reactor to begin a new redox-cycle and thus act as an oxygen storage material. The off-gas from the air reactor is oxygen deficient air. Overall, the fuel is combusted in a CLC process but the resulting CO_2_ is produced separately from the oxygen deficient air, while the total heat released is equivalent to the combustion of the fuel in air. The heat released in the air reactor can be used to raise steam for a steam cycle. When the CLC process is operated at an elevated pressure, the O_2_ depleted air leaving the air reactor at high temperatures (around 1000 °C) can additionally be used to drive a gas turbine for electricity production. The CLC process implicitly reduces the energy penalties of separating CO_2_ and other products associated with a traditional air-based combustion. This technology for the combustion of gaseous fuels, particularly natural gas, has been an active research area for the last two decades.^7,12,13^ These investigations have shown that CLC provides an efficient and cost-effective method for the combustion of gaseous fuels with inherent capture of CO_2_.^14^

Given the vast abundance of solid fuels (coal, petroleum coke, biomass, etc.) and their importance in the generation of electrical power, it is highly attractive to apply CLC to the combustion of solid fuels for CO_2_ capture.^4,13^ One of the processes involves the *in situ* gasification and combustion (iG-CLC) of solid fuels in a fuel reactor where the gasification intermediates are oxidized by the metal oxides to CO_2_ and H_2_O, with the gasification being the rate-limiting step (Figure 1c).^4−6,17^*In situ* gasification CLC has been demonstrated in circulating fluidized bed reactors.^18−20^ A new concept is chemical-looping with oxygen uncoupling (CLOU) (Figure 1d).^21,22^ In the CLOU process, suitable oxygen carrier materials spontaneously releases gaseous O_2_ that is used to burn the solid fuel *in situ*. Thus, for a Cu-based oxygen carrier, the first reaction involves the release of O_2_ into an inert fluidizing gas (containing H_2_O and CO_2_):
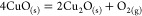
1with the products of pyrolysis and char being burned by the gaseous oxygen:
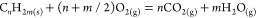
2

The reduced oxygen carriers are then reoxidized:
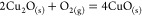
3

The CLOU process allows for faster rates of conversion of coal and biomass compared to the conventional *in situ* gasification CLC process.^21−25^ The CLOU combustion of coal has also been demonstrated in continuous units, showing extraordinary high carbon capture efficiencies (∼100%).^26−28^ While the chemical looping technology has been scaled-up from laboratory prototype to pilot-scale plants worldwide over the past decade, there is an increasing demand for high-performance oxygen storage materials for large-scale implementation of this technology.

One key scientific challenge in CLC and other redox cycles-based processes is the design and fabrication of reactive and stable oxygen carriers. The irreversible structural changes of metal oxides over a large number of repeated redox cycles are especially detrimental to unsupported nanoparticles under reactive environments.^29−33^ In addition, the ideal oxygen carrier materials for the CLOU process should reversibly release or take-up gaseous O_2_ at high temperatures (800–1000 °C) while being resistant to sintering. Good candidates are the transition metal oxides CuO-Cu_2_O, Mn_2_O_3-_Mn_3_O_4_, and Co_3_O_4-_CoO,^21,22^ perovskites,^34^ and spinel oxides.^35,36^ The advantages of the CuO–Cu_2_O redox couple are its relatively low cost, high O_2_ release capacity (i.e., amount of gaseous O_2_ released per unit mass of the metal oxide), and fast rates of reaction in comparison to other materials, e.g., perovskites or spinel oxides. However, Cu-based oxygen carriers usually suffer from thermal sintering and agglomeration due to their low Tammann temperatures (CuO, 526 °C; Cu_2_O, 481 °C; Cu, 405 °C). Sintering can be limited by the use of an inert support, on which the active metal oxides is uniformly dispersed. So far, various Cu-based oxygen carrier materials have been investigated for CLOU, such as CuO supported on Al_2_O_3_, SiO_2_, MgAl_2_O_4_, or ZrO_2_.^21,22,26,37−40^ However, conventional preparation methods (e.g., mechanical mixing, spray drying, etc.) result in a limited dispersion of the active component on the support at high loadings (of the active phase), leading to a compromised performance during long-term redox-cycling. Natural minerals such as copper and manganese ores are also promising low-cost alternatives; however, their reactivity and oxygen release rate are usually relatively low.^41−44^ In contrast, contemporary nanoscience has allowed for the rational design of nanoparticles with well-controlled size, composition, dispersion, active sites, microstructure, and metal–support interaction.^45−48^ Recent scientific understandings of the synthetic chemistry of Cu-based catalysts (Cu/ZnO/Al_2_O_3_)^46,47,49−52^ have important implications for the rational design of nanostructured metal oxides.

In our previous work from 2013,^53^ we reported a novel approach to design oxygen storage materials from layered double hydroxides (LDHs) precursors. LDHs, also known as hydrotalcite-like compounds (HTLCs), are a class of two-dimensional nanostructured anionic clays.^49,54−56^ LDH consists of brucite-like host layers formed of divalent and trivalent metal cations mixed at a molecular level, [M^2+^_1–*x*_M^3+^_*x*_(OH)_2_]^*x*+^ and charge-balancing anions (A^*n*–1^), as illustrated in Figure 1e. Calcination of LDH precursors produces then mixed metal oxides with a high degree of dispersion of the active phase.^54−59^ Our previous work carefully studied the material chemistry for preparing Cu–Al LDH precursors by coprecipitation and the structure of the derived mixed metal oxides. As depicted in Figure 1e, calcination of the Cu–Al LDH precursor in the presence of Na–Al Dawsonite generates mixed metal oxides with CuO nanoparticles well-dispersed in an amorphous Na–Al–O support. Owing to the dispersion of elements at a molecular level in the precursor and the promotion of sodium, the active copper phase in the calcined product stays highly dispersed and shows a strong resistance to sintering. Previous work has studied the composition and loading of CuO in the oxygen carriers. The results suggested that the oxygen carriers with a CuO loading of 60 wt % provided high oxygen capacity and stability during redox-cycling, as examined by oxygen release and uptake cycles (CuO-Cu_2_O) and reduction–oxidation cycles (CuO-Cu) at high temperatures but in the absence of solid fuels.^53^ It is highly desirable to use these synthetic mixed metal oxides for the combustion of solid fuels *via* the promising CLOU approach.

In this study, we report the use of LDH-derived mixed metal oxides as oxygen storage materials for the combustion of coal at high temperatures (800–985 °C) *via* both, the CLOU and iG-CLC, processes. The stability of the materials in combustion of solid fuels at high temperatures and cycling between CuO and Cu was investigated aiming to fully utilize the oxygen storage capacity and achieve a high combustion efficiency. The oxygen carriers were exposed to different types of coal, with and without the addition of steam as a gasification agent as well as to a gaseous fuel (CO/N_2_) over the temperature range 850–985 °C. The fresh and spent oxygen carriers were characterized with various physicochemical techniques to understand the sintering mechanism. We investigated key performance parameters, such as the oxygen release and storage capacity, the rate of coal combustion and CO_2_ production, the interaction of metal oxides with coal ash, and the microstructure and phase changes over multiple redox-cycles. Our results may inspire the development of high-performance oxygen carriers for CLC processes and other applications.

## 2 Results and Discussion

### 2.1 Synthesis and Characterization of Materials

**Figure 2:**
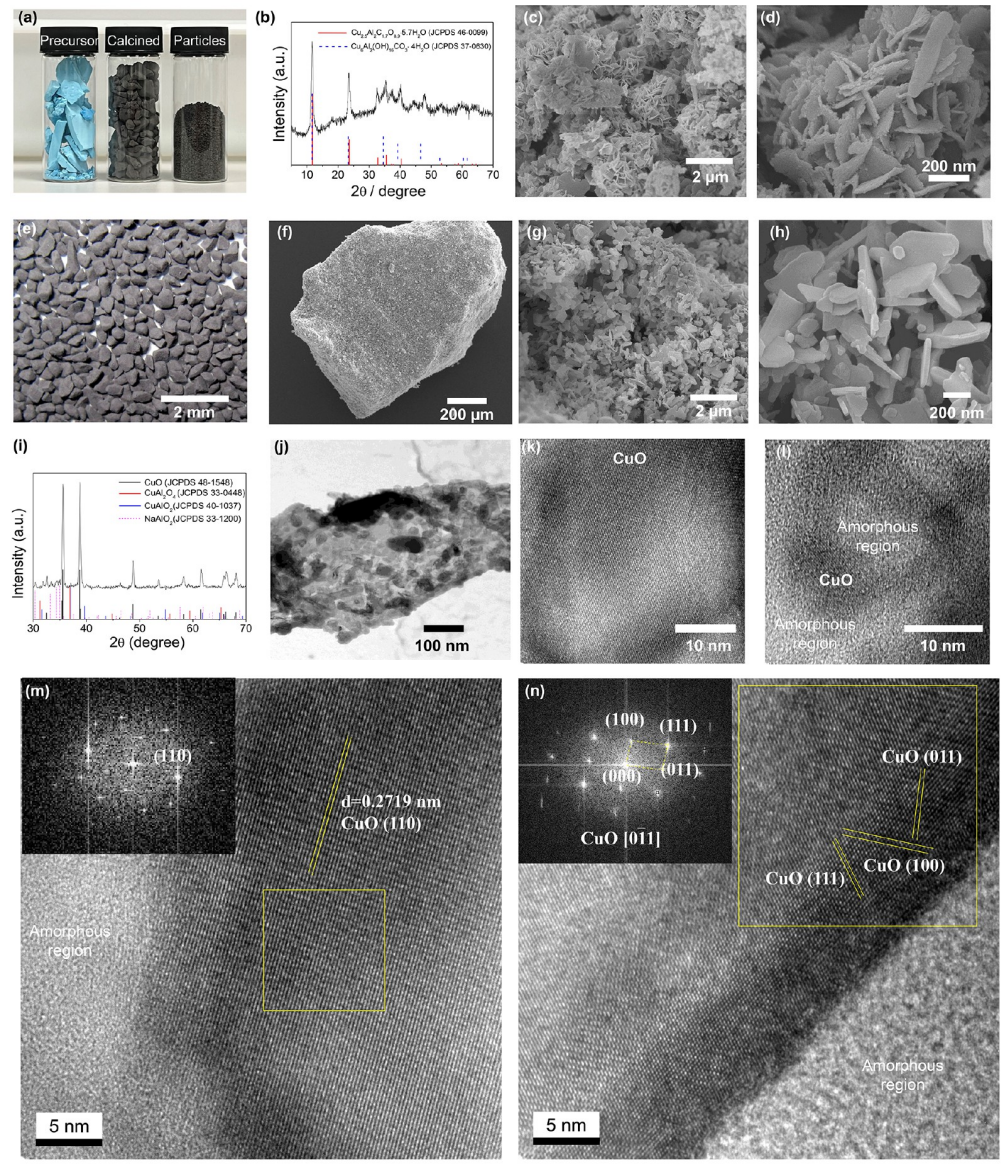
Synthesis and characterization of oxygen carrier materials. (a) Photo of the Cu–Al hydrotalcite precursors, calcined MMOs, and particles. (b) XRD pattern of the precursor. Cu–Al hydrotalcite (JCPDS 46-0099), solid vertical lines and Cu_6_Al_2_(OH)_16_CO_3_·4H_2_O (JCPDS 37-0630), dashed vertical lines. (c and d) SEM images of the precursor surface at low and high magnifications. (e) Photo of a batch of oxygen carrier particles crushed from calcined powders suitable for operation in fluidized bed reactors. (f) SEM image of a typical oxygen carrier particle. (g and h) SEM image of the calcined mixed oxides at low and high magnifications. (i) XRD pattern of the calcined mixed oxides. (j) STEM image of mixed metal oxides calcined from the precursor at 950 °C. (k–n) HRTEM images of mixed metal oxides after calcination. Inset images in (m) and (n) show the fast Fourier transform (FFT) patterns of crystalline regions (yellow squares).

The Cu–Al layered double hydroxides (or hydrotalcite) precursors and calcined mixed metal oxides were synthesized following the synthesis route reported previously.^53^ A detailed synthetic procedure is given in the Supporting Information Methods section. In this study, we characterized the precursors and calcined mixed metal oxides (Figure 2a) using various techniques to understand their structures and properties. X-ray diffraction (XRD) patterns (Figure 2b) of the precursors confirmed the formation of a Cu–Al LDH phase, and the low crystallinity suggests the presence of amorphous phases. The scanning electron microscopy (SEM) images of the precursors (Figure 2c,d) showed the morphology of disordered aggregation of nanosheets with thicknesses of ca. 10–20 nm and a lateral size of ca. 500 nm. Fourier-transform infrared spectroscopy (FTIR) spectra confirmed the intercalation of carbonate anions (Figure S3). Thermogravimetric analysis (Figure S4) confirmed the decomposition of the LDH structures, with the weight loss corresponding to the removal of interlayer water, anions, and decomposition of metal hydroxides.

The precursor powders were calcined at a temperature of 950 °C in air, and crushed and sieved into micrometer-sized particles (Figure 2e,f and Figure S5) to obtain a suitable particle size range for their use in fluidized bed reactors. SEM images of the particles at low and high magnifications (Figure 2g,h) confirmed that the porous structures originated from the LDH precursors, with aggregated grains and nanosheets (30–50 nm) (Figure 2g). The XRD pattern of the calcined mixed metal oxides (Figure 2i) confirmed that crystalline CuO (JCPDS 48-1548) was the dominant phase with very weak peaks corresponding to CuAl_2_O_4_, CuAlO_2_, and NaAlO_2_. The average crystallite size of CuO derived from XRD analysis was about 36 nm. Scanning transmission electron microscopy (STEM) imaging showed that nanoscale CuO nanoparticles were dispersed in an amorphous matrix (Figure 2j). HRTEM images indicate that the size distribution of CuO crystals was not uniform, ranging from about 30–40 nm (Figure 2k) to about 5 nm (Figure 2l). Parts m and n of Figure 2 present two typical HRTEM images and fast Fourier transform (FFT) patterns (Figure 2m,n). Detailed analyses of the lattice spacings are provided in the Supporting Information (Figure S6). The enlargement of lattice fringes in Figure 2m shows a separation of about 2.7 Å, in agreement with the (110) interplanar *d* spacing (0.2719 nm) of monoclinic CuO. Figure 2n shows the HRTEM together with the corresponding FFT image with the beam paralleling to [011] zone axis, and (110), (100), and (111) planes of CuO could be identified.

Inductively coupled plasma atomic emission spectroscopy (ICP-AES) analysis confirmed that the mass fraction of crystalline CuO (soluble in nitric acid) was ∼50.0 wt %. The total content of CuAl_2_O_4_ and CuAlO_2_ accounted for up to 10 wt % based on a mass balance of copper. The sodium content was found to be ∼8.9 wt %, corresponding to a nominal loading of Na_2_O of 12.1 wt %. However, only very weak peaks of NaAlO_2_ (JCPDS 33-1200) were observed in the XRD pattern (Figure 2i). Sodium-containing species (i.e., NaAlO_2_) were probably the calcined product of dawsonite (NaAlCO_3_(OH)_2_, JCPDS 45-1359), which was a contaminant in the LDH precursor that occurred when Na_2_CO_3_ was used as the precipitation agent. The NaAlO_2_ might possibly further interacted with the aluminum phase, forming complex species (Na_*x*_Al_*y*_O_*z*_). Here, these sodium-containing species inhibited the formation of copper aluminates (CuAl_2_O_4_ and CuAlO_2_) at the interface of copper and the surrounding phases. This helped to maintain the aluminum phase in an amorphous state. Thermodynamic analysis have shown that CuO reacts with Al_2_O_3_ to form copper aluminates, which are not as effective as CuO in releasing gaseous oxygen.^40,60,61^ Our materials design strategy generates a highly stable Cu-based mixed oxides owing to the formation of sodium–aluminum support, which limits the interactions between copper and alumina.

### 2.2 Reversible O_2_ Release and Storage

**Figure 3:**
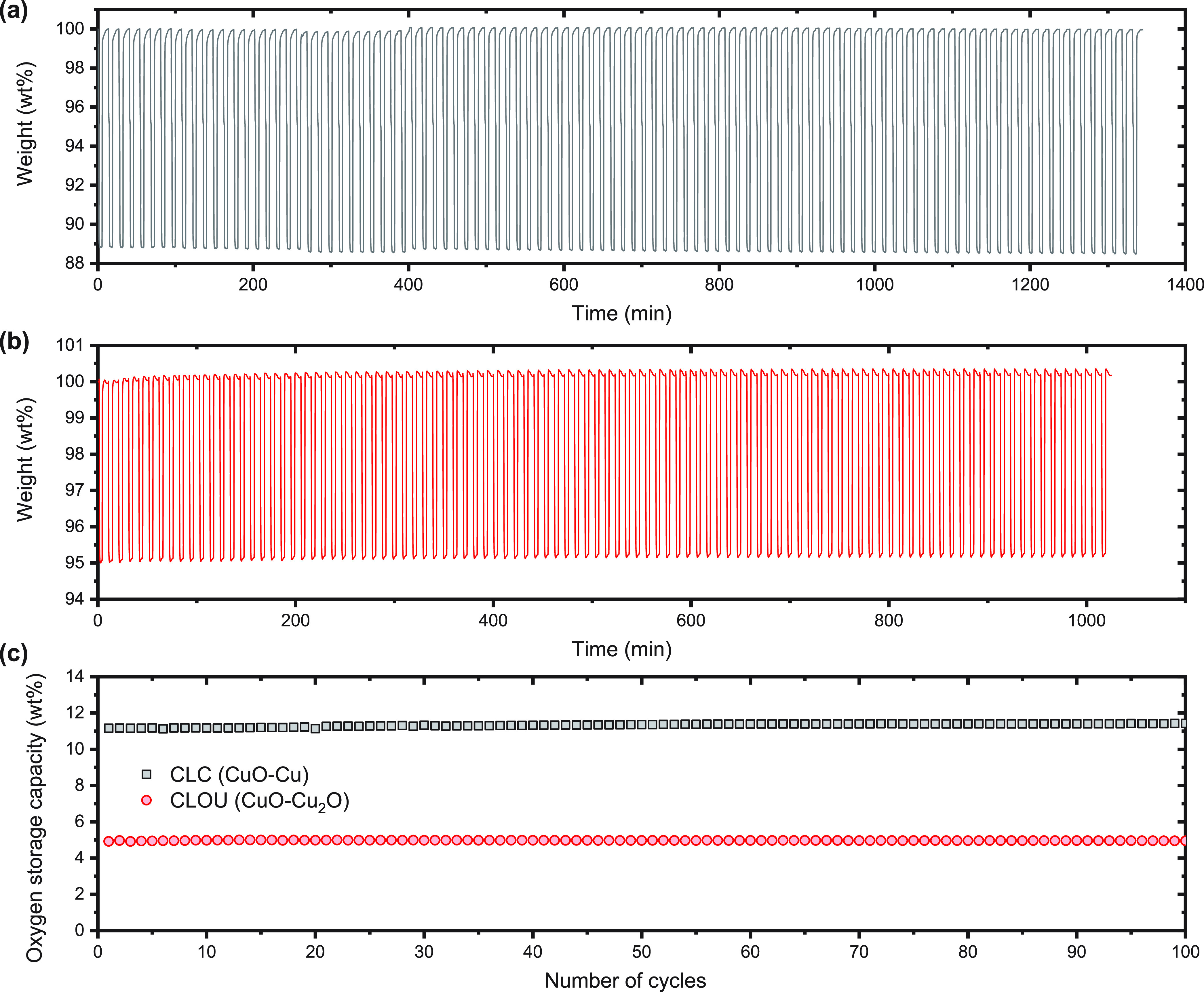
Thermochemical redox cycles of oxygen carrier materials in a TGA. (a) CLC cycling with reducing gas of 10 vol % CO/N_2_ for 3 min, followed by N_2_ purging for 1 min, and air oxidation for 8 min, and 1 min purging with N_2_. (b) CLOU cycling between decomposition in N_2_ for 4 min and oxidation in air for 6 min. (c) Oxygen storage capacity as a function of cycle number for both CLC and CLOU.

To demonstrate the oxygen storage capacity, we performed thermochemical looping redox cycles of the Cu-based oxygen carrier in both CLC and CLOU modes in a thermogravimetric analyzer (TGA). The weight change profiles are presented in Figure 3. In CLC mode, the oxygen carriers were exposed to reduction by 10 vol % CO in N_2_ and then oxidized by air at 900 °C. As presented in Figure 3a, the oxygen carriers exhibited reversible oxygen release and storage during CLC redox cycles. The total oxygen storage capacity was stable at 11 wt % for 100 cycles over a testing period of 1300 min. Similarly, the oxygen carriers showed a stable gaseous O_2_ release capacity of 5 wt % in 100 CLOU redox cycles (phase change between CuO-Cu_2_O) over the testing period of 1000 min, as shown in Figure 3b,c. Compared to pure CuO or supported CuO oxygen carriers reported in the literature, our Cu-based oxygen carriers showed superior thermal stability and strong resistance to sintering owing to the nanoscale dispersion of active Cu phases in the sodium-stabilized support.

**Figure 4:**
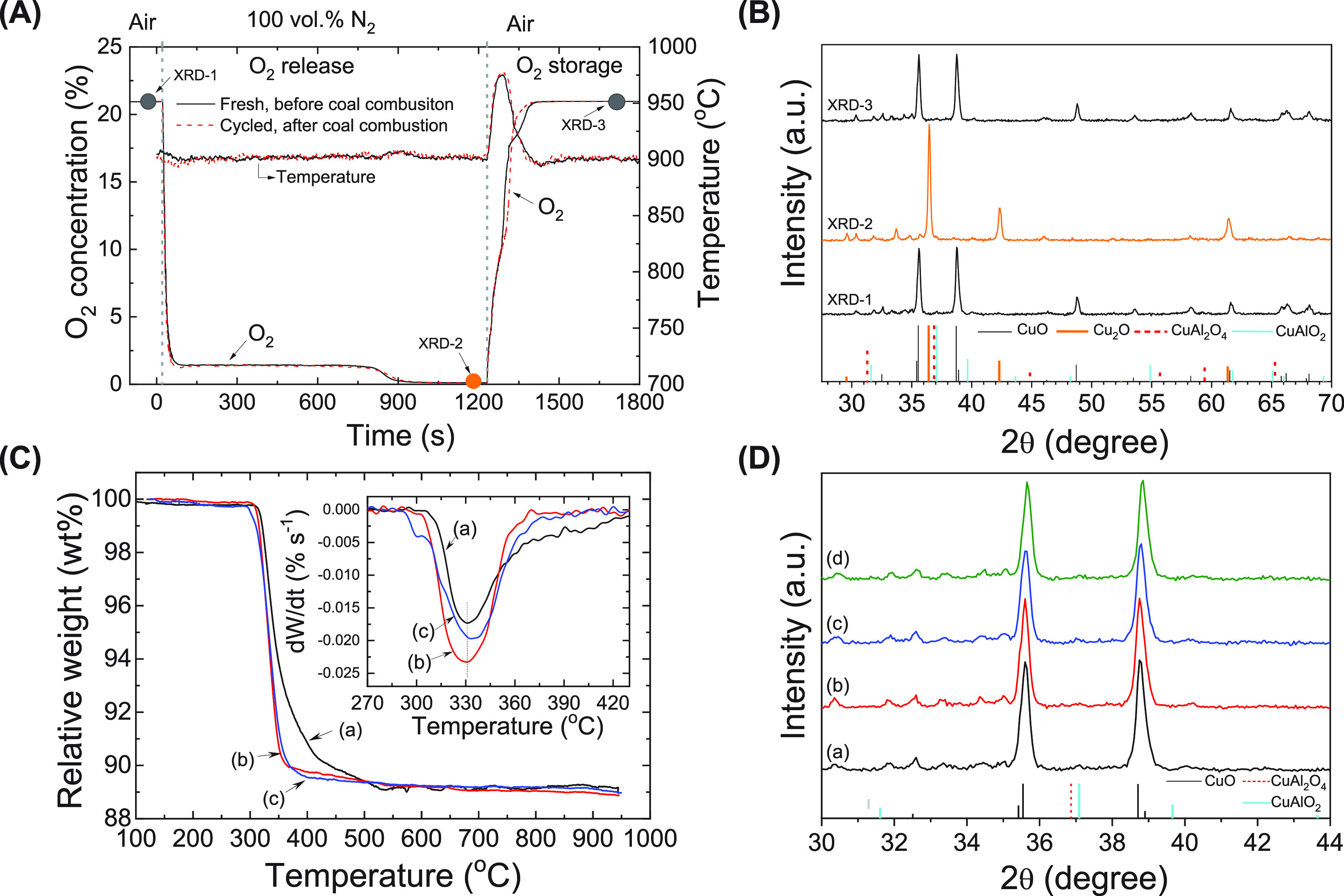
Reversible oxygen release and storage. (A) O_2_ release and storage profiles of fresh and redox-cycled oxygen carrier materials after 20 cycles of combustion of bituminous coal in a fluidized bed reactor. The solid circles indicate the sampling of materials for XRD analysis. (B) Sequential *ex situ* XRD analyses of oxygen carrier particles in its pristine state (XRD-1), after complete O_2_ release (XRD-2), and complete oxidized state after 20 cycles of bituminous coal combustion (XRD-3). The crystalline patterns are CuO (JCPDS 48-1548) and Cu_2_O (JCPDS 05-0667). (C) Temperature-programmed reduction (TPR) profiles: (a) fresh sample and redox-cycled samples in the CLOU experiments after (b) 20 cycles of lignite combustion at 900 °C and (c) 20 cycles of bituminous coal combustion at 900 °C. (D) XRD patterns of fresh and cycled CuO-based mixed metal oxides (oxidized state): (a) as calcined, (b) after 20 cycles with lignite at 900 °C, (c) after 20 cycles with bituminous coal at 900 °C, and (d) after 20 cycles of oxygen release and storage at 900 °C in the absence of solid fuels. The main phase is crystalline CuO (JCPDS 48-1548), with very weak peaks corresponding to CuAl_2_O_4_ (JCPDS 33-0448) and CuAlO_2_ (JCPDS 40-1037).

We further demonstrated that the Cu-based oxygen carriers can reversibly release and take-up O_2_ in the temperature range 800–1000 °C during operation in a laboratory fluidized bed. Fully oxidized oxygen carriers would start to release O_2_ immediately when the O_2_ partial pressure fell below its equilibrium value. Figure 4A shows that the O_2_ concentration, during a stable period of oxygen release (i.e., between 100 and 800 s), was kept at a stable level (between 1.41 and 1.55 vol %), which was close to the equilibrium O_2_ partial pressure (∼1.5 vol %) at the operating temperature (900 °C). This observation indicated that the kinetics of the O_2_ release were limited by the thermodynamic equilibrium. Integration of the amount of O_2_ released over time yields an apparent O_2_ release capacity of 0.05 g O_2_/(g oxygen carrier). Reversible crystalline phase changes (CuO ↔ Cu_2_O) were confirmed by *ex situ* XRD analyses (Figure 4B). The freshly calcined metal oxides contained CuO as the dominant crystalline phase (Figure 3b: XRD 1). After complete O_2_ release, the active phase had transformed to Cu_2_O (Figure 4B: XRD 2), which was readily regenerated back to CuO after a further oxidation step (Figure 4B: XRD 3).

The temperature-programmed reduction of oxygen carriers (Figure 4C) was performed in a TGA with a ∼5 mg sample heated from 120 to 950 °C at rate of 5 °C min^–1^ in 5 vol % H_2_ balanced with N_2_. The fast weight loss (∼10 wt %) in the range 300–400 °C corresponded to the reduction of bulk crystalline CuO (about 50 wt %), and the slow reduction at 400–550 °C was likely caused by the reduction of small amounts of CuAl_2_O_4_ and CuAlO_2_ (weight percentage of ∼10 wt %). The oxygen carriers exposed to multiple redox cycles showed a similar weight loss, confirming their stable oxygen storage capacity. We also examined the XRD patterns of cycled oxygen carriers after CLOU cycles without and with solid fuels (Figure 4D). The formation of thermodynamically stable side products (CuAl_2_O_4_ and CuAlO_2_) was not observed in all experiments. The contents of sodium (∼8.8 wt %) as measured by ICP and other trace elements remained stable over O_2_ release and storage cycles (Table S2). These results confirmed that the oxygen carriers were stable in most of the operation conditions, so we can evaluate the combustion of solid fuels and the durability of oxygen carrier in these operation conditions.

### 2.3 Chemical-Looping Combustion of Solid Fuels

**Figure 5:**
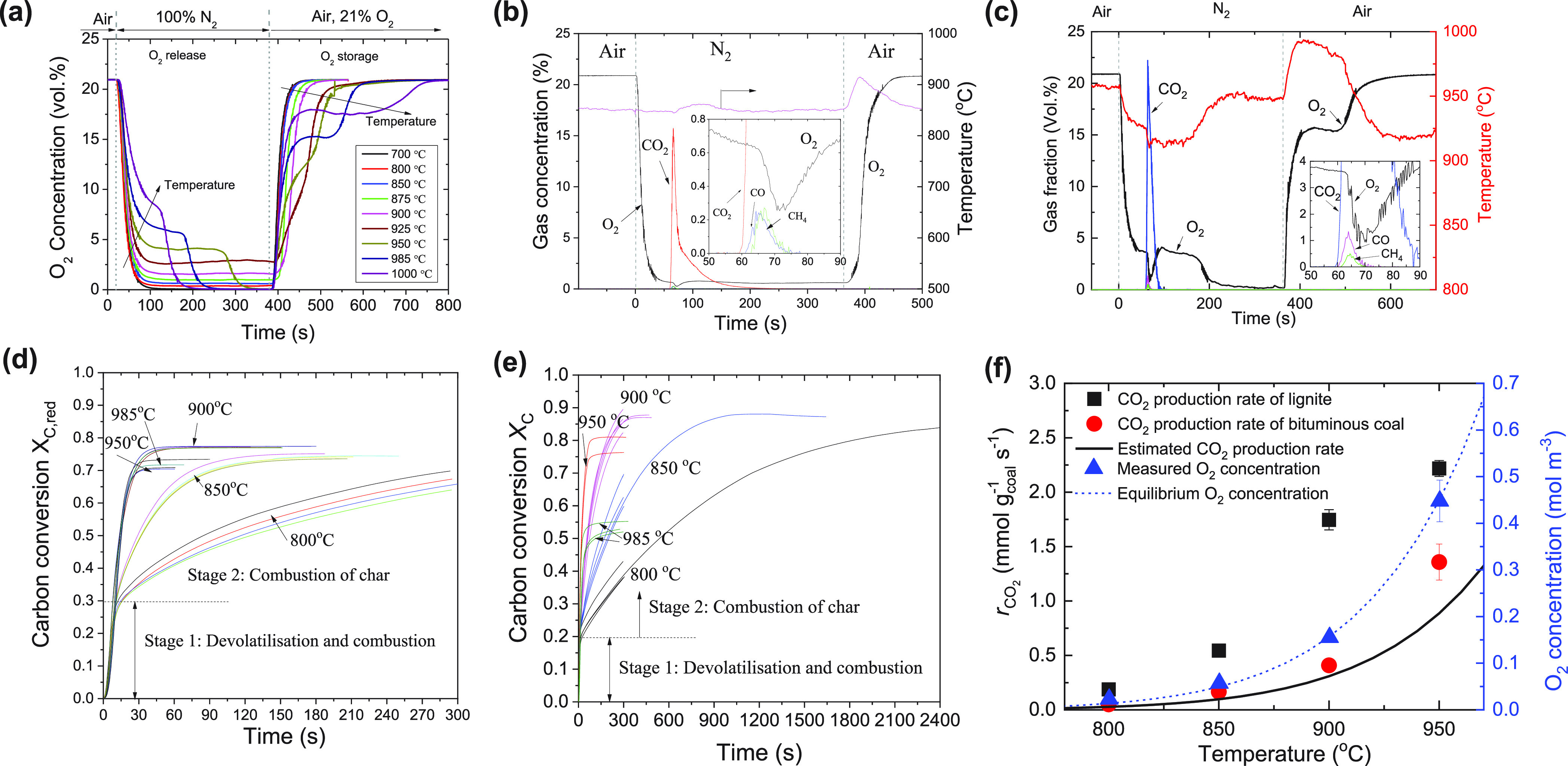
Combustion of solid fuels *via* CLOU process. (a) O_2_ concentration profiles during cyclic reduction and oxidation. (b) Profiles of the combustion of lignite at a set-point temperature of 850 °C. The vertical dashed lines indicate the switch of the fluidizing gases. The inset in (b) shows the detailed profile of gas concentrations during the initial stage of the combustion (i.e., the combustion of volatiles). (c) Combustion of lignite at 950 °C. (d) Carbon conversion as a function of time for the combustion of lignite at 800–985 °C. (e) Carbon conversion as a function of time for the combustion of bituminous coal at 800–985 °C. (f) Rate of CO_2_ production and O_2_ concentration as a function of set-point temperature during the combustion of lignite and bituminous coal in the presence of oxygen carriers in a fluidized bed reactor. The CO_2_ production rates were derived from the second stage of coal combustion (maximum rate of combustion of char). The estimated CO_2_ production rate was calculated with the equilibrium O_2_ partial pressure and the inlet gas flow rate. The equilibrium O_2_ concentration was calculated from the theoretical equilibrium of the CuO/Cu_2_O redox couple.

The combustion is strongly dependent on the concentration of O_2_ released from the oxygen carrier. Figure 5a shows the concentration of O_2_ released from the oxygen carrier, measured in the fluidized bed reactor. The oxygen concentration was close to that of the equilibrium of CuO/Cu_2_O at corresponding temperatures (Figure S7). Two types of coal were tested in batch mode, including a lignite and a bituminous coal (composition analysis shown in Table S1). The N_2_ adsorption isotherms at 77 K for lignite, lignite char, and bituminous coal are presented in Figure S8. Further concentration profiles of the combustion reactions are shown in Figures S9 and S10. A typical profile in Figure 5b shows the measured outlet gas concentration and bed temperature during the combustion of lignite with O_2_ released from the oxygen carriers. A batch of 15 g of Cu-based oxygen carrier was fluidized alternatingly in inert gas (N_2_, 50.0 mL s^–1^, SATP) and oxidizing gas (air, 47.4 mL s^–1^, SATP). At each cycle of oxygen release, a batch of coal (0.2 g) was added to the reactor 60 s after the gas was switched to N_2_, when the partial pressure of O_2_, generated by the oxygen carriers, had reached a stable value in the reactor. In general, when lignite was added to the reactor, rapid combustion of volatiles occurred, generating an initial sharp rise in the volume fraction of CO_2_ in the exhaust gas (e.g., a peak of ∼22 vol % CO_2_ at 950 °C in Figure 5c). Only very low concentrations of CO, CH_4_ and residual O_2_ were detected (e.g., < 1 vol % at 950 °C), irrespective of the operating temperature. After combustion, when the bed of materials was recovered, the oxygen carrier particles were relatively clean without an observable presence of ash from the lignite. This was owing to the low content of ash in this coal and elutriation of the ash from the reactor. In contrast, the combustion of bituminous coal was slower (Figure S10), owing to a lower reactivity of bituminous coal char, as found by Dennis et al.^6^ For both lignite and bituminous coal, the transient N_2_-free concentration of CO_2_ was as high as 95 vol %, which is arguably in a range suitable for sequestration.

Parts d and e of Figure 5 show the carbon conversion for both lignite and bituminous coal in the temperature range 800–985 °C. Typically, the combustion profiles showed rapid combustion of volatiles and slow combustion of char particles. The maximum rates of CO_2_ production during the transition from combustion of volatiles to combustion of char (Figure S11) were assumed as the maximum rate of combustion of char. The rates of CO_2_ production from char combustion against temperature are shown in Figure 5f. The apparent rates of the combustion of solid fuels increased exponentially with temperature, partly because of the increase in the equilibrium O_2_ partial pressure. The apparent activation energy of char combustion was calculated as 233.6 ± 12.2 and 235.4 ± 11.3 kJ mol^–1^ for lignite and bituminous coal, respectively (Figure S12). The measured rate of combustion of bituminous coal char at 900 °C was 4.1 × 10^–4^ mol g_coal_^–1^ s^–1^, comparable to the calculated maximum rate of external mass transfer (3.0 × 10^–4^ mol g_coal_^–1^ s^–1^, see the Supporting Information), indicating very fast reaction kinetics. Interestingly, the measured rate of lignite combustion (e.g., 1.75 × 10^–3^ mol g _coal_^–1^ s^–1^ at 900 °C) was higher than the maximum CO_2_ rate estimated by O_2_ equilibrium concentration, indicating that direct reaction between volatiles and metal oxides was possible.

**Figure 6:**
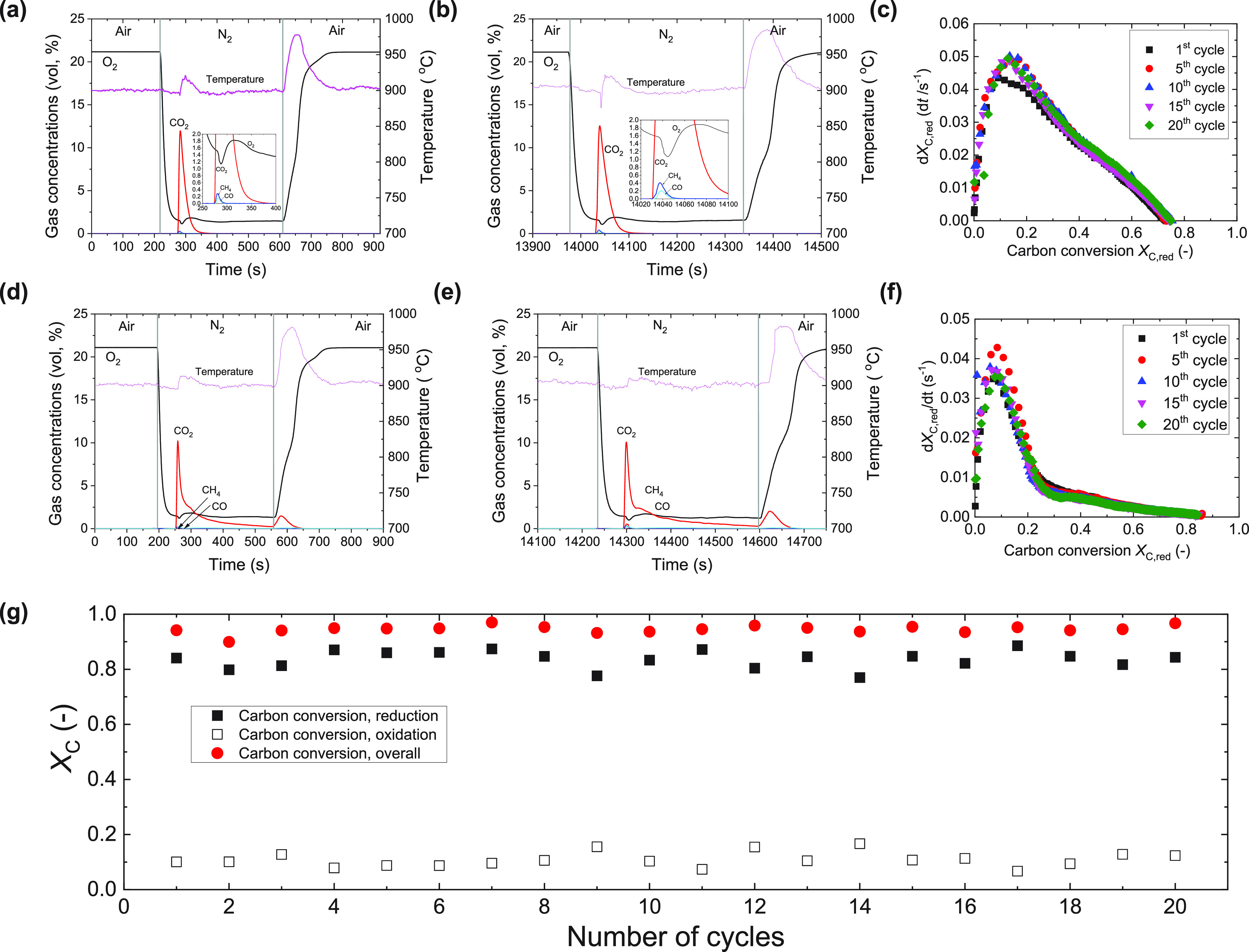
Reactivity and stability of oxygen carriers during coal combustion. (a and b) Profiles of combustion of lignite at a set-point temperature of 900 °C of the (a) first and (b) 20th redox-cycles. (d and e) Profiles of combustion of bituminous coal at a set-point temperature of 900 °C of the (d) first and (e) 20th redox-cycles. The vertical dashed lines indicate the switch of the fluidizing gases. The insets in (a and b) show a magnified profile of gas concentrations during the initial stage of volatiles combustion. The ordinate and abscissa scales of the insets are vol % and s, respectively. (c and f) Rate of carbon conversion (d*X*_C_, _red_/d*t*) versus carbon conversion *X*_C_,_red_ at typical cycles of combustion of (c) lignite coal and (f) bituminous coal at 900 °C. (g) Overall carbon conversion (*X*_C_) over 20 redox-cycles during the combustion of bituminous coals at 900 °C. The carbon conversions labeled as “reduction”, “oxidation”, and “overall” correspond to the conversion of carbon during the oxygen release stage, oxidation stage, and the combined value for each cycle, respectively.

We also demonstrated that the Cu-based oxygen carriers are highly active and stable over multiple cycles of solid fuel combustion. Detailed combustion profiles are shown in Figure 6. The gas concentration profiles at the first and 20th cycles for the combustion of lignite (Figure 6a,b) and bituminous coal (Figure 6d,e) were almost identical. The rate of carbon conversion for lignite and bituminous coal showed a high reproducibility over 20 cycles (c.f., Figure 6c,f). Due to non-ideal mixing between the char and the oxygen carriers, and the low reactivity of the bituminous coal char, some unburned carbon was carried over to the oxidation stage. The overall carbon conversion (*X*_C,total_) of bituminous coal in each cycle was stable at around 0.95 (Figure 6g). The less-than-unity carbon conversion may be attributed to the bypassing of volatiles. The reactivity of the oxygen carriers to release and take-up oxygen, after coal combustion, was found to be identical with the fresh materials, as shown in Figure 4. These results confirmed the high stability of the Cu-based oxygen carriers.

### 2.4 Chemical Looping Combustion in the Presence of Steam for Gasification

**Figure 7:**
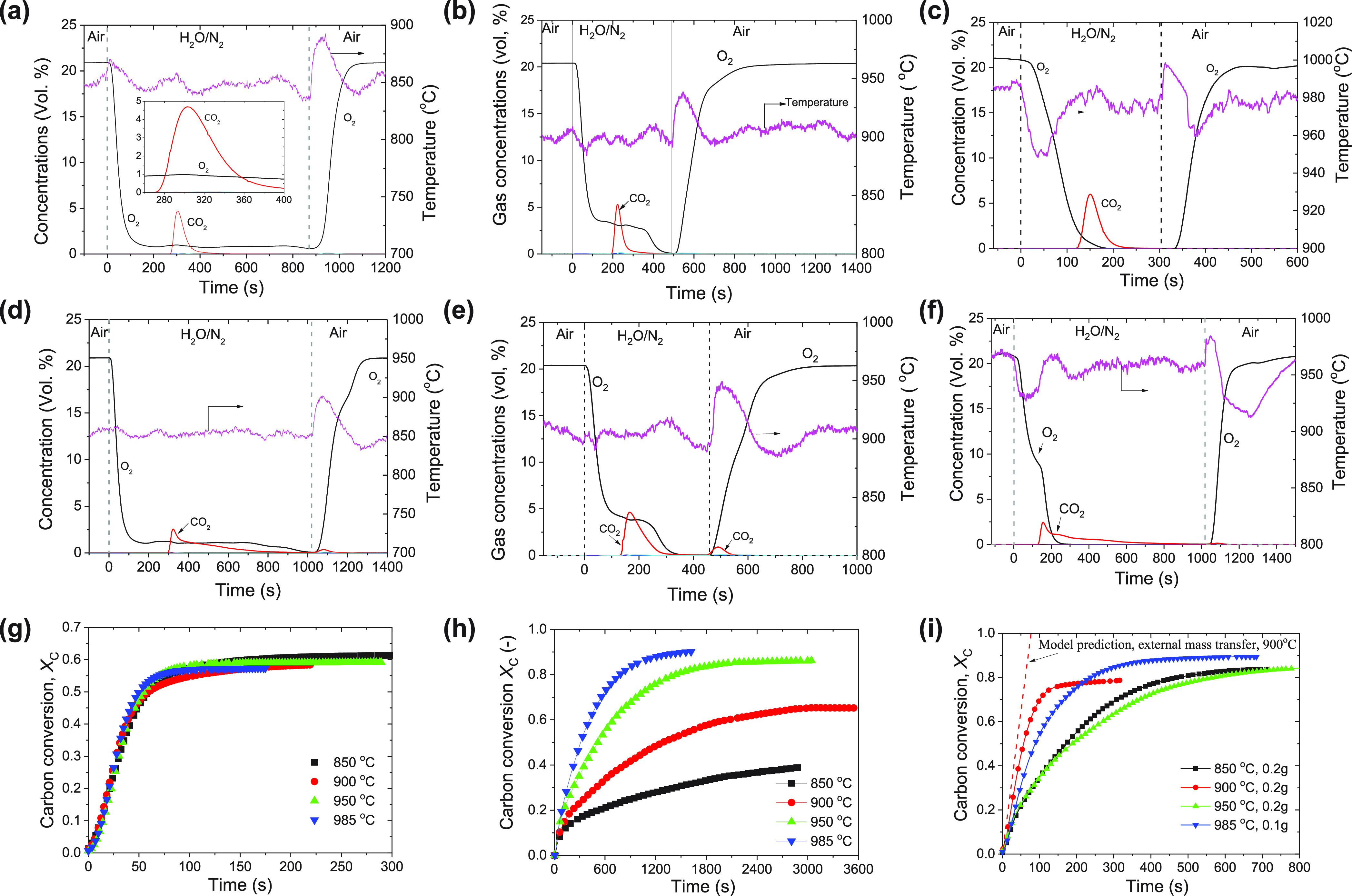
Combustion of solid fuels in the presence of steam. Concentration profiles of 0.2 g (a–c) Hambach lignite char combustion and (d–f) bituminous coal combustion (a–f) with 20 g of oxygen carriers fluidized by 25.6 vol % H_2_O balanced with N_2_ at (a and d) 850 °C, (b and e) 900 °C, and (c and f) 985 °C. The flow rate of H_2_O was 49 mL/h (at SATP) and was balanced with 53.6 cm^3^/s of N_2_ (at SATP), which corresponds to 25.6% H_2_O/N_2_ and a total flow of 72.0 cm^3^/s. During the oxidation stage, air with a flow rate of 50.9 cm^3^/s (at SATP) was used. (g–i) Carbon conversion in the temperature range 850–985 °C for (g) combustion of lignite, (h) gasification of bituminous coal in steam, and (i) combustion of bituminous coal in the presence of 20 g of oxygen carriers and steam.

In the previous experiments, solely N_2_ was used as the fluidizing gas to simplify the interpretation of the experimental results. However, in scaled-up processes, the fluidizing gas would be free of N_2_. When steam and/or CO_2_ are used for fluidization, they are likely to serve as gasifying agents (even) in the presence of oxygen carriers. The gasification products would either react with the CuO, with the gaseous O_2_ released by the decomposing CuO or, after complete decomposition, with Cu_2_O (for further reduction to Cu). A deep reduction of the oxygen carriers might result in agglomeration or sintering, which metallic Cu is prone to sintering and agglomeration. Therefore, we also performed the combustion of coal in the presence of steam. The detailed gasification reaction and CLC profiles are shown in Figures S13–S18. In the iG-CLC experiments, we increased the temperature of the bed step by step from 850 to 985 °C, and at each temperature step, we studied combustion of lignite, lignite char, and bituminous coal. Figure 7a shows a typical profile of the combustion of lignite in the presence of O_2_ released from the oxygen carriers and steam in the fluidizing gas. After the fluidizing gas was switched from air to 25.6 vol % H_2_O/N_2_, the oxygen carriers started to release oxygen and the exhaust composition of O_2_ in dry-basis reached a mole fraction of 0.78%. This corresponded to an O_2_ volume fraction of 0.56% in the bed, approximately equal to the O_2_ equilibrium fraction (0.46%). When a batch of 0.2 g of lignite was added to the bed, the pyrolysis gas and char were combusted to CO_2_ and H_2_O and the temperature increased slightly to 860 °C (due to the heat released from the combustion). After complete combustion of the lignite char, the oxygen carriers still released O_2_. When the inlet fluidizing gas was switched to air to reoxidize the oxygen carriers, a significant temperature increase to 890 °C was observed. The observed profiles of combustion of lignite at 850 °C (Figure 7a) and 900 °C (Figure 7b) were similar, except that the O_2_ fraction approached a stable value of ca. 3.0%. At these low temperatures, we did not observe defludization.

We also tested the oxygen carriers with lignite char and bituminous coal. The lignite char was tested in batch mode combustion similar to that of lignite and generally gave similar results (Figure S16). When testing the combustion of Taldinskaya bituminous coal, the reactivity was found to be significantly lower than that of lignite and thus the char produced would accumulate in the bed, similar to that observed in the CLOU process. As shown in Figure 7d, the combustion of the bituminous coal at 850 °C was fast for the first 60 s due to the rapid devolatilization and combustion of pyrolysis gas by oxygen released in the bed and freeboard area. The combustion rate of char was slow mainly due to the low oxygen concentration (limited by the O_2_ equilibrium). At 900 °C (Figure 7e), the combustion was completed within 150 s, even before the release of O_2_ was complete. During the following oxidation period, a small amount of CO_2_ (83 mmol, corresponding to *X*_C,ox_ = 7.5%) was released, suggesting that the char generated from the raw coal was not gasified by the gasification agent at 900 °C, as evidenced by no detection of CO or H_2_. Therefore, the further combustion of char with Cu_2_O did not occur.

With the temperature increased to above 950 °C, the rate of decomposition of CuO to Cu_2_O significantly increased and consequently the loss of oxygen during the N_2_ purging period also increased. As shown in Figure 7c, at a temperature of 985 °C, the fraction of O_2_ decreased to a value close to zero within 150 s after gas switching. The lignite added to the bed was completely burned mainly *via* the gasification intermediate step, that is, the gasification products were oxidized by the solid phase oxygen in the oxygen carrier to CO_2_ and H_2_O. During this stage, the Cu-based oxygen carriers were further reduced to Cu. Therefore, in the subsequent oxidation stage (of the first redox-cycle), defluidization occurred during the beginning of the oxidation period (and was confirmed visually). This was possibly due to a high degree of reduction to copper and an excessive temperature increase during subsequent oxidation in air (bed temperature increased to *ca.* 1000 °C), with the surface temperature of the oxygen carriers potentially being even closer to the melting point of metallic copper (*ca.* 1085 °C). Such a significant temperature increase likely led to partial agglomeration of particles and defuidization and partial sintering of the oxygen carrier. Fortunately, the defluidization did not lead to significant bed agglomeration, as fluidization commenced when adding 0.1 g of coal in the subsequent combustion phase, and defluidization was not observed again thereafter. The oxygen carriers recovered after the experiment were found to be segregated (not agglomerated). During the initial oxidation stage, the fraction of oxygen increased in the off-gas fast suggesting a fast oxidation rate of Cu to Cu_2_O. During the later period, the slow increase of O_2_ concentration suggested a slower oxidation rate, which could be due to a smaller concentration difference between the feed gas and the equilibrium.

At higher temperatures, the role of steam in the combustion became important because the combustion pathway varied from direct combustion by gaseous oxygen to indirect combustion *via* the gasification intermediates. Figure 7f shows a typical combustion of bituminous coal at 950 °C *via* both CLOU and iG-CLC. When the O_2_ released in the bed was depleted, the rate of combustion shows a significant drop during the later iG-CLC step. Nevertheless, the residual coal char was fully burned as the syngas generated *via* gasification of char was completely oxidized by the oxygen carrier.

The reaction kinetics of combustion of solid fuels were analyzed and are presented in Figure 7g–i and Figures S18 and S19. The reaction rates of combustion were certainly much higher than that of gasification. For reactive lignite and lignite char, the carbon conversion rates were limited by external mass transfer of the O_2_ transferred from the oxygen carriers into the bed. In the case of bituminous coal, the initial combustion rate was limited by the O_2_ release. However, at a later stage, the reaction rate was slower as the O_2_ had been fully released from the oxygen carriers, and the slow gasification of coal char became the rate-limiting step. It should be noted that these experiments were performed with diluted steam in N_2_. In future work and practical application, pure steam or recirculated flue gas (CO_2_ and H_2_O) from the fuel reactor could be used as fluidizing gases and gasification agents.

### 2.5 Chemical-Looping Combustion of Gaseous Fuels

**Figure 8:**
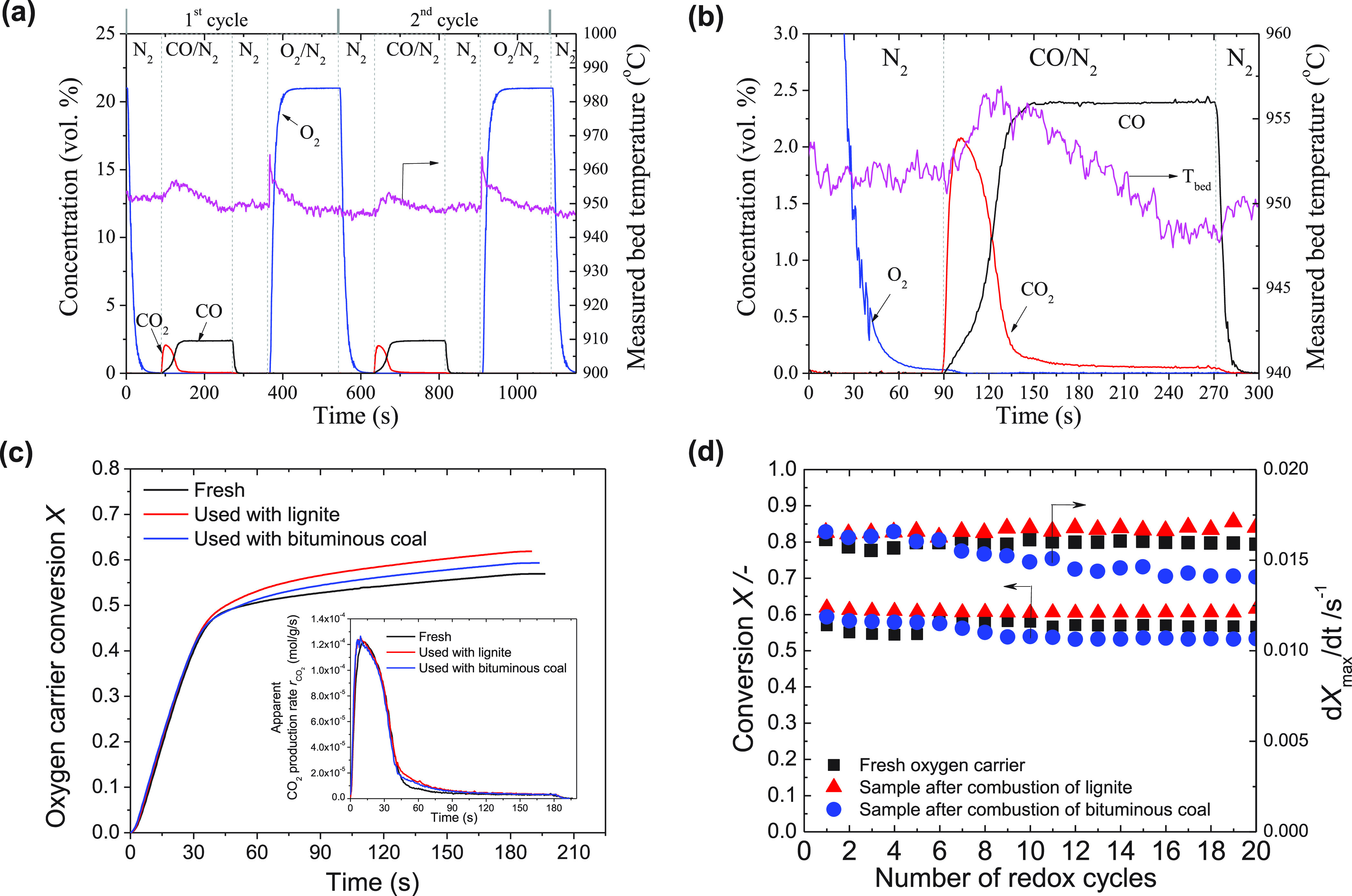
Chemical-looping combustion of gaseous fuels. (a and b) Gas concentration and measured bed temperature profiles of chemical-looping combustion with 0.5 g of oxygen carriers at a set-point temperature of 950 °C with ∼2.4 vol % CO in N_2_ (total flow rate of 65.8 mL s^–1^, STP, 180 s). (a) First two redox cycles of freshly calcined oxygen carriers. The vertical dashed lines indicate the switch of inlet gases. (b) Enlargement of the first reduction of fresh oxygen carriers. (c) oxygen carrier conversion versus time during the first cycle. Inset shows the apparent CO_2_ production rate as a function of time. (d) Oxygen carrier conversion and maximum rate of reduction of fresh and cycled oxygen carriers over the number of redox cycles.

We further examined the stability of oxygen carriers by exposing them to harsher operation conditions, relevant for chemical-looping combustion with gaseous fuels. Gas concentration profiles of typical cycles are shown in Figure 8a,b. The CuO decomposed to Cu_2_O, releasing O_2_ during the N_2_ purging period. This was followed by reduction to Cu in the presence of a gaseous fuel (CO) balanced with N_2_. After another inert phase (using N_2_ for fluidization), the oxygen carriers were regenerated back to CuO in air. In the reduction period, the reaction was limited by external mass transfer. The oxygen carrier conversions were calculated from the amount of CO_2_ generated during the CO reduction period divided by the total amount of oxygen in the oxygen carriers in their fully oxidized state. The resulting oxygen carrier conversions and rates were limited by residual lattice oxygen in the oxygen carriers. Oxygen carriers recovered after multiple redox-cycles of coal combustion were further tested during redox-cycling with the gaseous fuel. The Cu-based oxygen carriers maintained a high oxygen storage capacity during the redox cycling (Figure 8d). After CLOU combustion of coal, the reducible oxygen capacity from CuO to Cu was ∼11 wt % (of the total oxygen carrier mass), as measured by temperature-programmed reduction (TPR; Figure 4C). During the redox-experiments with CO/N_2_, only 50–60% of this O_2_ storage capacity was observed, which was mainly due to the O_2_ release during the inert period (i.e., decomposition of Cu to Cu_2_O). Agglomeration and defluidization were not observed for all samples during the reduction and oxidation stages.

### 2.6 Characterization of Cycled Materials

**Figure 9:**
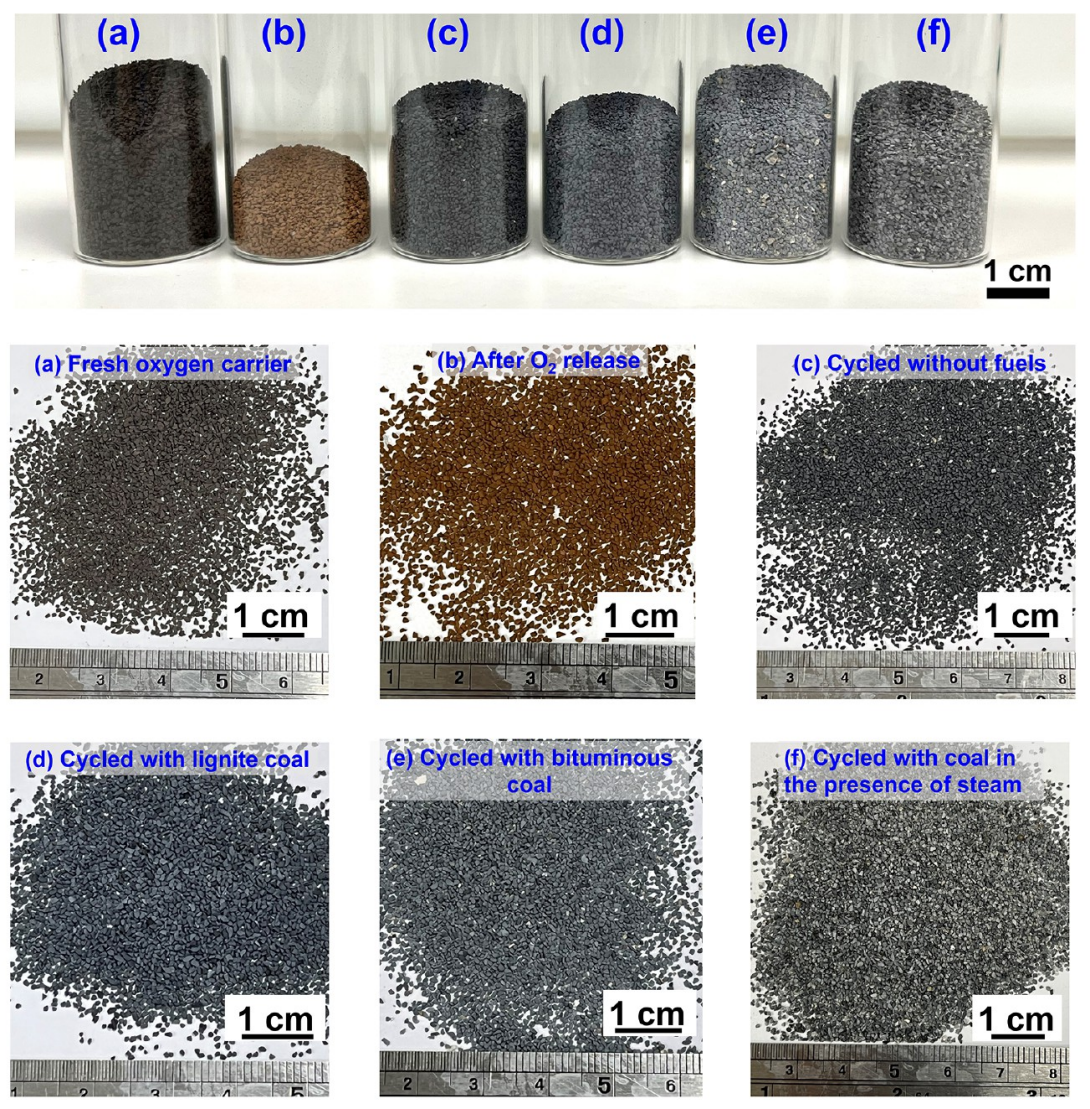
Photos of fresh and cycled oxygen carrier materials: (a) fresh oxygen carrier; (b) after O_2_ release in the fluidized bed; (c) after 20 cycles of oxygen release and storage at 900 °C in fluidized bed; (d) after 20 cycles of CLOU combustion with lignite at 900 °C; (e) after 20 cycles of CLOU combustion with bituminous coal at 900 °C; and (f) cycled oxygen carriers recovered from iG-CLC combustion of coal at 850–985 °C in the presence of steam. Bulk agglomeration between particles was not observed in all samples.

For copper-based oxygen carriers, the most important concern is the ability to resist sintering and agglomeration during redox cycles. Owing to the low melting point of copper, sintering of reduced copper and adhesion of metallic copper on the particle surface could have a promotive effect on particle agglomeration. Our nanostructured oxygen carriers consisted of highly dispersed copper nanoparticles stabilized by the support, which restricted the diffusion and migration of copper phases toward the surface of particles. Therefore, the oxygen carrier materials demonstrated high resistance to sintering and agglomeration. Figure 9 shows photos of fresh (a) and cycled oxygen carrier particles after testing in both CLOU and iG-CLC cycles in fluidized bed reactors (b–f). It can be observed that the oxygen carrier particles did not agglomerate after exposed to O_2_ release (Figure 9b), CLOU cycles without fuels (Figure 9c), and 20 cycles of CLOU combustion with lignite (Figure 9d) and bituminous coal (Figure 9e) in the fluidized bed. In all of these samples, the particles remained discrete without agglomeration, and their geometry and sizes maintained roughly the same as fresh particles. It should be noted that the sample recovered from iG-CLC combustion of coal at 850–985 °C in the presence of steam was exposed to harsh operation conditions and might have experienced partial agglomeration within the reactors (Figure 9f). As described above, defluidization only occurred in one iG-CLC experiment at a very high temperature of 985 °C when a large batch of lignite coal was added to the bed in the presence of steam. The high degree of reduction to copper and subsequent oxidation in air resulted in to a significant temperature increase in the bed, which likely led to melting of copper and oxides, and consequently partial agglomeration of particles and defluidization and partial sintering of the oxygen carriers. After this cycle, we reduced the amount of coal added to the bed, and defluidization did not occur any more in the subsequent cycles. The oxygen carrier particles recycled from the reactor did not show agglomeration, but we speculate that partial agglomeration of particles might have occurred during the oxidation due to a significant temperature increase, but subsequent fluidization might have breakdown the agglomerated particles.

**Figure 10:**
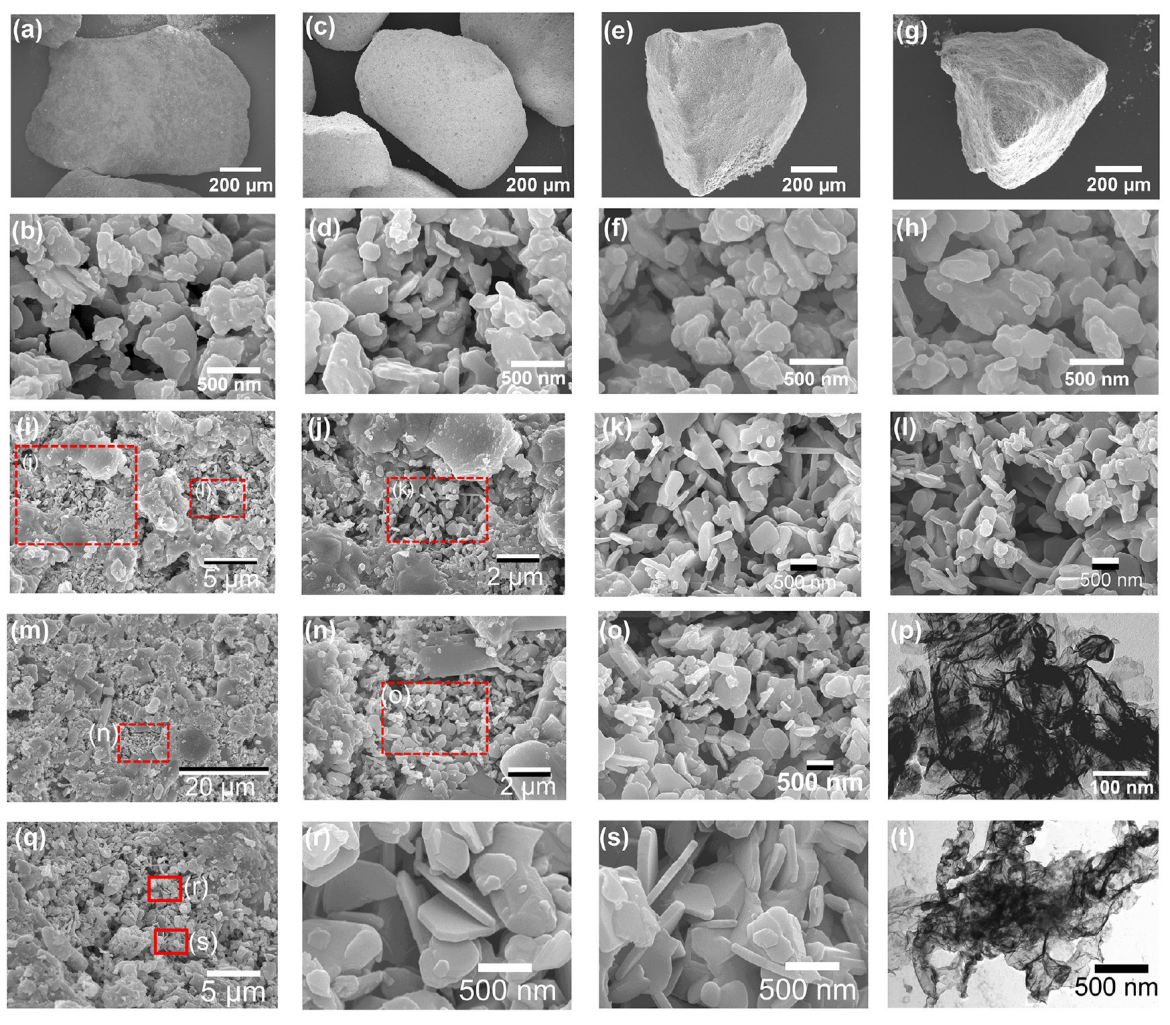
Morphology analysis of cycled oxygen carrier materials. (a and b) SEM images of oxygen carrier particle and surface after O_2_ release (Cu_2_O); (c and d) SEM images of (c) an oxygen carrier particle and (d) its surface after 20 CLOU cycles at 900 °C in the absence of a fuel; (e and f) SEM images of (e) a particle and (f) its surface after 20 cycles of CLOU combustion with lignite at 900 °C; (g and h) SEM image of (g) a particle and (h) its surface after 20 cycles of CLOU combustion of bituminous coal at 900 °C. (i–l) SEM images of cycled oxygen carriers after 20 redox cycles with gaseous fuel (CO/N_2_) for reduction and air as oxidant at set-point temperature of 950 °C. (m–p) SEM and STEM images of cycled oxygen carrier. The material was recovered from 20 cycles of CLOU combustion with coal and further exposed to 20 redox cycles with gaseous fuel (CO) and air as oxidant at set-point temperature of 950 °C. (q–s) SEM images and (t) STEM image of cycled oxygen carriers recovered from iG-CLC combustion of coal at 850–985 °C in the presence of steam.

To understand the evolution of the surface morphologies and microstructures of the oxygen carriers during the CLOU and redox cycles, we performed extensive characterization analyses of the cycled materials. The Brunauer–Emmett–Teller (BET) surface area of the cycled oxygen carrier particles, determined by N_2_ adsorption at 77 K, decreased from 5 m^2^ g^–1^ (as calcined) to about 2–3 m^2^ g^–1^ (after redox cycling) (Figure S20 and Table S3). Detailed high-resolution SEM images are provided in Figures S21–S24. At a macroscopic scale, the robust micron-sized particles were found to have maintained their sizes after decomposition to Cu_2_O (Figure 10a) and CLOU cycles (Figure 10c,e,g), showed good resistance to attrition in the fluidized bed, and no signs of agglomeration after multiple redox-cycles were observed. The nanoplatelet-like morphology was preserved after decomposition (Figure 10b) and CLOU redox cycling (Figure 10d,f,h). Compared to fresh samples, the grains became slightly larger due to sintering; yet, the shape and size of the grains did not change significantly over O_2_ release and storage cycles. Similar morphologies were observed for oxygen carriers cycled with reduction in CO/N_2_ (Figure 10i–l), cycled in CLOU experiments with coal with subsequent redox-cycles with CO/N_2_ (Figure 10m–p), as well as those cycled in iG-CLC in the presence of steam (Figure 10q–t). Large grains (∼5 μm) were formed in some regions due to sintering caused by the harsh operating conditions, while in some regions the nanoplate-like morphology (thickness of ca. 50–100 nm; lateral size of 500 nm) was preserved. These highly stable nanoplate-like microstructures might limit the sintering as well as facilitate a quick O_2_ release and uptake. STEM analysis (Figure 10p,t) also indicated that CuO crystals were still reasonably well-dispersed in the support.

To understand the structural changes of oxygen carriers during CLOU and iG-CLC cycling, we also performed XRD analysis. We examined the XRD pattern of cycled oxygen carriers after CLOU cycles (Figure 4D and Figure S25), after further exposure to redox-cycles with CO/N_2_ (Figure S26) and those recovered after iG-CLC cycling (Figure S27). CuO remained the dominant crystalline phase after redox cycling in both CLOU and CLC processes without observation of copper aluminate formation. This confirmed the high thermal stability of the oxygen carrier, which was stabilized by sodium–aluminum phases in the support.

The samples CLOU-cycled with solid fuels showed a stable reactivity and oxygen storage capacity (Figure 4). In contrast, the samples cycled in iG-CLC at higher temperatures and in the presence of steam showed a slight loss in their oxygen storage capacity. This was observed in the TGA and the fluidized bed reactor (Figure S28). Redox cycling between Cu_2_O and Cu in a fluidized bed reactor showed relatively stable oxygen storage capacity (Figure S29). In order to obtain the intrinsic reactivity of the oxygen carriers, temperature-programmed reduction (TPR) and oxidation (TPO) experiments were performed in TGA with a high gas/solid ratio to minimize the limitation by external mass transfer (Figures S30 and S31, Table S4). The lattice oxygen storage capacity decreased from 11.6 to 8.6 wt %. However, it was possible to partially reactivated it to 9.3% by exposing the cycled samples to further reduction by CO and oxidation in air. The weight change in the subsequent oxidation step was comparable to that of reduction, demonstrating that the oxygen release and storage is reversible and the oxygen carriers can be partially regenerated (though with a slightly decreased capacity). One possible reason was the strong interaction between CuO and support, e.g., the formation of copper aluminates, which should be reducible below 950 °C. Therefore, the loss of the oxygen storage capacity could likely be attributed to the thermal sintering of the oxygen carriers during the high-temperature operation, especially when the oxygen carriers experienced defluidisation during the first cycle at the highest temperature (as reported above). A significant loss of pore volume was confirmed by N_2_ adsorption analysis (Figure S32). These results suggested that there is a need to employ or develop support materials with a higher thermal stability, such as CuO supported on MgAl_2_O_4_.^62^ In addition, the operating conditions should be carefully controlled to avoid excessive reduction and significant temperature increase of the bed materials.

## 3 Conclusions

In summary, we report the use of Cu-based mixed oxides as oxygen storage materials for the combustion of solid fuels in CLC processes with a high CO_2_ capture efficiency. The nanostructured mixed metal oxides derived from Cu–Al LDH precursors, stabilized by sodium, proved to be highly thermally stable and reactive for the combustion of solid fuels as well as gaseous fuels during thermochemical redox-cycles (i.e., cycling between CuO-Cu_2_O and CuO-Cu). The high thermal stability was attributed to the high degree of dispersion of the active Cu phases in the support, which was a result of a high degree of dispersion of Cu and Al phases at the molecular level in the precursors. The sodium-containing phases stabilized the Cu phases in the support and inhibited the formation of copper aluminates. The oxygen carriers demonstrated a high gaseous oxygen release capacity (∼5 wt % of their total mass), oxygen storage capacity (∼11 wt % of their total mass), and high thermal stability over multiple redox cycles in both TGA and fluidized bed reactors.

The Cu-based oxygen carriers showed a high performance for the combustion of two types of coal. The rate of combustion of char was strongly related to the O_2_ partial pressure generated by the oxygen carriers. At higher temperatures, the rate of combustion of char was significantly enhanced. In extended cycling experiments with two solid fuels (a lignite and a bituminous coal), the oxygen carriers maintained their capacity to take-up and release oxygen over 20 cycles. The oxygen carriers also showed stable reactivity during cycling between CuO and Cu over multiple redox-cycles with gaseous fuel and solid fuels in iG-CLC conditions. Slight deactivation and sintering of oxygen carriers occurred after the operation at iG-CLC at a very high temperature of 985 °C. We demonstrated that these high-performance oxygen storage materials were very promising for the combustion of solid fuels while allowing for efficient CO_2_ capture. We anticipate that our strategy of synthesizing the oxygen carrier materials would inspire rational design of novel oxygen storage materials for a wide range of thermochemical processes for clean energy production.

## Supporting Information Available

Discussions of experimental methods, figures of photo of co-precipitation of Cu-Al hydrotalcite precursor, schematic diagram and photo of the fluidized bed reactor setup, FTIR spectra, thermogravimetric analysis, photos of oxygen carrier particles, HRTEM images and calculation of lattice fringe spacings, thermodynamic analysis, N_2_ adsorption isotherms, profiles of combustion of lignite and bituminous coal at different temperatures, rate of CO_2_ production as a function of carbon conversion, plot of rate of CO_2_ production as a function of set-point temperature, gasification profiles, Arrhenius plots, combustion profiles in the presence of oxygen carriers, rate of combustion of coal expressed as rate of production of CO_2_ as a function of carbon conversion, carbon conversion of coal in gasification and iG-CLC, pore size distribution, SEM images, XRD patterns, O_2_ release and storage in fresh and cycled oxygen carrier, CLC of gaseous fuel with fresh and used oxygen carriers, temperature programmed reduction and oxidation of Cu-based oxygen carrier, and fitting of TPR and TPO rate curves, and tables of proximate and ultimate analyses of coal samples, ICP-MS analysis of coal ash, fresh and used oxygen carrier materials, pore size properties of fresh and used oxygen carriers, and quantitative fitting of the peaks in TPR and TPO of oxygen carrier (PDF)
