# Supplementary material for: Hydrotalcite-Derived Copper-Based Oxygen Carrier Materials for Efficient Chemical-Looping Combustion of Solid Fuels with CO2 Capture
Source: Energy Fuels. 2022 Aug 26;36(18):11062–76. doi: 10.1021/acs.energyfuels.2c02409 (PMC9483923; doi:10.1021/acs.energyfuels.2c02409)
Supplement: Supplementary file 1 — ef2c02409_si_001.pdf [file ef2c02409_si_001.pdf]

Supporting Information for

Hydrotalcite-Derived Copper-Based Oxygen Carrier Materials for Efficient Chemical-Looping Combustion of Solid Fuels with CO_2 Capture

*Michael High[a], Clemens F. Patzschke[a], Liya Zheng[a,b], Dewang Zeng[c], Rui Xiao[c], Paul S. Fennell[a], Qilei Song[a]**

[a]*Department of Chemical Engineering, Imperial College London, London SW7 2AZ, UK. Email:* q.song@imperial.ac.uk

[b] *School of Materials, Sun Yat-sen University, Guangzhou, 510275, PR China.*

[c]*MOE key Laboratory of Energy Thermal Conversion and Control, School of Energy and Environment, Southeast University, Nanjing, 210096, China.*

Experimental Methods

1. Synthesis of LDH precursor and calcination

The Cu-Al-LDH precursor was prepared by co-precipitation using the constant pH method at room temperature, following method reported in a previous study.[1] A photo of the coprecipitation process is shown in **Figure S1**. An aqueous solution of $Cu(NO_3)_2 \cdot 2.5H_2O$ and $Al(NO_3)_3 \cdot 9H_2O$ (1 M of each) was added to a flask under vigorous stirring, with simultaneous addition of another aqueous solution of NaOH and Na_2CO_3 (1 M of each), at a constant pH of 9.6±0.1 in the precipitate mixture. After aging for 2 h at room temperature, the precipitate was washed with purified water for five times, filtered and dried in an oven at 60 °C for 48 h in air. The LDH precursor was then calcined in a muffle oven at 950 °C for 5 h. After cooling down to room temperature, the powders were crushed and sieved to a size range of 500-710 μm.

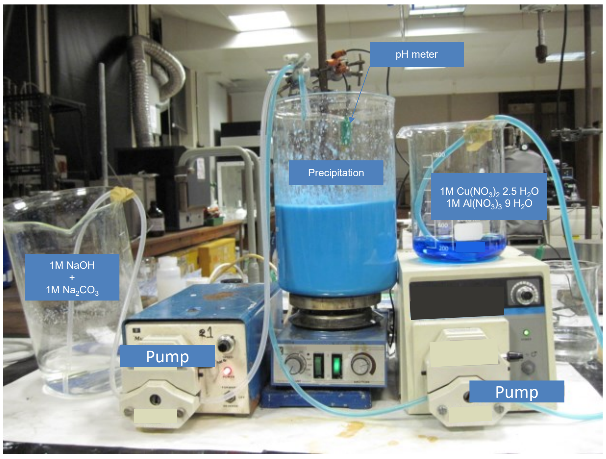

Figure S1. Photo of co-precipitation of Cu-Al hydrotalcite precursor. The metal salt solution ($Cu(NO_3)_2 \cdot 2.5H_2O$ and $Al(NO_3)_3 \cdot 9H_2O$) and precipitating agent solution (NaOH and Na_2CO_3) are pumped into the beaker, with pH controlled at 9.6. The resulting precipitate was washed and dried in oven, and calcined to obtain the Cu-based mixed metal oxides.

2. Characterization

The composition of the fresh and used oxygen carriers were determined by an inductively coupled plasma mass spectrometer (ICP-MS) (ELAN DRC II, PerkinElmer Inc., USA). The content of copper and sodium were determined separately using inductively coupled plasma atomic emission spectroscopy (ICP-AES, Varian Liberty) calibrated with standards. X-ray diffraction (XRD) was carried out with a Philips PW1830 HT Generator operated at 40 mA and 40 kV using Cu Kα radiation with a step of 0.025° per second. Scanning electron microscopy (SEM) and scanning transmission electron microscopy (STEM) were performed using a microscope (Hitachi S5500). High resolution TEM was carried out using a Philips Tecnai 20 transmission electron microscope. Nitrogen adsorption isotherms were measured at 77 K with Micromeritics instrument ASAP 2020.

3. Thermal analysis

Temperature programmed reduction (TPR) was carried out in a thermogravimetric analyser (TGA) (Mettler Toledo Inc.). A ~5 mg batch of oxygen carriers was heated from 120 °C to 950 °C at a heating rate of 5 °C min^{-1} in 5.0 vol% H_2/ N_2 (50 mL min^{-1}, STP).

CLOU and CLC redox cycling tests were carried out in a Q5000 (TA Instruments) to evaluate the stability of the metal oxides. In a typical experiment, 3–4 mg of the calcined mixed oxides were placed in a platinum crucible (1.5 mm high and 9.8 mm in diameter) and cycled for 100 redox cycles. In CLC cycling, the materials heated up to 900°C in air, then exposed to N_2 purging for 1 min, and then exposed to (1) reducing gas of 10 vol% CO/N_2 for 3 min, followed by (2) N_2 purging for 1 min, and (3) air oxidation for 8 min, and (4) 1 min purging with N_2. The four-step sequence was repeated for a total of 100 cycles. In CLOU cycling, the materials were exposed to decomposition in N_2 for 4 min and oxidation in air for 6 min. All experiments were performed at 900°C, and the total gas flow rate was kept constant at 180 ml min^{-1} (SATP). The oxygen release capacity (CLOU) and oxygen storage capacity (CLC) were derived from the weight changes during redox cycles, specifically from the difference of the relative weights at the beginning and the end of the decomposition and reduction period, respectively.

4. Fluidized bed reactor

The redox cycling of metal oxides were investigated in a laboratory fluidized bed (**Figure S2**). The reactor (i.d. 29.5 mm; length 460 mm) was made of quartz with a sintered porous quartz plate as a gas distributor located 110 mm from the bottom. The reactor was heated by an electric furnace and the temperature of the bed was controlled by a PID controller connected to a K-type thermocouple situated axially in the bed 15 mm above the distributor. A second K-type thermocouple was placed at the same position to log temperature continuously. The inlet gases were supplied from gas cylinders (BOC Ltd.) and metered to the bed by rotameters. The gas supplied to the reactor was switched by several programmed solenoid valves.

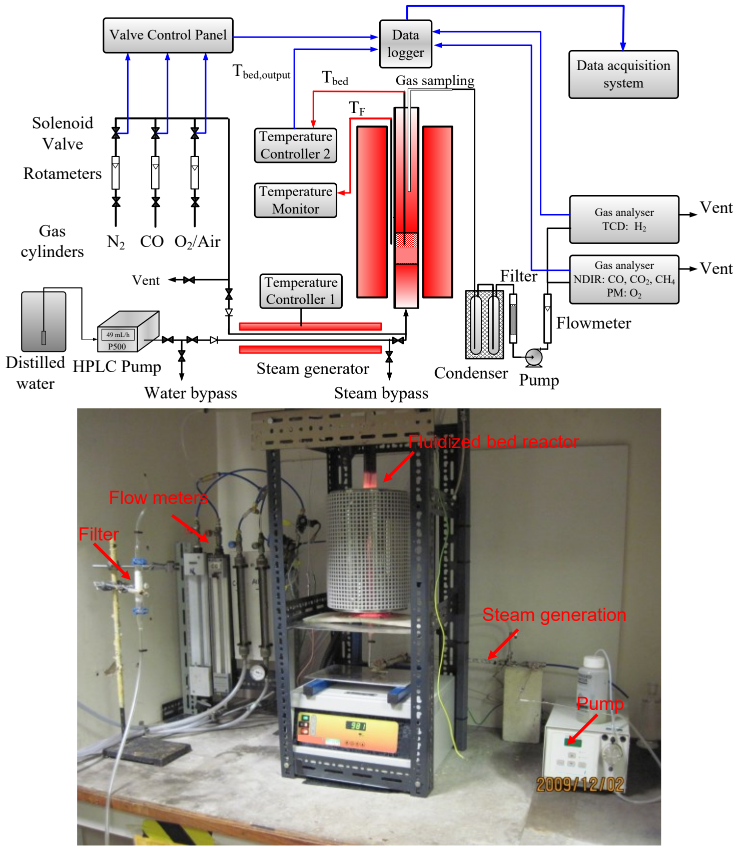

Figure S2. Schematic diagram (top) and photo (bottom) of the fluidized bed reactor setup. NDIR: non-dispersive infrared analyser; PM: paramagnetic analyzer; TCD: thermal conductivity detector.

The flue gas was sampled form a point 140 mm above the gas distributor *via* a quartz sampling probe and desiccating tubes containing $CaCl_2$ with quartz wool as a filter at both ends. The resulting dry gas was introduced to two continuous gas analyzers placed in parallel (0.5 L min^{-1}, at 293 K and 1 atm) passing to each. The first analyzer (ABB EL3020) measured [CO_2], [CH_4], [CO] (via non-dispersive infrared (NDIR)) and [O_2] (using a paramagnetic method); the detection limit was 0.01 vol.%. The second analyzer (ABB EL3020) measured the mole fraction of H_2 using the principle of thermal conductivity (TCD). The concentrations were recorded at a frequency of 0.5 Hz in most experiments. The time delays of gas analyzer due to mixing in the sampling system were corrected.

5. Cyclic oxygen release and storage

A batch of oxygen carriers (15.0 g, in fully oxidized state) was alternately exposed to inert gas (N_2, 50.0 mL s^{-1}, STP) for 360s and oxidizing gas (air, 47.4 mL s^{-1}, STP) for 360-720 s in the laboratory-scale fluidized bed at a constant temperature (800-1000 °C). In some experiments, complete decomposition was performed by extending the time for N_2 purge and the ultimate O_2 uptake capacity was quantified. The stability of the oxygen carriers was evaluated by the apparent rate and overall amount of gaseous O_2 released over multiple cycles.

6. Chemical-looping combustion of solid fuels

Two solid fuels were used, a lignite (Hambach) and a bituminous coal (Taldinskaya). The proximate and ultimate analyses are presented in **Table S1**. The coal was sieved to a size range of 1.0 – 1.7 mm. A batch of oxygen carrier (15.0 g) were alternately exposed to inert gas (N_2, 50.0 mL s^{-1}, STP) and oxidizing gas (air, 47.4 mL s^{-1}, STP) in the fluidized bed at a constant temperature (800-1000 °C). At each O_2 release stage, a batch of coal (~0.2 g) was added to the reactor for combustion. Multiple cycles of coal combustion were also performed in this batch mode.

Table S1. Proximate and ultimate analyses of coal samples: moisture (M_{ad}), volatile matter (V_{ad}), ash content (A_{ad}), fixed carbon (FC_{ad}), and ultimate analyses (C_{ad}, H_{ad}, O_{ad}, N_{ad}, and S_{ad}). The received lignite was dried in air to remove the intra particular water (~54 wt%).

Sample	Proximate analysis (ad, wt%)				Ultimate analysis (ad, wt%)				
	M_{ad}	V_{ad}	A_{ad}	FC_{ad}	C_{ad}	H_{ad}	O_{ad}^{a}	N_{ad}	S_{ad}
Hambach lignite	18.0	41.0	25.87	37.5	54.87	4.32	18.33	0.74	0.24
Hambach lignite char	-	-	8.99	91.01	84.2	1.07	4.64	1.10	0
Taldinskaya coal	9.2	32.7	13.4	44.7	66.10	4.9	3.98	2.0	0.42

[a] Calculated by difference; ad: air dried basis

Two sets of experiments were performed. In the first set, the oxygen carrier materials were exposed to consecutive cycles at successive temperatures of 800, 850, 900, 950, and 985 °C using different

fuels at each temperature. At each temperature, multiple cycles of O_2 uptake and release were performed. For each cycle, one batch of coal particles was added to the bed during the O_2 release stage. Detailed procedure is as follows: a batch of 15 g of oxygen carrier particles (in fully oxidized state) was added to the bed, without other inert bed materials. Then the bed was heated to the desired reaction temperature and fluidized by air (47.4 mL s^{-1}, STP). Then the inlet gas was switched to a flow of N_2 (50.0 mL s^{-1}, STP) to purge the O_2 from the system. After about 60 s, a batch of 0.2 g coal particles, sieved to 1.0 – 1.7 mm was added to the bed. The minimum fluidization velocity was calculated using the equation developed by Wen and Yu.[2] The U_{mf} of the oxygen carriers was around 0.10 m s^{-1}, with the U/U_{mf} of oxygen carriers varying between 2.8 and 3.2 over the operation temperature of 800-985 °C. The calculated U/U_{mf} of the raw coal particles was 1.6 – 2.0, suggesting reasonable mixing of the coal particles with the oxygen carriers in the fluidized bed. Following the addition of the batch of fuel, when the mole fractions of CO, CO_2 and H_2 had fallen to the limit of detection of the analysers, the fluidizing gas was switched to zero grade air (47.4 mL s^{-1}, STP) for 6 min or extended to 12 min over temperature range of 950-985 °C until the O_2 concentration in the effluent gas reached the same value as that in the inlet gas. After oxidation, the bed was purged with pure N_2 before the oxygen carriers were exposed to the next cycle of reduction and oxidation. Each type of coal was tested for 3-5 cycles before the bed temperature was increased for experiments at a higher temperature. It was shown that the materials were stable over these high temperature cycles, therefore, the kinetics data of coal combustion could be compared. Further experiments using fresh particles at each temperature also gave the same combustion profiles.

In a further set of experiments, a fresh batch of oxygen carriers was exposed to 20 consecutive cycles of O_2 uptake and release at 900 °C and on each O_2 release cycle, 0.2 g of lignite was added to the bed. Using the same procedure, another batch of fresh oxygen carriers was tested with 20 cycles of bituminous coal at 900 °C. In both series of experiments, complete decomposition of the oxygen carriers was carried out before and after the 20 cycles, to examine the oxygen releasing capacity.

Chemical looping combustion in the presence of steam.
The combustion with solid fuels was carried out in the fluidized bed in cyclic batch mode. A batch of 20 g 60 wt% coprecipitated CuO/Al_2O_3 oxygen carrier particles were added to the bed. A batch of 10 cm^3 alumina sand (300-425µm) was also added to the bed. Then the bed was heated to desired reaction temperature in air (50.9 cm^3/s, STP). Then the inlet gas was switched to a steam/N_2 mixture (N_2 of 53.6 cm^3/s, STP and H_2O of 49 mL/h). The molar fraction of steam corresponds to 25.6 mol% in N_2. Pure steam was not used since a certain amount of carrier gas was necessary to pass through the system. After around 1-5 min, batches of 0.1-0.2 g coal particles with a size range of 1.0-1.7 mm were added to the bed using a test tube. A lower mass of coal (0.1 g) was used at higher temperatures because defluidisation occurred when too much coal was added. In most experiments the fluidizing gas during reduction was mixture of steam/N_2. During the combustion stage, the temperature was varied from 850-985 °C, and the U_{mf} of the oxygen carriers was around 0.11 m/s, with the U/U_{mf} of oxygen carriers approximately equal to 4 using steam/N_2 mixture as the fluidising gas, and 3 when using N_2 alone. The oxygen carrier particles were exposed to consecutive cycles at different temperatures of 850, 900, 950, and 985 °C. At each temperature, several batches of different solid fuels were tested, after which the temperature was increased to the next temperature. When the cyclic tests were finished the inlet gas was switched to air and the heater was shut down. The oxygen

carrier particles were cooled in the nitrogen flow to room temperature and collected for further testing.

Gasification of coal in fluidised bed

Control experiments of solid fuels gasification over inert bed were also performed in the fluidized bed reactor using 20 cm^3 alumina sand (300-425 μm) as bed material. Rapid pyrolysis and gasification of lignite and bituminous coal were carried out over a temperature range 850-985 °C. In brief, the inert bed materials were fluidized by the steam and N_2 mixture, a batch of 0.2 g of coal particles with size range of 1.0-1.7 mm was dropped into the fluidized bed. Rapid devolatilization occurred in the steam and nitrogen mixture and then the derived char was gasified to syngas. The residual char was oxidized when the feed gas was switched to air.

During the reactivity test with gases, the oxygen carrier conversion, defined as the ratio of the measured number of moles of CO_2 produced to the number expected from the complete reduction of the given mass of CuO in the carrier, was used to check the mass balance of O_2.

$$X_{\mathrm{OC,red}} = \frac{M_{\mathrm{CuO}}}{m_{\mathrm{ox}} y_{\mathrm{CuO}}} \int_0^t \dot{n}_{\mathrm{out}} y_{\mathrm{CO_2}} dt \qquad \text{(Equation S1)}$$

Where $y_{\mathrm{CO_2}}$ is the molar fraction of CO_2 in the product gas,

Since the reduction duration was long enough to completely reduce the oxygen carriers, the variation of oxygen carrier conversion could be used to determine the oxygen carrier capacity during cycling.

The carbon conversion, X_C was calculated from the total amount of gaseous species containing carbon, (CO, CO_2, and CH_4), divided by and the amount of carbon in the coal in the ultimate analysis (Table S1):

$$X_{\mathrm{C,red}} = \frac{\int_0^t \dot{n}_{\mathrm{out}} (y_{\mathrm{CO,out}} + y_{\mathrm{CO_2,out}} + y_{\mathrm{CH_4,out}}) dt}{n_{\mathrm{C,coal}}} \qquad \text{(Equation S2)}$$

where $n_{\mathrm{C,coal}}$ is the amount of C in the batch of coal added to the bed in mol, calculated based on the carbon content, $n_{\mathrm{C,coal}}=\mathrm{m_{coal}}C_{\mathrm{ad}}/12$ (mol). The rate of carbon conversion $dX_{\mathrm{C,red}}/dt$ (s^{-1}) was calculated as:

$$\frac{dX_{\mathrm{C,red}}}{dt} = \dot{n}_{\mathrm{out}} (y_{\mathrm{CO,out}} + y_{\mathrm{CO_2,out}} + y_{\mathrm{CH_4,out}}) \qquad \text{(Equation S3)}$$

Since the amount of CO and CH_4 is negligible, the combustion rate of coal could be expressed as a specific rate of production of CO_2 (mol g_{coal}^{-1} s^{-1})

$$r_{\mathrm{CO_2}} = \frac{C_{\mathrm{ad}}}{12} \frac{dX_{\mathrm{C,red}}}{dt} \qquad \text{(Equation S4)}$$

In certain cycles, residual coal char was oxidized to CO_2 and CO during the air oxidation period, thus the additional carbon conversion during oxidation was calculated as:

$$X_{\mathrm{C,ox}} = \frac{\int_0^t \dot{n}_{\mathrm{out}} (y_{\mathrm{CO,out}} + y_{\mathrm{CO_2,out}}) dt}{n_{\mathrm{C,coal}}} \qquad \text{(Equation S5)}$$

Therefore, the total carbon conversion was given by $X_{\mathrm{C,total}} = X_{\mathrm{C,red}} + X_{\mathrm{C,ox}}$.

7. Chemical-looping combustion of gaseous fuel

A batch of alumina sand (20 mL, 300-425 μm) served as the principal bed material to which a batch of oxygen carrier material (0.5 g, in oxidized state) was added. The bed was heated to the desired reaction temperature (950 °C) and fluidized by air. Then the oxygen carrier particles were exposed to consecutive redox cycles with each cycle consisting of four stages: (1) purging with inert N_2 (50.0 mL s^{-1}, STP, 90 s), (2) reduction with ~2.4 vol.% CO in N_2 (total flow rate of 65.8 mL s^{-1}, STP, 180 s), (3) purging with inert N_2 (50.0 mLs^{-1}, STP, 90 s), (4) oxidation with zero grade air (47.4 mL s^{-1}, STP, 180 s). Both the fresh oxygen carriers and the samples recovered from the solid fuels experiments were exposed to 20 consecutive redox cycles. The stability of the oxygen storage materials weas evaluated by determining the apparent rate and overall amount of CO_2 over multiple redox cycles.

8. Kinetics of gasification

For lignite coal and its derived char, the content of coal ash was quite low, therefore, the gasification of the shrinking coal particle may be described with the shrinking core model with chemical-reaction control with the resistance of diffusion in the product layer neglected.

$$\frac{dX_C}{dt} = k_{app}(1 - X)^{-2/3} \qquad \text{(Equation S6)}$$

Where X_C is the carbon conversion, k_{app} is the apparent rate constant (s^{-1}), t is the time (s).

For steam gasification of coal char, generally the Langmuir-Hinshelwood (L-H) model is used to describe the kinetics:

$$k_{\mathrm{app}} = \frac{k_a P_{\mathrm{H_2O}}}{1 + k_{\mathrm{H_2}} P_{\mathrm{H_2}} + k_{\mathrm{CO}} P_{\mathrm{CO}} + k_{\mathrm{H_2O}} P_{\mathrm{H_2O}}} \qquad \text{(Equation S7)}$$

Where k_a is the reaction rate constant ($MPa^{-1}s^{-1}$), $P_{\mathrm{H_2O}}$ is the partial pressure of steam in the bed (MPa), $k_{\mathrm{H_2}}$ is the adsorption constant of H_2 (MPa^{-1}), $P_{\mathrm{H_2}}$ is the partial pressure of H_2 in the bed (MPa), $k_{\mathrm{H_2O}}$ is the adsorption constant of H_2O (MPa^{-1}).

Generally, En. S7 can be used to describe the kinetics of gasification in a wide pressure range. The inhibition of gasification by gasification product, *i.e.* H_2, depends on the operating conditions. In the fluidized bed, the weight ratio of coal to the bed material was substantially small and the partial

pressure of H_2 was much smaller than that of steam, so the inhibition effect may be neglected. Therefore, the L-H equation could be expressed as follows with the term of $k_{H_2}P_{H_2}$ neglected:

$$k_{app} = \frac{k_a P_{H_2O}}{1 + k_{H_2O} P_{H_2O}} \qquad \text{(Equation S8)}$$

This equation also works for the case when the gasification product was oxidised by oxygen carriers, removing the H_2 and CO, as presented in CLC of coal with iron-based oxygen carriers under pressurized condition.[3]

In this study the gasification of coal with and without oxygen carriers was performed in a fluidized bed at atmospheric pressure. During the gasification experiments, the partial pressure of H_2 was about 0.007 MPa at the maximum and it was much smaller than P_{H_2O} (0.0256 MPa at bulk). Under the low partial pressure of H_2O, the gasification of coal char in steam could be assumed as first order without consideration of the inhibition effect of CO and H_2. Therefore, the conversion of coal char with time could be described using a simple shrinking unreacted core model:

$$1-(1-X_C)^{1/3} = \frac{k_s C_{H_2O}}{\rho_C R_{PC}} t \qquad \text{(Equation S9)}$$

The carbon conversion of lignite and char versus time as shown in Fig. 5a and c were simulated with this equation. For the bituminous coal, the rate of devolatilization was much faster than that of gasification of the char generated *in situ*. Also the relatively large particle size of bituminous coal and examination of the Weisz-Prater criterion [4] indicates that the reaction was influenced to some extent by the internal mass transfer. Nevertheless, the parameters of chemical-reaction control could be derived using Eq. S9 with only the stage of gasification of char considered.

Assuming the reaction rate constant follows the Arrhenius equation:

$$k_s = k_0 \exp(-E_a / RT) \qquad \text{(Equation S10)}$$

The Arrhenius plots of reaction rate constant (k_s) versus 1/T is presented in Fig. S12. The value of the apparent activation energies (E_a) were calculated from the value of the slope of the linear fitting line. The apparent activation energies for gasification of lignite, lignite char and bituminous coal char were 57, 94, and 218 kJ/mol, respectively. For the gasification of lignite and lignite char, the apparent activation energies are slightly lower than those reported in the literature for steam coal gasification, indicating that external mass transfer of steam might influence the reaction. For the bituminous coal, the value of apparent activation energy corresponds well to those in literature.

8. Kinetic analysis of coal combustion without steam

Here, rates of O_2 release from the carrier are compared with the maximum rate of oxidation of the char, assuming it is controlled by external mass transfer. Thus, the maximum theoretical rate of

external mass transfer of O_2 between the bed and a particle of char is given by the rate of CO_2 production:

$$r_{CO_2} = k_{G,C}\pi d_{PC}{}^2 C_{O_2,b} \approx k_{G,C}\pi d_{PC}{}^2 C_{O_2,e} \text{ mol g}_{coal}^{-1}\text{ s}^{-1}. \quad \text{(Equation S11)}$$

Here, $C_{O_2,b}$ is the bulk O_2 concentration in the bed, mol m^{-3}, assumed to be equal to the equilibrium concentration of $O_2, C^e_{O_2}$, over the copper carrier, d_{PC} is the diameter of a single char particle, and $k_{G,C}$ is the coefficient of external mass transfer for transfer of O_2 to the surface of the char particle, m/s.

For coal particles, the average particle size is $d_{PC} = 1.3\times10^{-3}$ m, with a density of ρ_{coal} = 800 kg m^{-3}. The mass of a single coal particle is $m_{coal} = 9.20\times10^{-4}$ g per particle. At 900 °C, with fluidizing gas N_2 of 53.6 cm^3 s^{-1} (as measured at 293 K and 1 atm), U_{mf} =0.106 m s^{-1} and $\upsilon = 1.497\times10^{-4}$ m^2 s^{-1}, thus the Reynolds number for the particulate phase, $Re_P = U_{mf} d_{PC}/\upsilon = 0.92$, with the molecular diffusivity for O_2 in N_2 being $D_{O_2/N_2} = 2.2\times10^{-4} m^2 s^{-1}$, so that $Sc = \upsilon / D_{O_2/N_2} = 0.657$. The Sherwood number, *Sh,* was calculated from $Sh = 2\varepsilon_{mf} + 0.69(Re_p/\varepsilon_{mf})^{1/2} S_c^{1/3}$ = 2.0, where ε_{mf} is the voidage of the particulate phase, assumed to be 0.4. Therefore $k_{G,C} = ShD_{O_2/N_2}/d_{PC} = 0.341$ m s^{-1} and the rate of combustion of char, or rate of CO_2 production, is $r_{O_2} = k_{G,C}\pi d_{PC}{}^2 C_{O_2,e} = 2.8\times10^{-7}$ mol/particle/s = 3.05×10^{-4} mol g_{coal}^{-1} s^{-1}. The actual rate of combustion of bituminous coal char at 900 °C is 4.1×10^{-4} mol g_{coal}^{-1} s^{-1}, comparable to the theoretical external mass transfer, suggesting that the rate of combustion of the bituminous coal was controlled by external mass transfer (O_2 released from the copper oxides).

9. Kinetics analysis of combustion of coal in the presence of steam

At lower temperatures, the maximum theoretical rate of O_2 exchange between the bed and surface of a particle of char is given by the rate of CO_2 production:

$$r_{CO_2} = k_{G,C}\pi d_{PC}{}^2 C_{O_2,b} \text{ mol/g/s} \quad \text{(Equation S12)}$$

Here, $C_{O_2,b}$ is the bulk O_2 concentration in the bed, mol/m^3, not equal to the equilibrium concentration of $O_2, C^e_{O_2}$ in this study. k_{G,O_2} is the coefficient of external mass transfer of O_2 to the surface of the char particle, m/s. For the oxygen carrier, the average particle size is $d_P = 6\times10^{-4}$ m in the size range of 510-700 μm. The small amount of inert sand was not considered. At 850-985 °C, with fluidizing gas of steam and N_2 of 72.0 cm^3/s (as measured at 293 K and 1 atm), U_{mf} =0.11 m/s and $\upsilon = 1.497\times10^{-4}$ m^2/s, thus the Reynolds number for the particulate phase, $Re_P = U_{mf} d_{PC}/\upsilon = 0.92$, with the molecular diffusivity for O_2 in N_2 $D_{O_2/N_2} = 2.2\times10^{-4} m^2/s$, then $Sc = \upsilon / D_{O_2/N_2} = 0.657$. The Sherwood number, *Sh,* was calculated from $Sh = 2\varepsilon_{mf} + 0.69(Re_p/\varepsilon_{mf})^{1/2} S_c^{1/3}$ = 2.0, where ε_{mf} is the voidage of the particulate phase of the bed which can be assumed as 0.4. Therefore, the coefficient of

external mass transfer of O_2 to the oxygen carrier could be estimated as $k_{G,C} = ShD_{O_2/N_2} / d_{PC} = 0.341$ m/s. Because the concentration of O_2 was influenced by temperature, so the rate of combustion of char should be calculated individually. At 850 °C with steam as present, the fraction of O_2 in the bed is estimated as 0.74% based on the dry basis fraction of 1.0% and flow rate, so the maximum rate of CO_2 production is derived as $r_{CO_2} = k_{G,C} \pi d_{PC}{}^2 C_{O_2,b} = 1.45 \times 10^{-7}$ mol/particle/s = 1.58×10^{-4} mol/g coal/s, which is approximately the observed maximum rate of combustion of bituminous coal. The rate of combustion of lignite and lignite char is higher than the theoretical value. As presented above, the gasification of lignite and its char by steam or O_2 may contribute to the reaction of gas products with the oxygen carriers, therefore, the actual rate of combustion could be higher. However, at 900 and 950 °C, the estimated maximum rate of combustion r_{CO_2} = 6.1×10^{-4} mol/g/s is approximately the maximum measured rate of combustion of lignite and bituminous coal well. As for the lignite char the actual rate of combustion is higher than estimated, possibly owing to the temperature increase and associated higher $p_{O_2,b}$.

Table S2. ICP-MS analysis of coal ash, fresh and used oxygen carrier materials

Element	Mass	Error	Lignite coal ash	Bituminous Coal ash	Fresh	Used with lignite	Used with bituminous coal
	g mol^{-1}	mg kg^{-1}	mg kg^{-1}	mg kg^{-1}	mg kg^{-1}	mg kg^{-1}	mg kg^{-1}
Ag	106.9	0.0230	0.10	0.09	3.13	3.02	2.82
Al	27	0.2335	**>11025**	**>32688**	**>142315**	**>141726**	**>131474**
As	74.9	0.4035	5.59	20.87	0.00	0.00	0.82
B	11	0.1608	1404.96	154.44	0.00	2.77	4.08
Ba	136.9	0.0280	5148.89	1594.61	5.67	9.53	20.14
Be	9	0.1386	0.38	3.61	0.00	0.00	0.03
Bi	209	0.0880	0.00	0.37	0.00	0.00	0.05
Ca	43	2.0923	**>318649**	30496.69	1644.11	1791.78	1776.22
Cd	110.9	0.2046	0.65	2.83	0.00	0.31	0.00
Co	58.9	0.0182	6.02	14.11	0.00	0.01	0.32
Cr	51.9	0.0133	14.98	71.83	2.43	54.96	30.43
Cu	62.9	0.2121	624.77	232.72	**>352880**	**>354361**	**>338087**
Fe	56.9	0.1869	**>123507**	**>14863**	67.90	111.36	122.66
Ga	68.9	0.0169	192.05	75.67	11.61	11.55	11.26
Li	7	0.0259	86.64	51.84	3.15	7.03	11.47
Mg	24	0.0710	**>98567**	4406.77	49.34	82.26	76.50
Mn	54.9	0.0118	2391.25	365.05	0.81	2.10	2.61
Mo	97.9	0.0604	2.13	7.29	0.09	0.44	0.61
Ni	59.9	0.0914	20.60	40.88	6.60	7.26	7.27
Pb	208	0.0545	5.45	14.78	3.07	1.13	0.57
Pb	206	0.0454	5.49	15.74	3.17	1.11	0.57
Rb	84.9	0.0675	10.32	10.02	0.29	0.45	0.50
Se	81.9	0.6885	15.77	1.61	0.00	3.95	4.26
Sr	87.9	0.0093	3249.34	2139.99	18.53	22.56	36.80
Te	129.9	0.0267	5.20	0.06	0.00	0.00	0.02
Tl	205	0.0508	0.00	0.43	0.00	0.00	0.01
U	238.1	0.0737	0.58	5.47	0.00	0.00	0.00
V	50.9	0.0227	24.61	105.06	0.00	0.00	1.05
Zn	65.9	0.3729	6.03	67.83	4.10	15.71	3.84

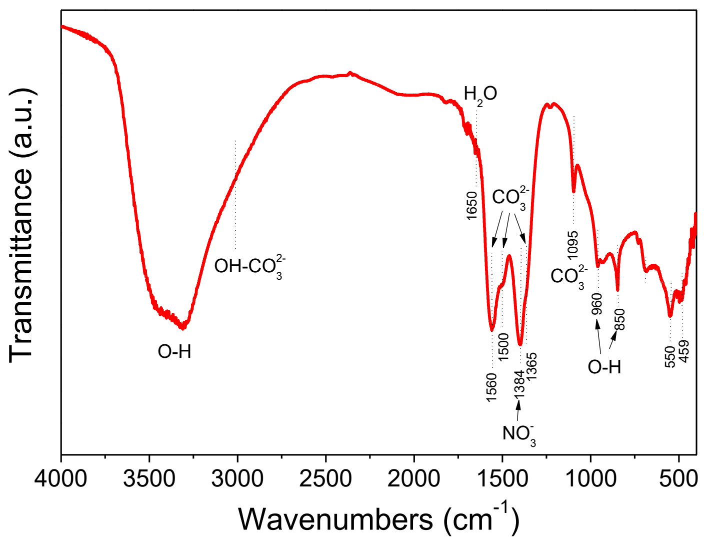

Figure S3. FTIR spectra of the Cu-Al LDH precursor.

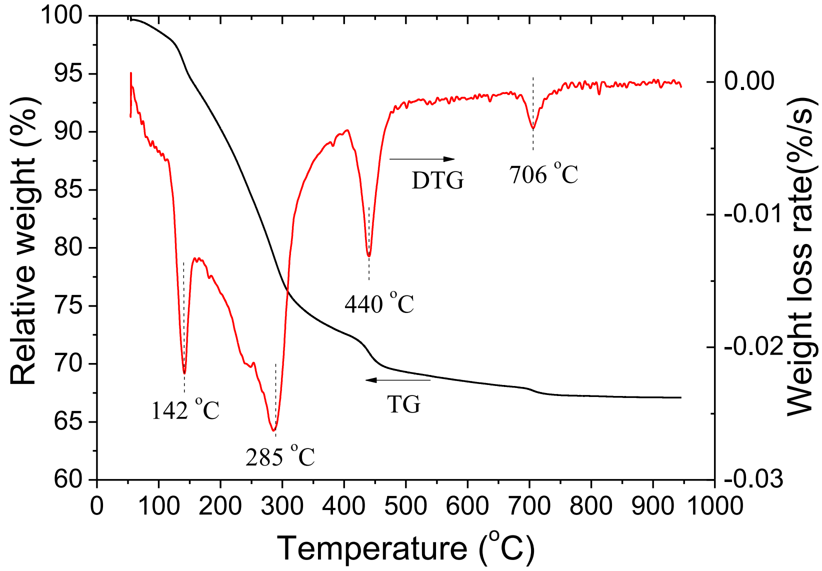

Figure S4. Thermogravimetric analysis of the Cu-Al LDH precursor. The sample was heated from 50 °C to 950 °C in air at a heating rate of 10 °C/min. The weight loss pattern agrees well with the literature.[5-6]

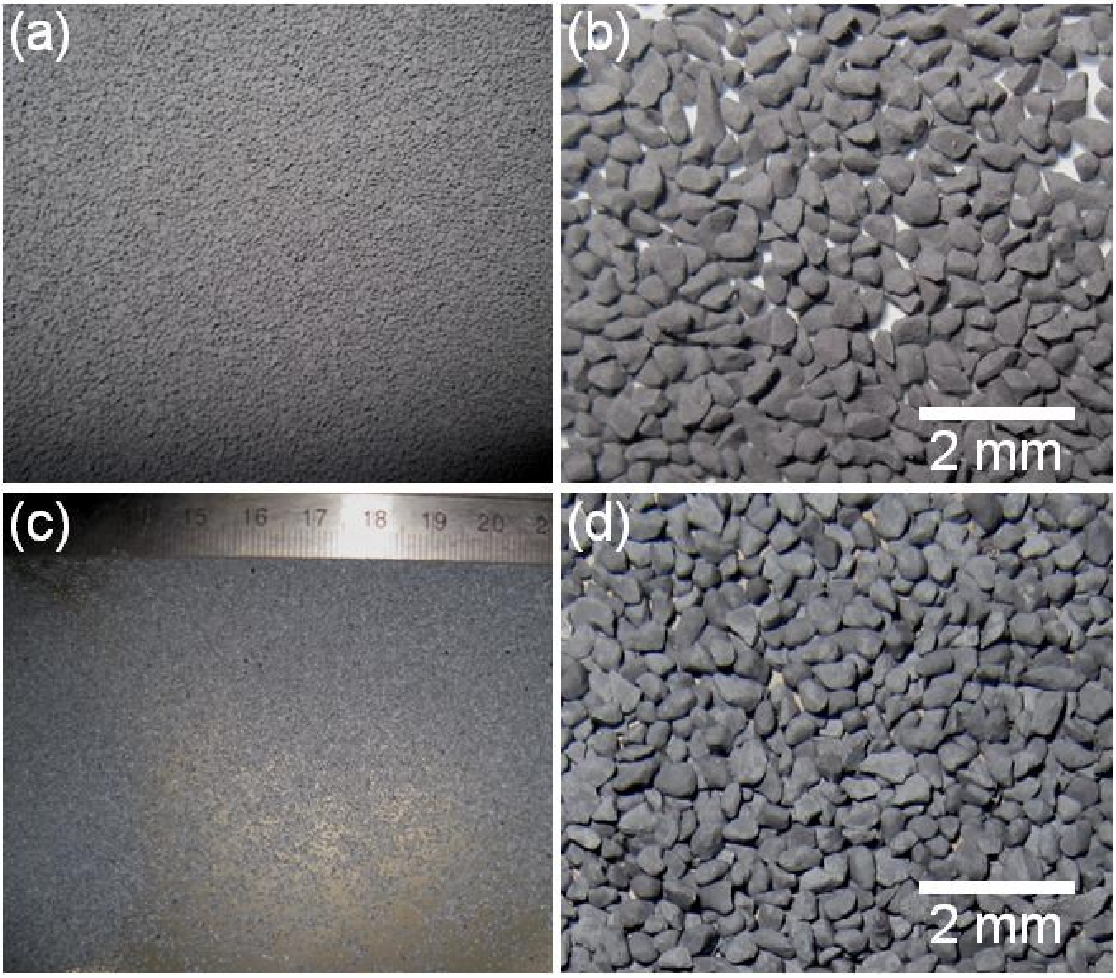

Figure S5. Photos of oxygen carrier particles. (a-b) fresh as calcined sample, and (c-d) used particles after 20 cycles of combustion of bituminous coal at 900 °C. Aagglomeration of particles did not occur. The size of fresh particles was in the range of 500-710 µm.

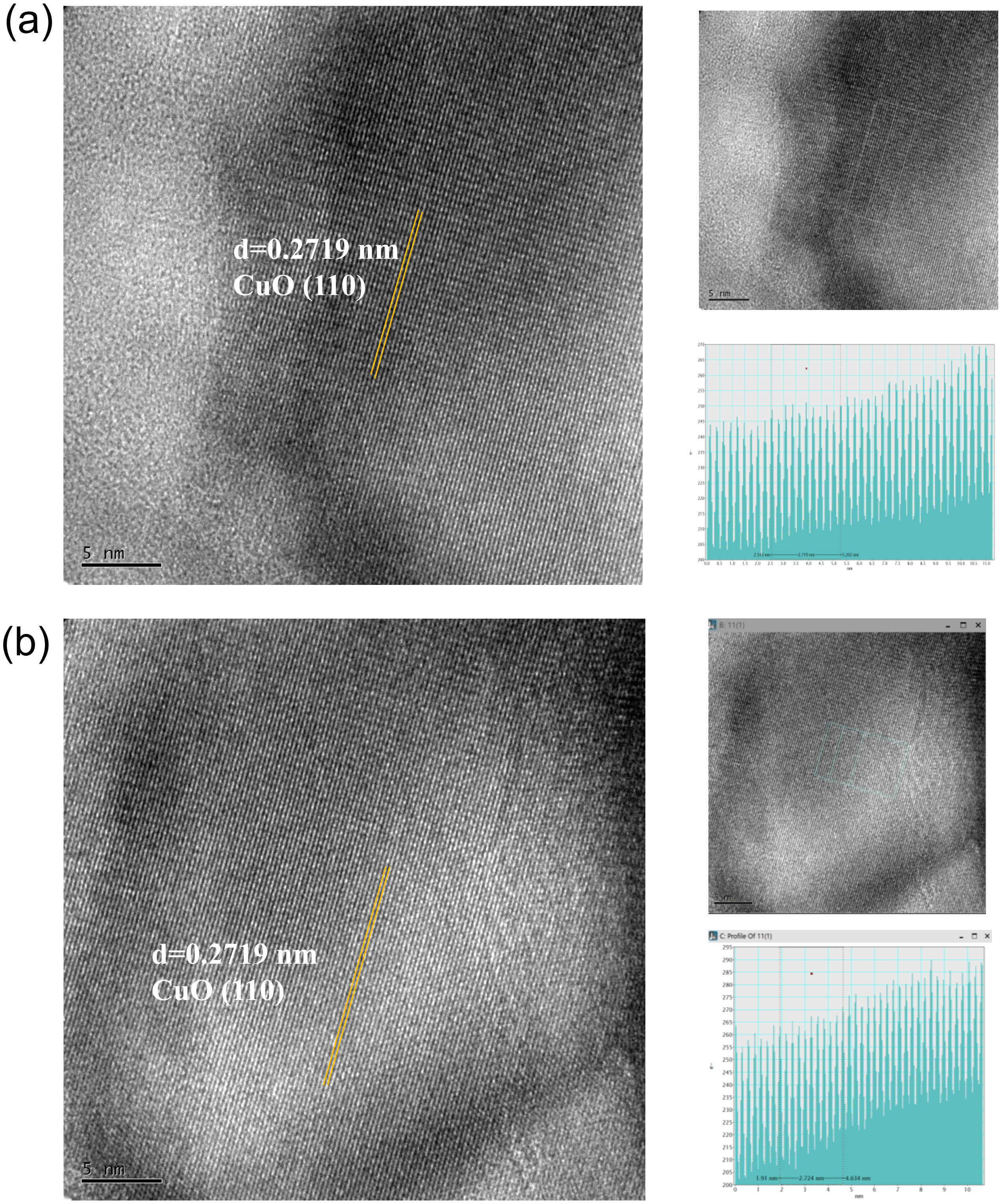

Figure S6. HR-TEM images and calculation of lattice fringe spacings. The two HRTEM images were taken at different positions of the same CuO nanoparticles with size of around 30-40 nm, (a) boundaries with amorphous domain included, (b) primarily crystalline domain.

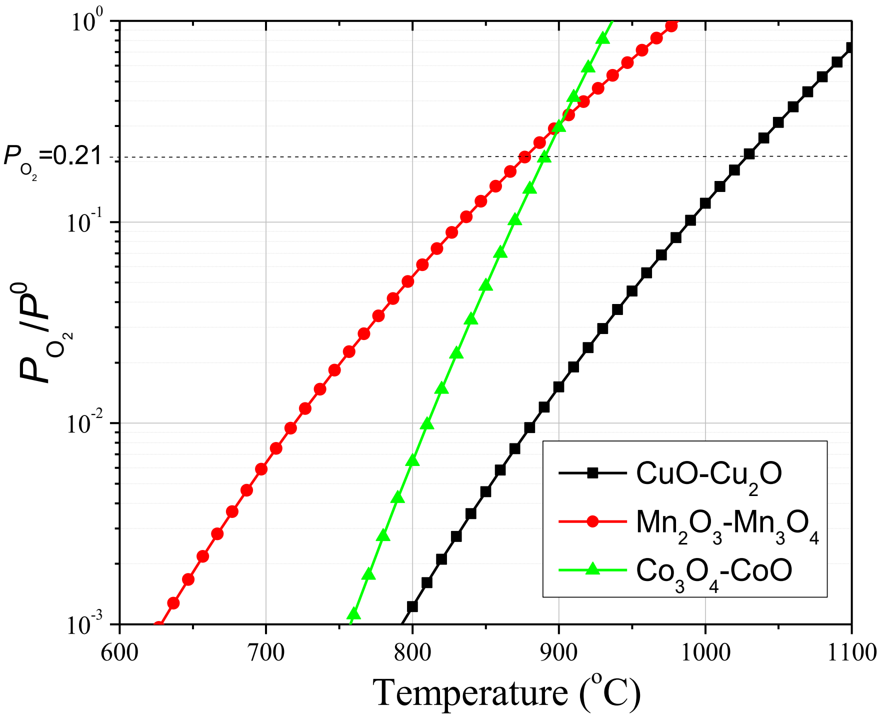

Figure S7. Thermodynamic analysis. Equilibrium partial pressure of O_2 versus temperature for representative metal oxides couples CuO/Cu_2O, Mn_2O_3/Mn_3O_4 and Co_3O_4/CoO. The thermodynamics data was taken from literature.[7]

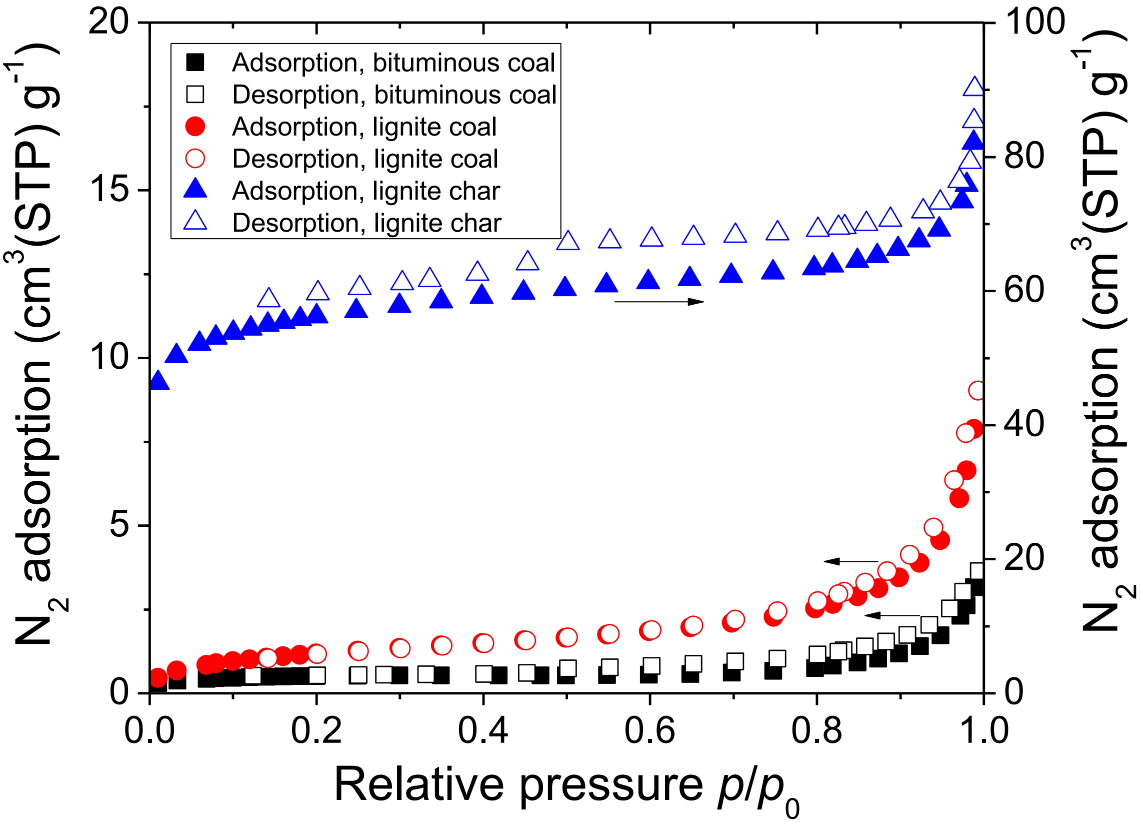

Figure S8. N_2 adsorption isotherms for lignite, lignite char, and bituminous coal.

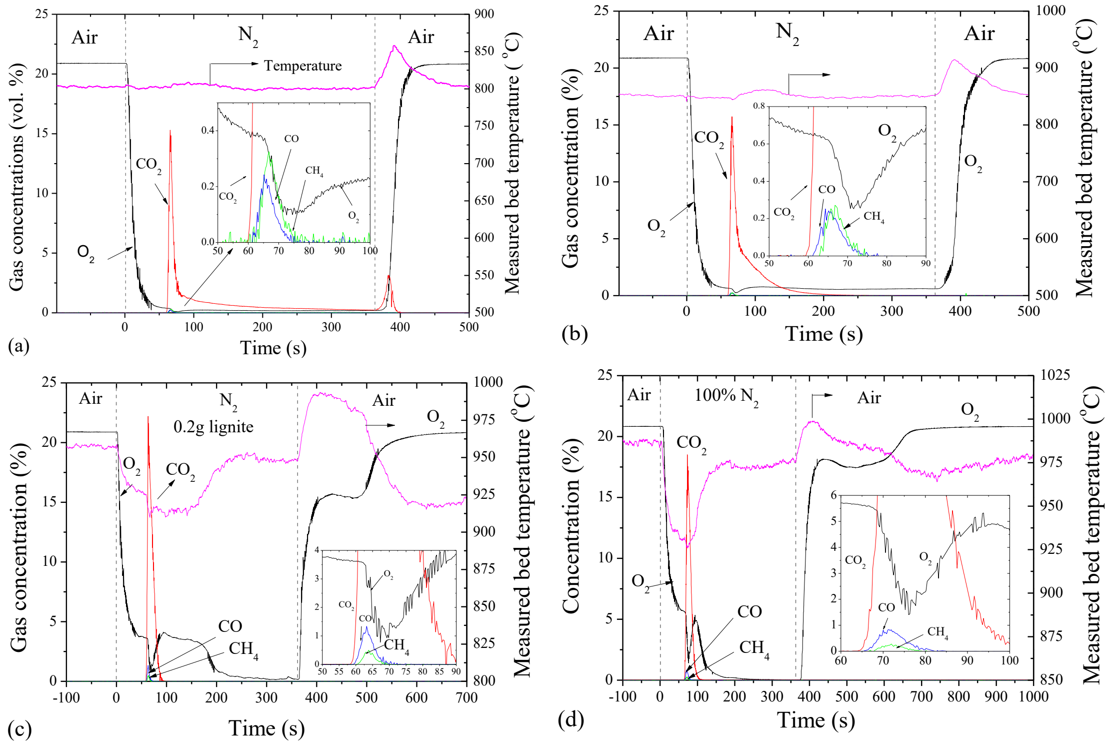

Figure S9. Profiles of combustion of lignite at different temperatures. Gas concentration and measured bed temperature profiles of combustion of 0.2 g of Hambach Lignite coal with 15 g of Cu-based oxygen carriers. (a) 800 °C, (b) 850 °C, (c) 950 °C, and (d) 985 °C. The insets show the detailed profile of gas concentrations during the initial stage of combustion of coal. For insets, ordinate has units of vol%, abscissa is in s.

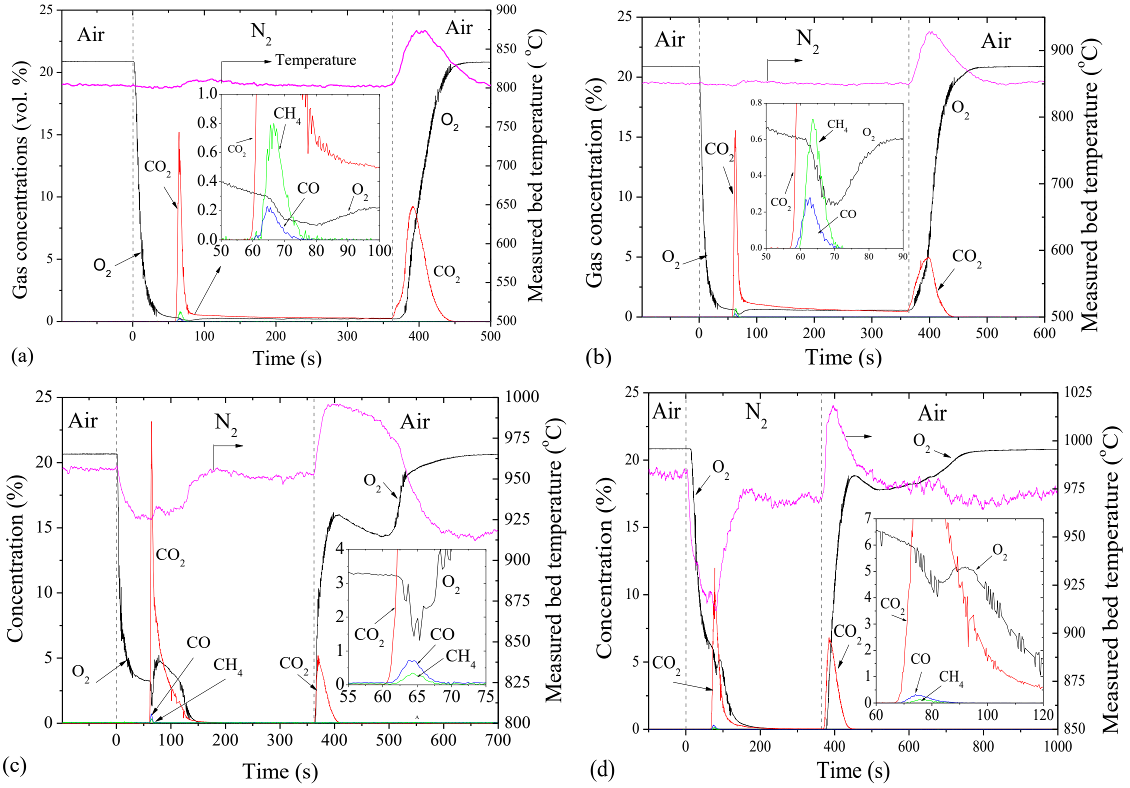

Figure S10. Profiles of combustion of bituminous coal at different temperatures. Gas concentration and measured bed temperature profiles of combustion of 0.2 g of Taldinskaya bituminous coal with 15 g of Cu-based oxygen carriers. (a) 800 °C, (b) 850 °C, (c) 950 °C, and (d) 985 °C. The insets show the detailed profile of gas concentrations during the initial stage of combustion of coal. For inset, ordinate has units of vol%, abscissa is in s. At 985 °C, the rate of O_2 release is too fast that the O_2 partial pressure drops quickly after switching the inlet gas to N_2, therefore a large fraction of the O_2 will be lost before the bed enters the "fuel reactor" environment, thus reaction at this temperature was not considered further.

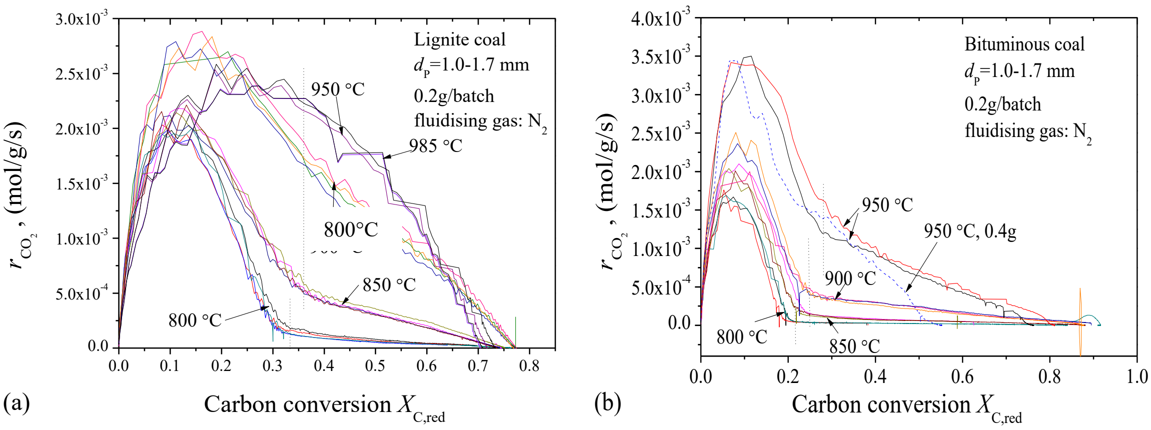

Figure S11. The rate of CO_2 production (r_{CO_2}) as a function of carbon conversion ($X_{OC,red}$). (a) lignite and (b) bituminous coal. The vertical dashed lines indicate the transition point of combustion of volatiles to char. The rate of CO_2 at this point was assumed as the maximum rate of combustion of char, as shown in the Figure 4f in the main text.

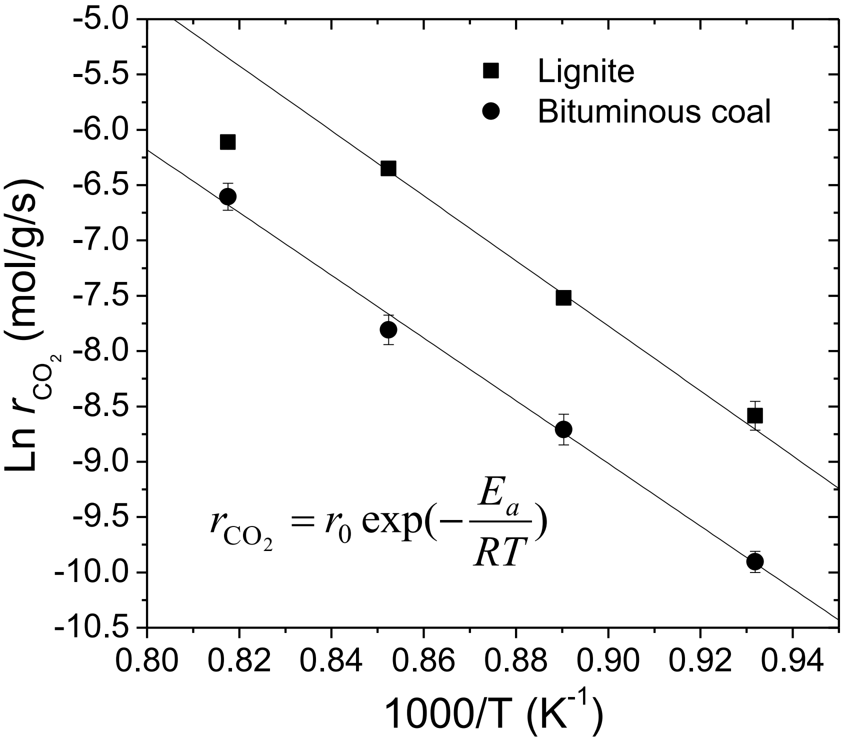

Figure S12. Activation energy. Plot of rate of CO_2 production $\mathrm{Ln}r_{CO_2}$ as a function of set-point temperature 1000/*T* during combustion of lignite and bituminous coal in the presence of Cu-based oxygen carriers in the fluidized bed. The apparent activation energy of char combustion was calculated to be 233.6 ± 12.2 and 235.4 ± 11.3 kJ mol^{-1} for the combustion of lignite and bituminous coal respectively. Note the reaction enthalpy (ΔH^0) of decomposition of CuO to Cu_2O is 260 kJ mol^{-1} within 800-950 °C at atmospheric pressure.

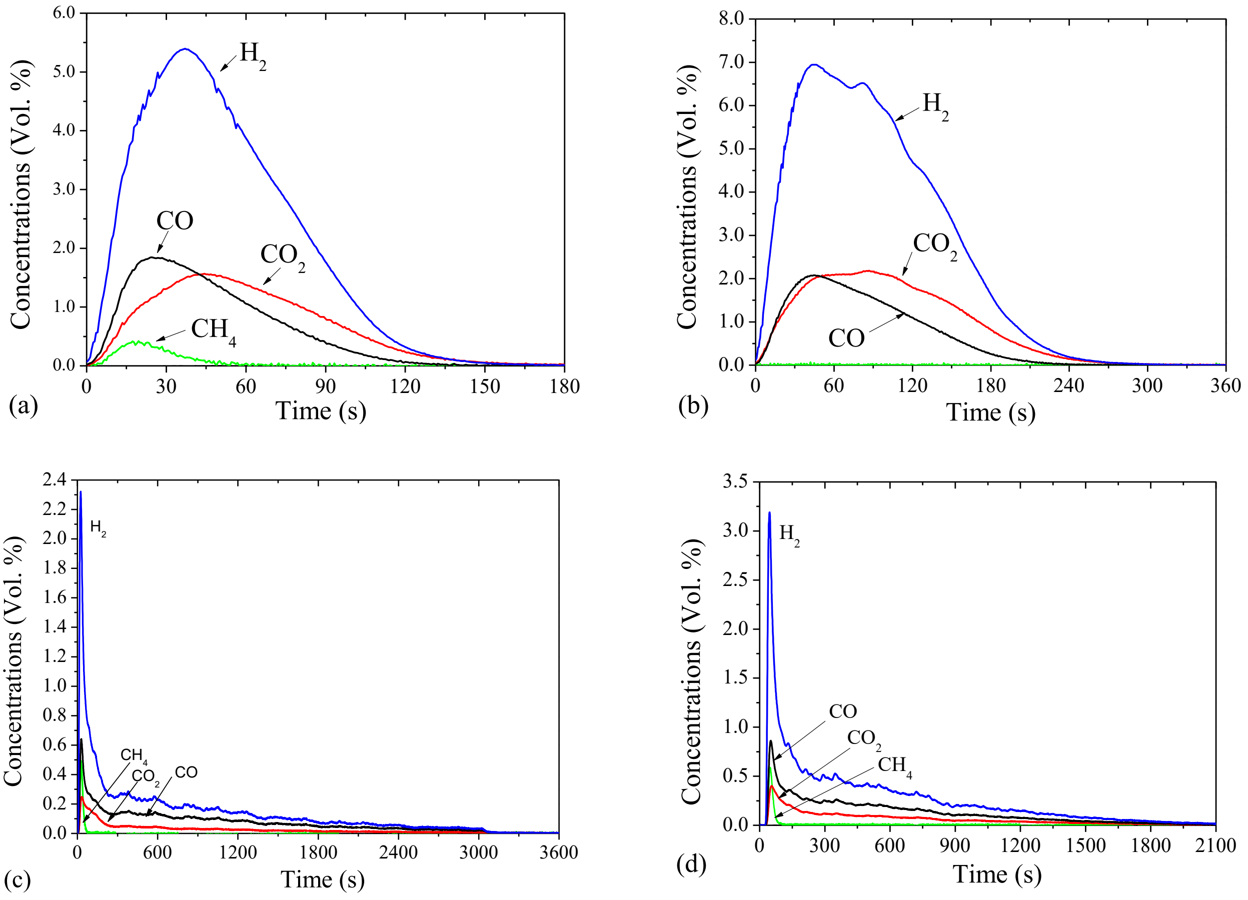

Figure S13. Gasification profile of (a) Hambach lignite at 900 °C. (b) Hambach lignite char at 900 °C, (c) Taldinskaya bituminous coal at 900 °C and (d) Taldinskaya bituminous coal at 950 °C in the presence of alumina sand fluidized by 25.6 vol% H_2O balanced by N_2. The flow rate of H_2O was 49mL/h with the balance of 53.6 cm^3/s N_2, corresponding to 25.6% H_2O/N_2 and 72.0 cm^3/s. The oxidation gas was air, flow rate of 50.9 cm^3/s. All gas flow rates are normalized to 293 K and 1 bar. The combustion of unburned char is not included.

Figure S13 shows the dry basis gas concentration profiles of gasification of three different solid fuels at typical temperatures as indicated. For all fuels, the gasification products were mainly H_2, CO, and CO_2. The initial release of CH_4 as observed during gasification of lignite and bituminous coal was mainly from volatiles and pyrolysis gases, which was not observed in gasification of lignite char. The higher concentration of H_2 was much higher than that of CO or CO_2 which was due to the influence of water gas shift reaction (WGSR, $CO+H_2O=H_2+CO_2$). Under higher steam concentration condition, the WGSR would be enhanced with the H_2/CO_2 ratio near the thermodynamics theoretical ratio of 2. Note that in all the experiments performed in this work, the off gas was sampled just above the bed, thus give a much more accurate product gas composition for evaluation of the intrinsic gasification reaction with less interference of homogeneous reaction including WGSR and methane reforming.
As can be compared from the gasification profiles, the reactivity of lignite is approximate to its derived char and much higher than that of bituminous coal, which takes nearly 60 min to reach around 65mol% carbon conversion at 900 °C with the rest char accumulated in the bed. With the temperature increased, the char gasification was much faster and reached 90mol% carbon conversion after 30min. Detailed quantitative analysis on the gasification rate and carbon conversion will be compared with the data in CLC experiments.

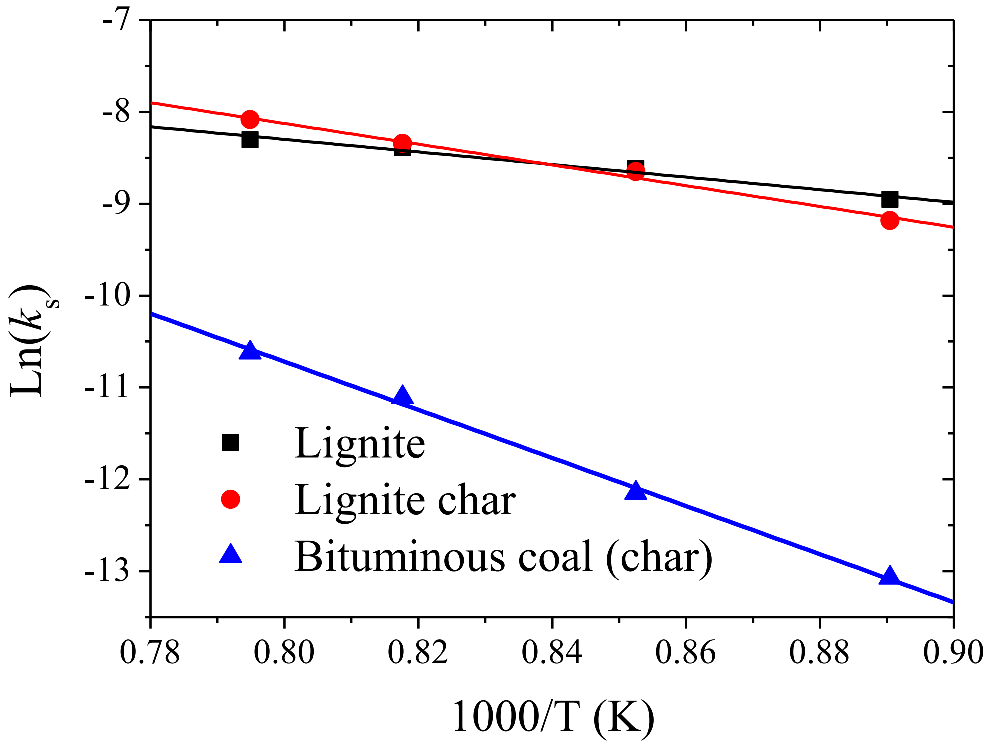

Figure S14. Arrhenius plots. Plots of the reaction rate constant ln(k_s) as a function of set-point temperature 1000/T during the gasification of coal over inert bed.

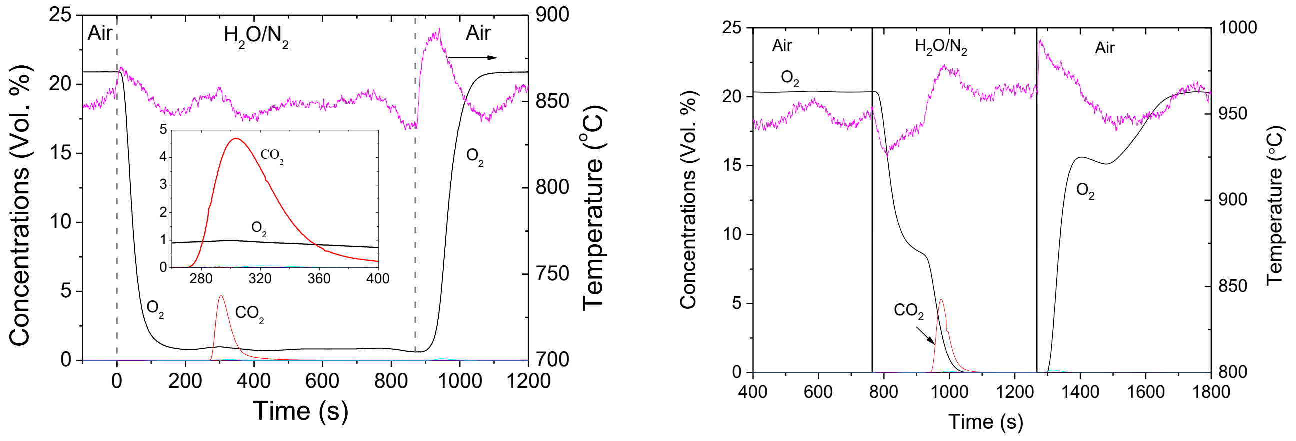

Figure S15. Combustion profiles in the presence of oxygen carriers. Concentration profile of combustion of 0.2 g Hambach lignite coal with 20 g coprecipitated 60 wt% CuO/Al_2O_3 fluidized by 25.6 vol% H_2O balanced by N_2 at 850 °C (left) and 950 °C (right). The flow rate of H_2O was 49mL/h with the balance of 53.6 cm^3/s N_2, corresponding to 25.6% H_2O/N_2 and 72.0 cm^3/s. The oxidation gas was air, flow rate of 50.9 cm^3/s. For inset, ordinate has units of vol%, abscissa is in s. All gas flow rates are normalized to 293 K and 1 bar.

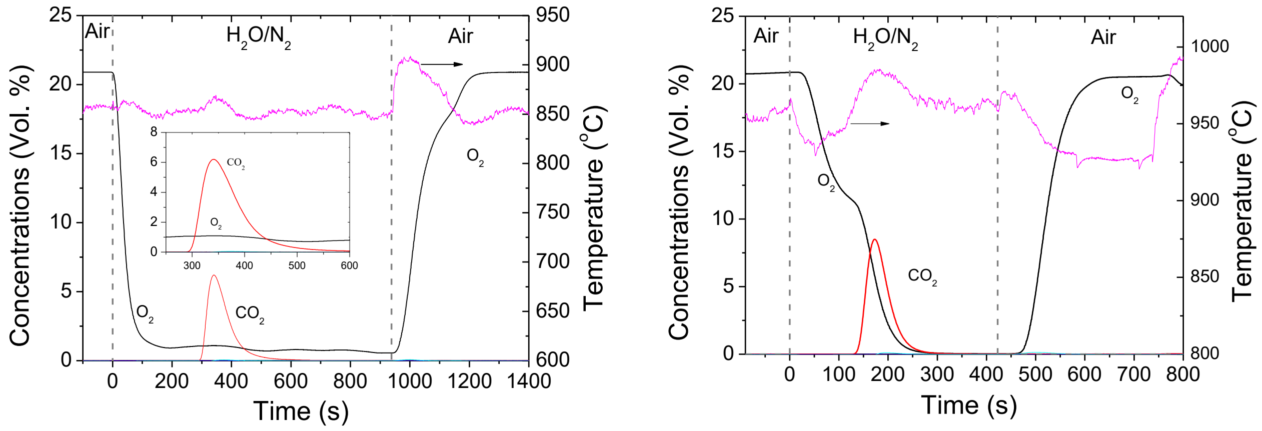

Figure S16. Combustion profiles in the presence of oxygen carriers. Concentration profile of combustion of 0.2 g Hambach Lignite char with 20 g coprecipitated 60 wt% CuO/Al_2O_3 fluidized by 25.6 vol% H_2O balanced by N_2 at 900 °C (left) and 950°C (right). The flow rate of H_2O was 49mL/h with the balance of 53.6 cm^3/s N_2, corresponding to 25.6% H_2O/N_2 and 72.0 cm^3/s. The oxidation gas was air, flow rate of 50.9 cm^3/s. For inset, ordinate has units of vol%, abscissa is in s. All gas flow rates normalized to 293 K and 1 bar.

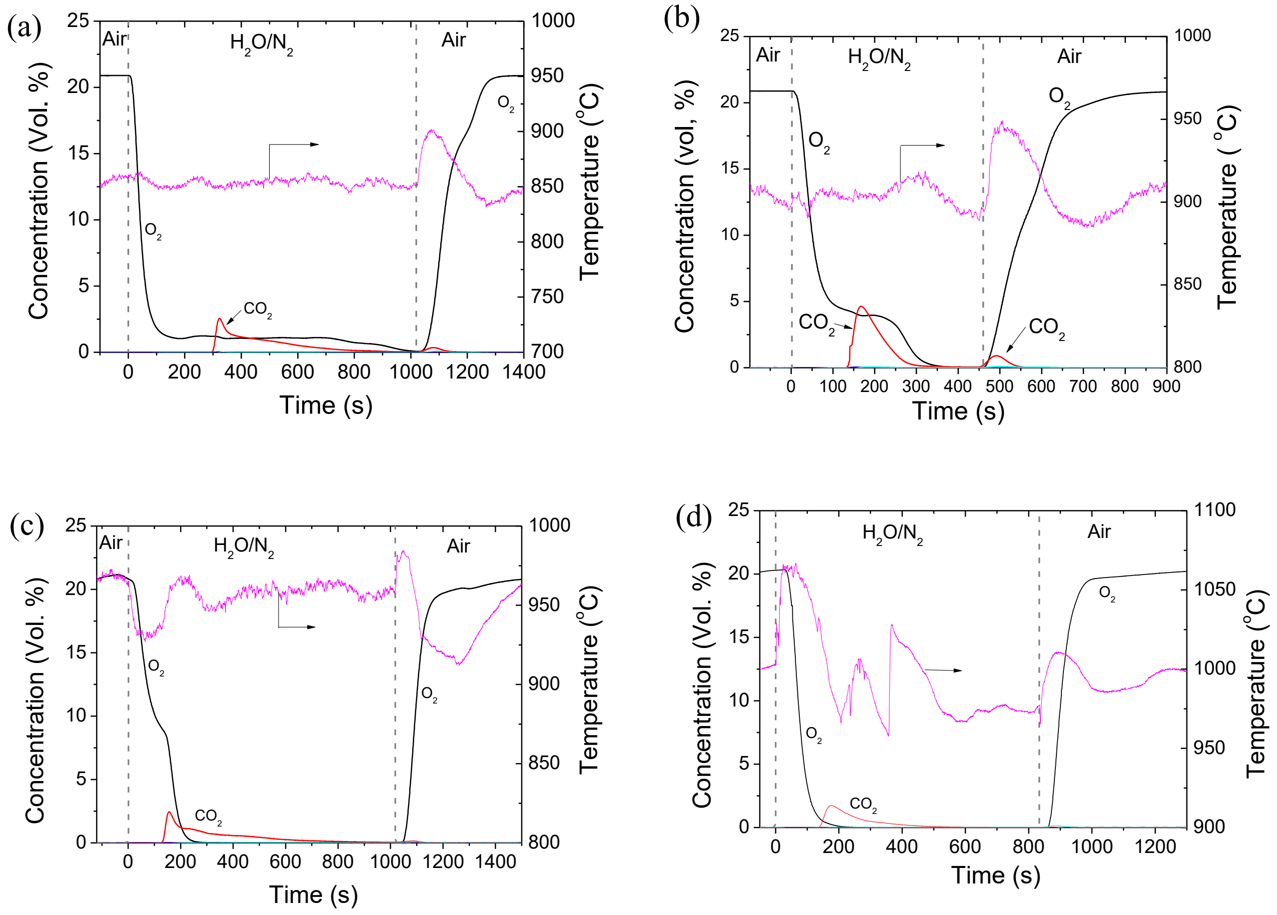

Figure S17. Combustion profiles in the presence of oxygen carriers. Concentration profiles of combustion of a batch of Taldinskaya bituminous coal with 20 g 60 wt% CuO/Al_2O_3 fluidized by 25.6 vol% H_2O balanced by N_2 at (a) 850 °C, (b) 900 °C, (c) 950 °C, and (d) 985 °C. The flow rate of H_2O was 49mL/h with the balance of 53.6 cm^3/s N_2, corresponding to 25.6% H_2O/N_2 and 72.0 cm^3/s. The oxidation gas was air, flow rate of 50.9 cm^3/s. All gas flow rates are normalized to 293 K and 1 bar. Note that the mass of coal was 0.2 g over the temperature of 850-950 °C, whilst 0.1 g was used to avoid defluidization.

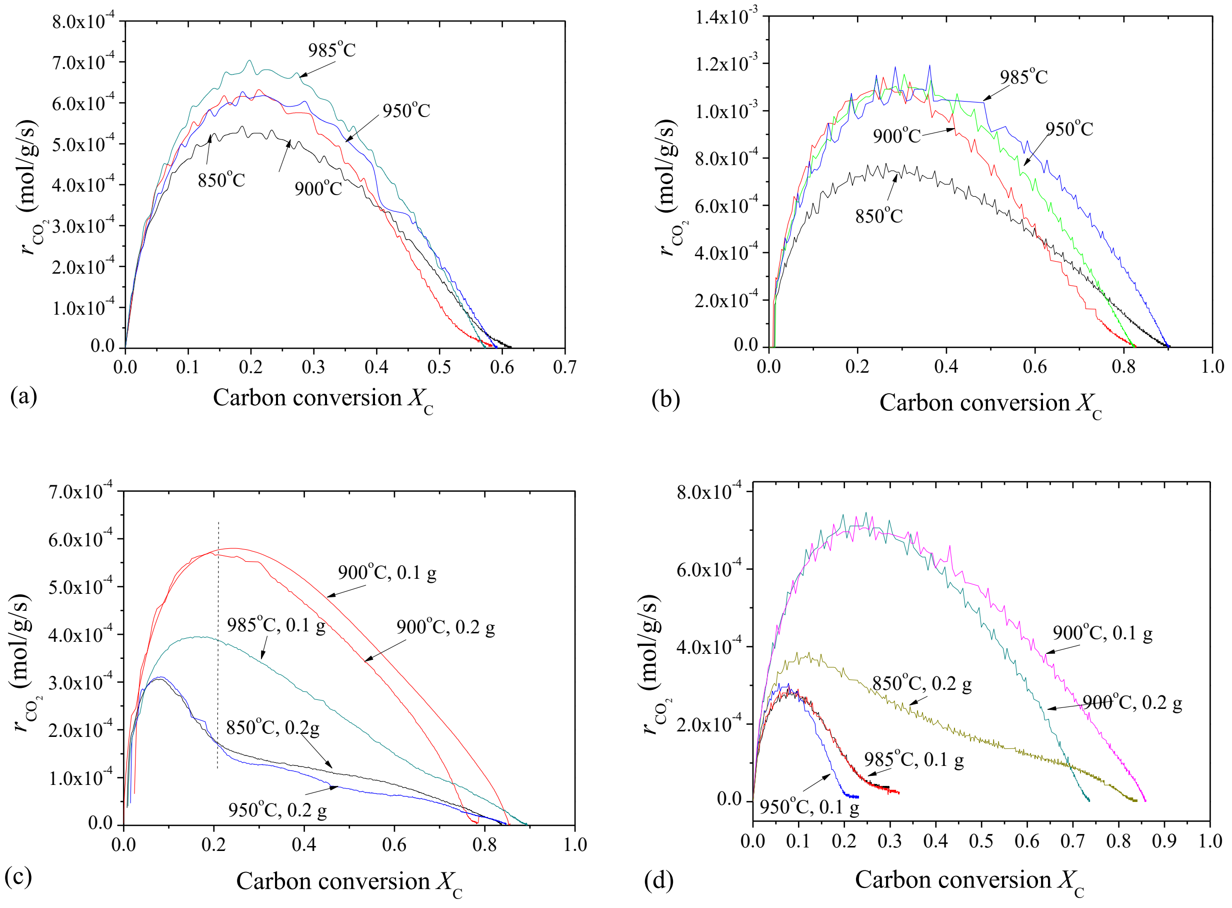

Fig. S18. Rate of combustion of coal expressed as rate of production of CO_2 as a function of carbon conversion (X_C). (a) combustion of lignite in the presence of CuO/Al_2O_3 fluidised by 25.6 vol% H_2O in N_2, (b) combustion of lignite char in the presence of CuO/Al_2O_3 fluidised by 25.6 vol% H_2O in N_2, (c) combustion of bituminous coal in the presence of CuO/Al_2O_3 fluidised by 25.6 vol% H_2O in N_2, and (d) combustion of bituminous coal in the presence of CuO/Al_2O_3 fluidised by pure N_2. It should be noted that the low combustion rates of bituminous coal at high temperature of 950°C and 985°C were mainly due to the rapid O_2 depletion in the bed and slow combustion during iG-CLC period.

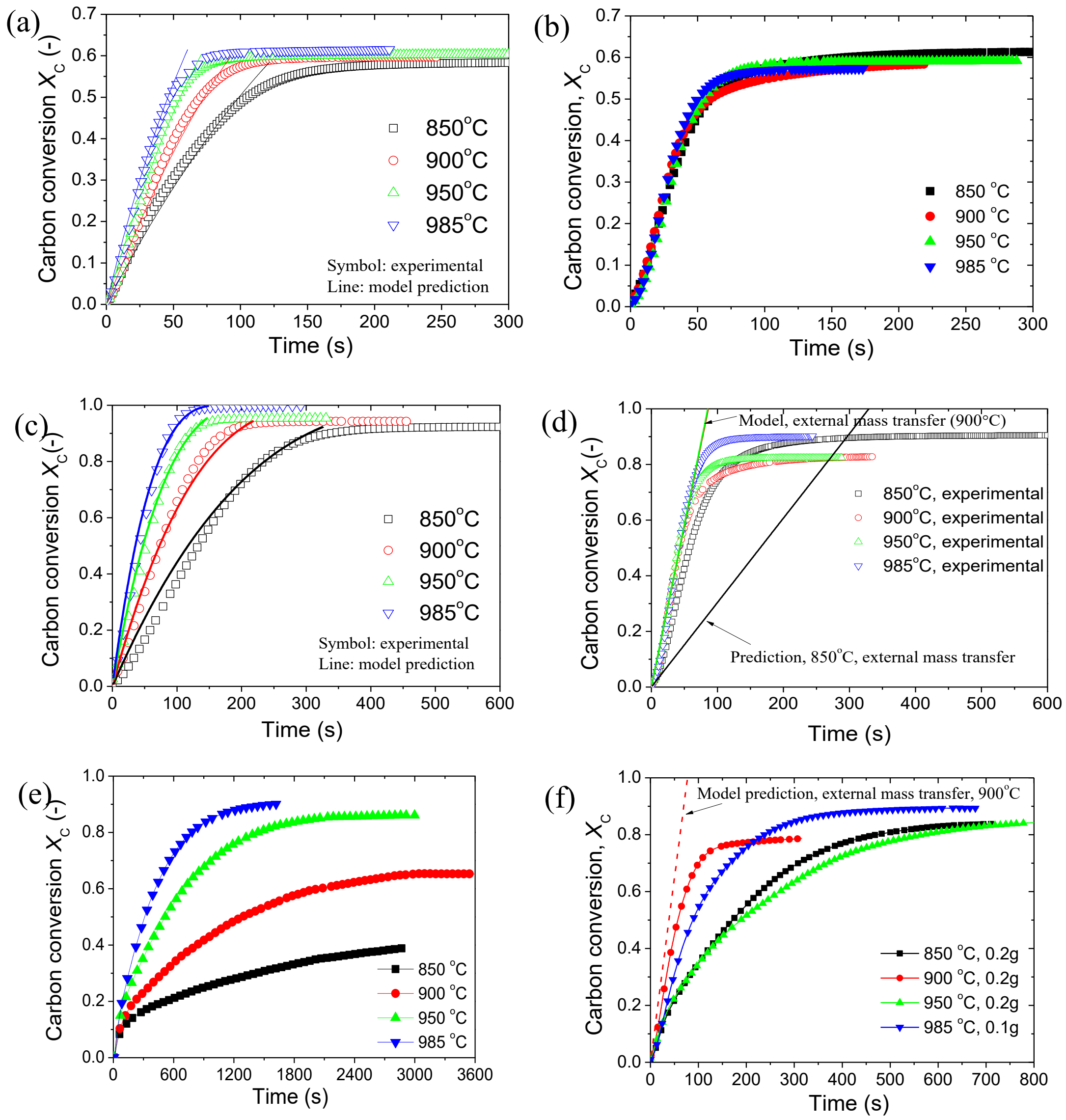

Figure S19. Carbon conversion of coal in gasification and iG-CLC. (a) Gasification of Hambach Lignite coal in the presence of inert alumina sand bed and (b) combustion of lignite in the presence of oxygen carrier. (c) Gasification of lignite coal char; (d) Combustion of lignite coal char in the presence of oxygen carrier; (e) Gasification of bituminous coal; and (f) combustion of bituminous coal in the presence of oxygen carrier. In combustion, 20 g Cu oxygen carriers were fluidized by 25.6 vol% H_2O balanced by N_2 within temperature of 850-985 °C The flow rate of H_2O was 49 mL/h with the balance of 53.6 cm^3/s N_2, corresponding to 25.6% H_2O/N_2 and 72.0 cm^3/s. The oxidation gas was air, flow rate of 50.9 cm^3/s. All gas flow rates are normalized to 293 K and 1 bar. The mass of coal in most experiments was 0.2 g, except 0.1 g of bituminous coal at 985 °C.

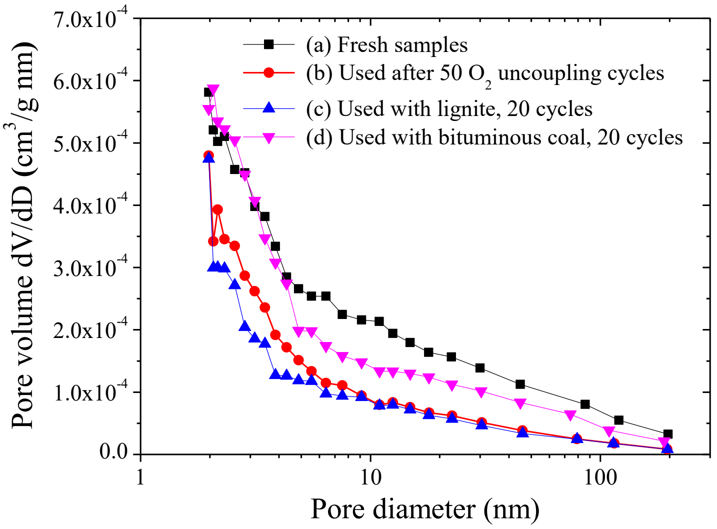

Figure S20. Pore size distribution of the fresh and used Cu-based oxygen carriers, calculated from the desorption branch of isotherms using the BJH model. (a) fresh particles (b) used particles after 50 cycles of decomposition/oxidation cycles, (c) used particles with lignite at 900 °C, (d) used particles with bituminous coal at 900 °C.

Table S3. Pore size properties of fresh and used oxygen carriers

Sample	S_{BET}	V_P	V_m	d_w	d_P
	m^2/g	mm^3/g	mm^3/g	nm	nm
Fresh (AlCu60-950-5)	5.2	20.6	0.631	9.4	21.6
Used with lignite	2.6	6.85	0.330	6.8	16.7
Used with bituminous coal	3.5	14.3	0.158	9.0	18.4
Used without fuel	2.2	7.38	0.072	8.2	15.3

S_{BET} :BET surface area; V_P : total pore volume; V_m : micropore volume; d_w: the average pore width; d_P : average pore diameter.

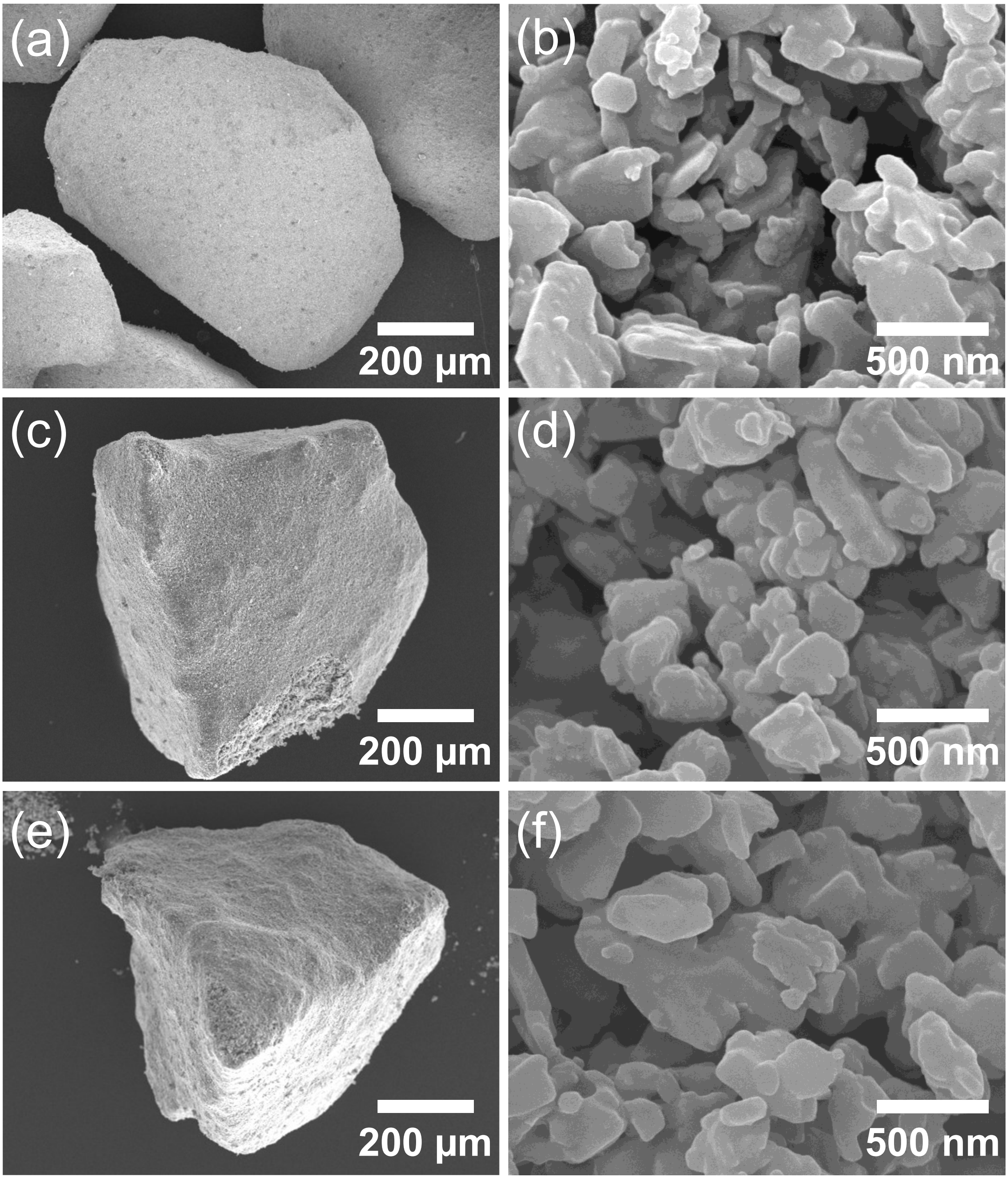

Figure S21. SEM images of cycled oxygen carrier particles. (a-b) oxygen carriers after 20 cycles of oxygen release and storage at 900 °C without fuels, (c-d) used oxygen carriers after 20 cycles of combustion of lignite at 900 °C, (e-f) used oxygen carriers after 20 cycles of combustion of bituminous coal at 900 °C.

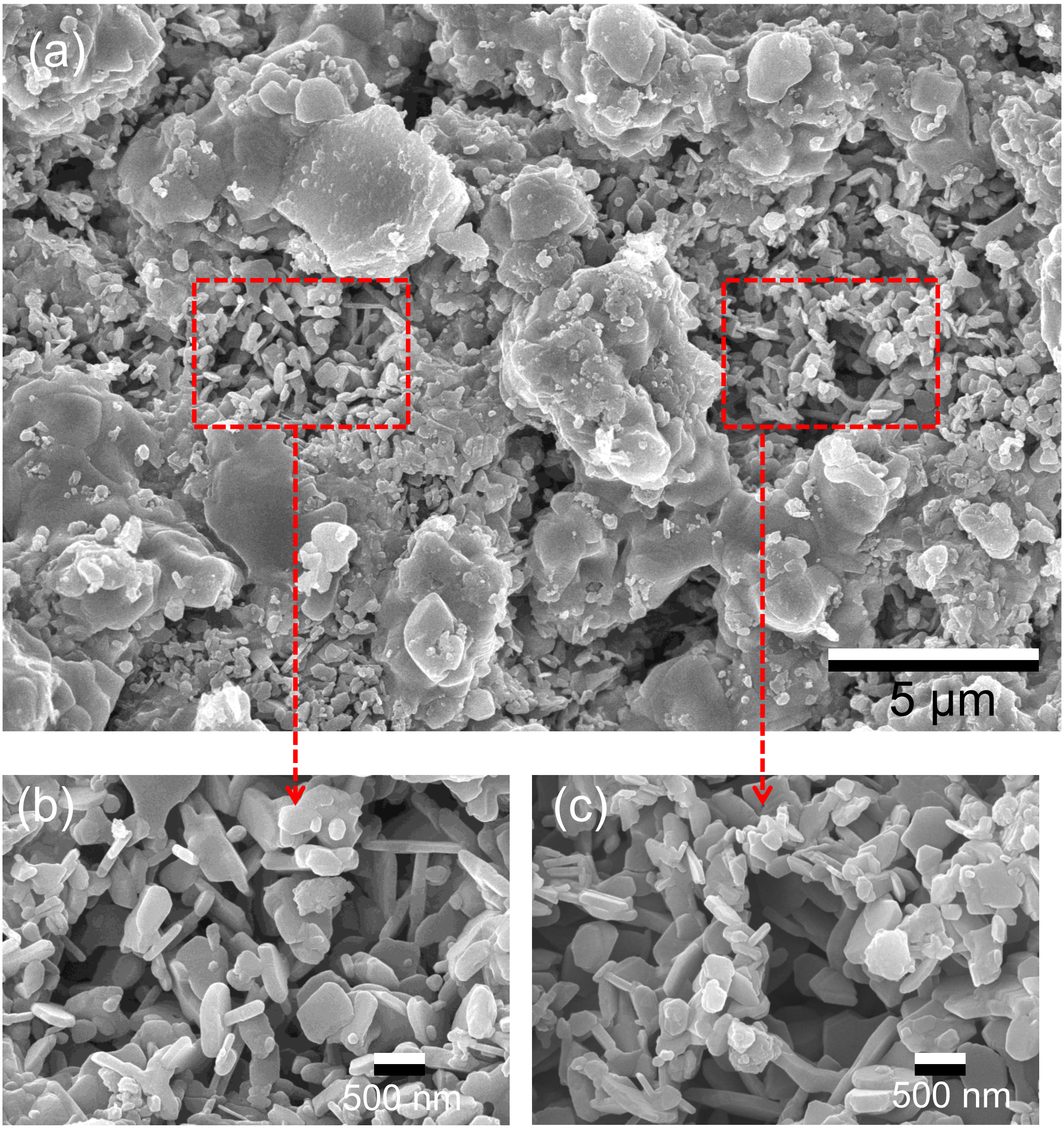

Figure S22. SEM images of cycled Cu-based oxygen carrier particles. The fresh calcined oxygen carriers were exposed to 20 redox cycles (phase changes between CuO-Cu_2O-Cu) of reduction with gaseous fuel (CO) and oxidation with air as the oxidant at a set-point temperature of 950 °C.

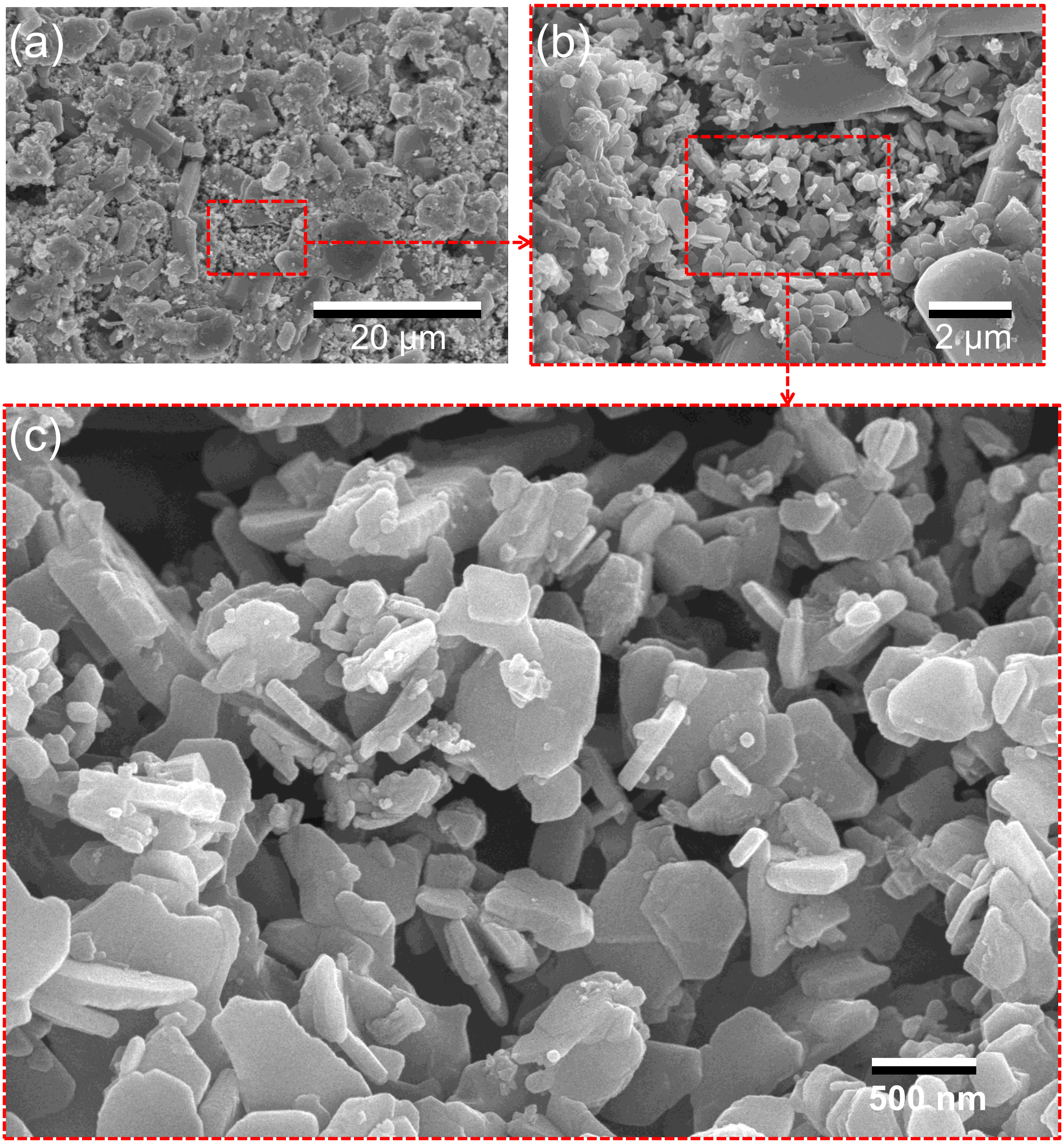

Figure S23. SEM images of cycled Cu-based oxygen carrier particles. The top-right inset in (a) shows the STEM image. The material was recovered from 20 cycles of O_2 release and storage for coal combustion (CuO-Cu_2O) and further exposed to 20 redox cycles (phase changes between CuO-Cu_2O-Cu) with gaseous fuel (CO) as the reductant and air as oxidant at set-point temperature of 950 °C.

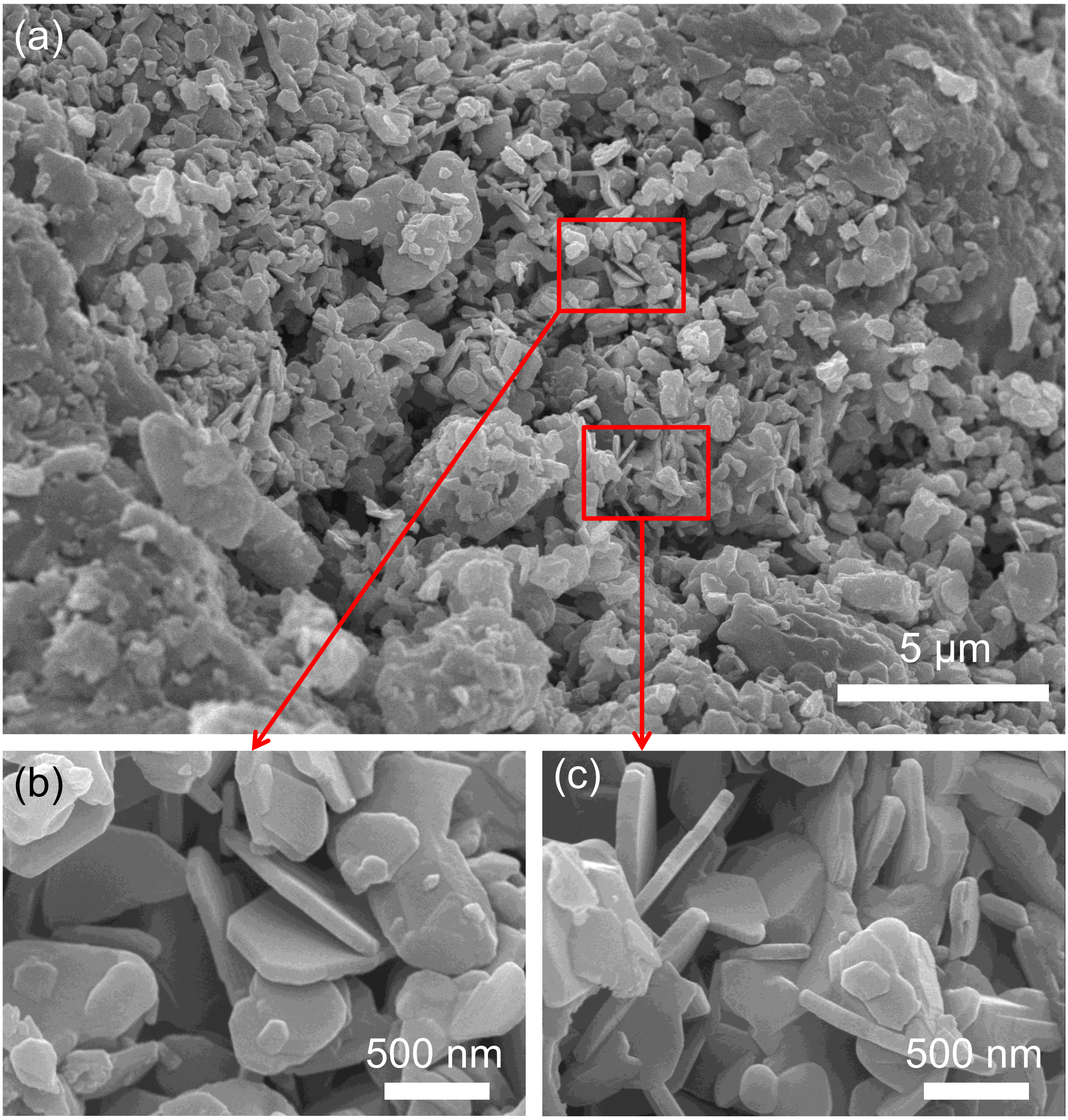

Figure S24. SEM images of cycled Cu-based oxygen carrier particles. (a-c) The material was recovered from iG-CLC cycles for coal combustion over 800-985 °C in the presence of steam.

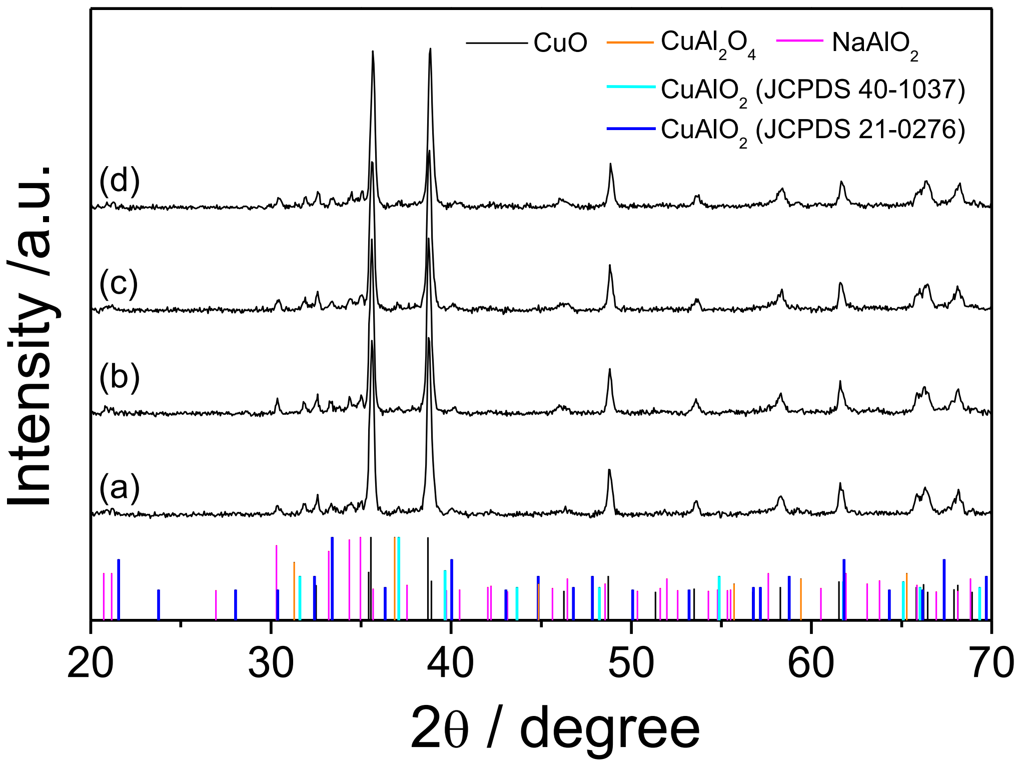

Figure S25. XRD patterns of fresh and cycled Cu-based mixed metal oxides (oxidized state). (a) as calcined, (b) after 20 cycles with lignite at 900 °C, (c) after 20 cycles with bituminous coal at 900 °C, (d) after 20 cycles of O_2 release and storage at 900 °C without solid fuels. Major phase is crystalline CuO (JCPDS 48-1548), with very weak peaks corresponding to $CuAl_2O_4$ (JCPDS 33-0448), $CuAlO_2$ (JCPDS 40-1037), $CuAlO_2$ (JCPDS 21-0276), and $NaAlO_2$ (JCPDS 33-1200).

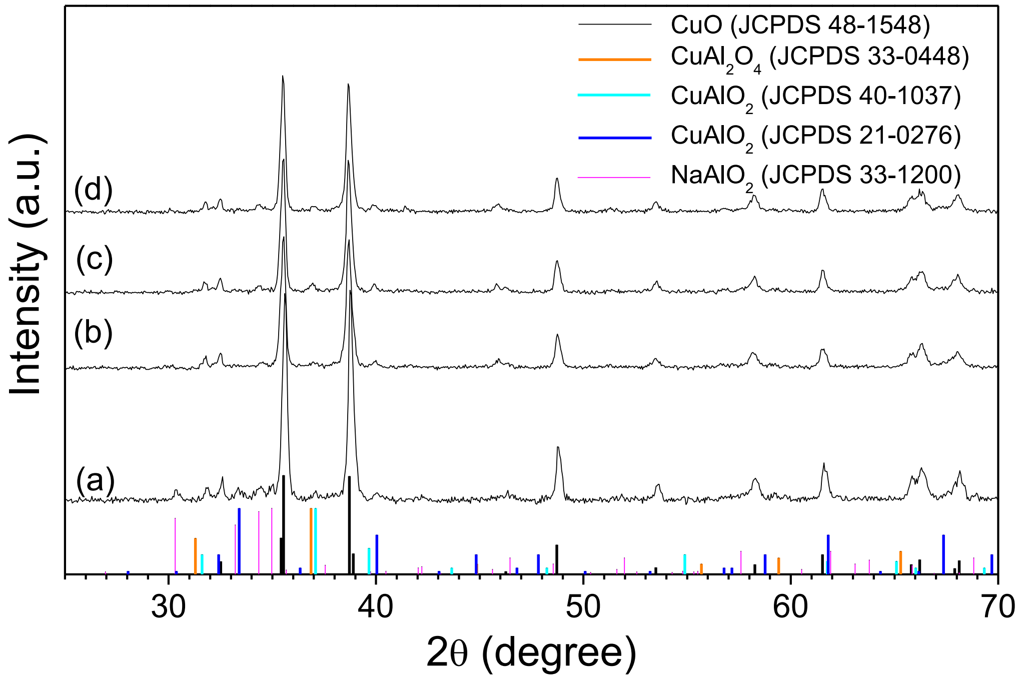

Figure S26. XRD patterns of fresh and cycled mixed metal oxides. (a) Fresh as calcined, (b) after 20 cycles of reduction and oxidation at 950 °C, (c) sample after combustion of lignite for 20 cycles, further exposure to gaseous fuels for 20 cycles, (d) sample after combustion of bituminous coal, further exposure to gaseous fuels for 20 cycles.

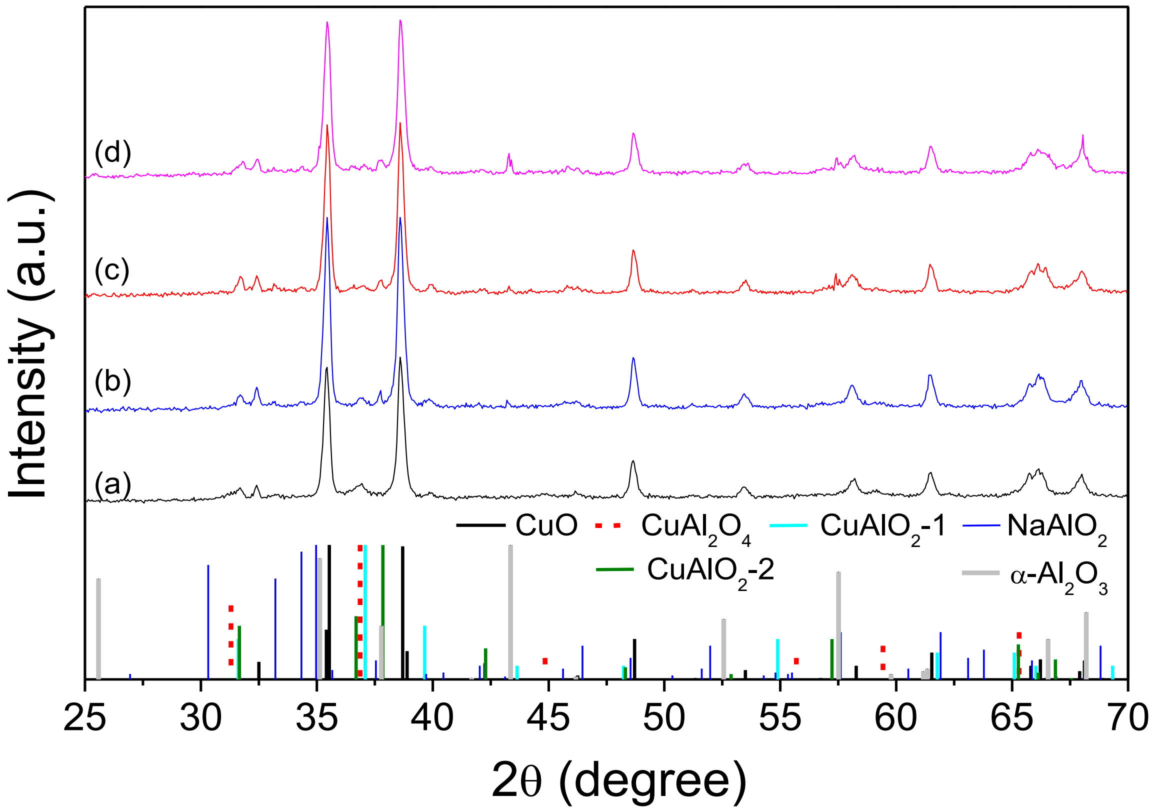

Figure S27. XRD patterns of fresh and used oxygen carriers. (a) fresh as-calcined oxygen carriers, (b) fresh oxygen carriers exposed to 20 redox cycles with CO as fuel gas, (c) used samples with solid fuels over 850-985 °C, and (d) used samples further exposed to 20 redox cycles at 950 °C. Reference patterns: CuAl2O4 (PDF-40-1037); $NaAlO_2$ (PDF-33-1200); tenorite CuO (PDF-48-1548); corundum, $\alpha-Al_2O_3$ (ICDD-10-0173); $CuAlO_2$ (PDF-35-1401);

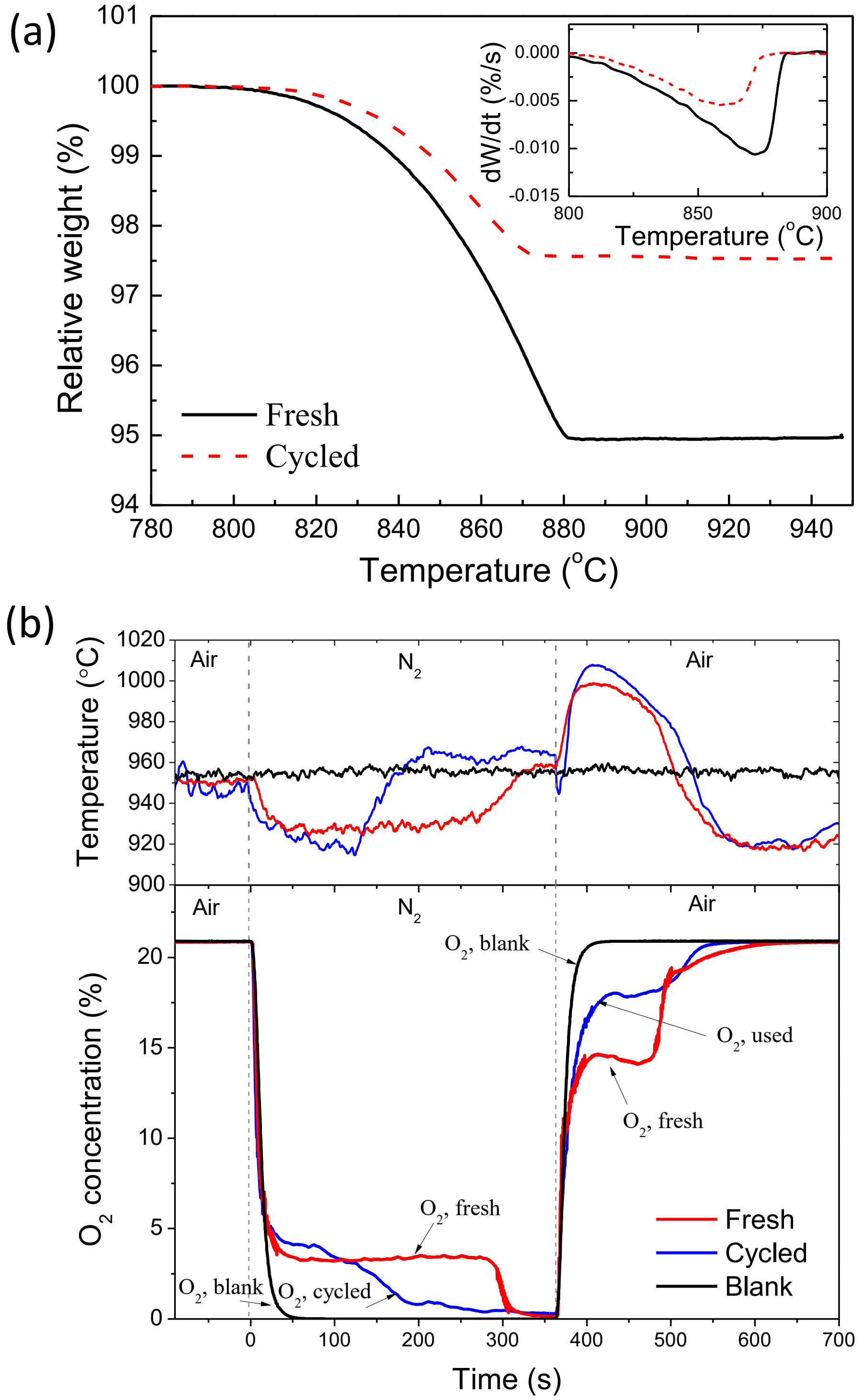

Figure S28. O_2 release and storage in fresh and cycled oxygen carrier. (a) Temperature programmed decomposition of the fresh as-calcined Cu-based oxygen carriers and cycled samples after solid fuels experiments (iG-CLC at 850-985 °C in steam). (b) The concentration and measured bed temperature profiles of cyclic O_2 release and uptake of the fresh and used oxygen carriers with solid fuels. The dash line indicates the switch of inlet gas between N_2 and air. The blank curve of O_2 fraction in the off gases with inert Al_2O_3 bed is also included.

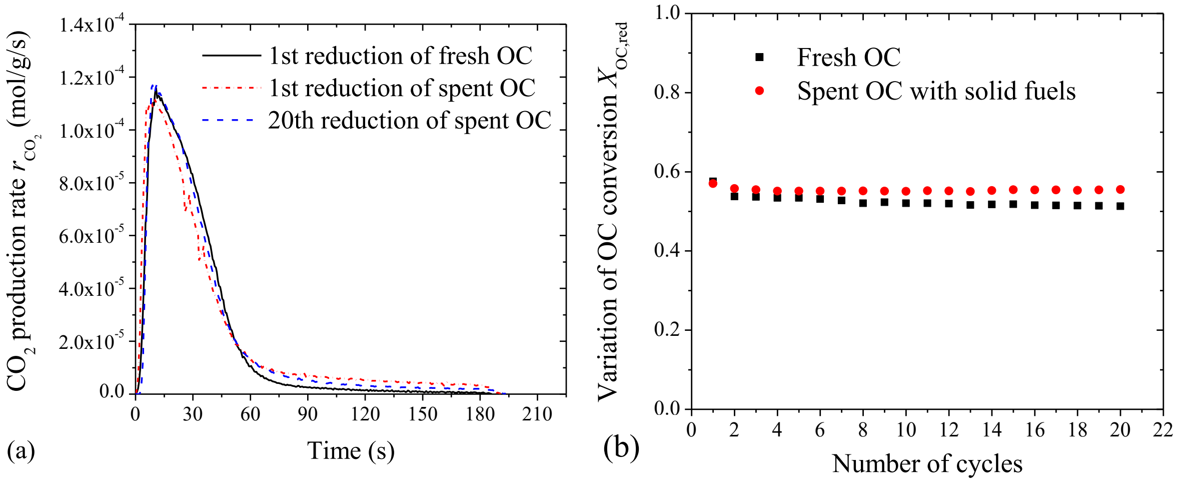

Figure S29. CLC of gaseous fuel with fresh and used oxygen carriers. (a) Rate of production of CO_2 as a function of time and (b) overall oxygen carrier conversion variation ($X_{OC, red}$) from CuO to Cu measured by the O_2 consumed to produce CO_2 during the reduction stage as a function of cycle number in CLC of CO with the fresh and spent oxygen carriers recovered in the solid fuels experiments. A batch of 0.5 g oxygen carriers were fluidized by 2.4 vol% CO in N_2 with a total flow rate of 70.6 cm^3/s at 950 °C, oxidation was carried out with air (50.9 cm^3/s).

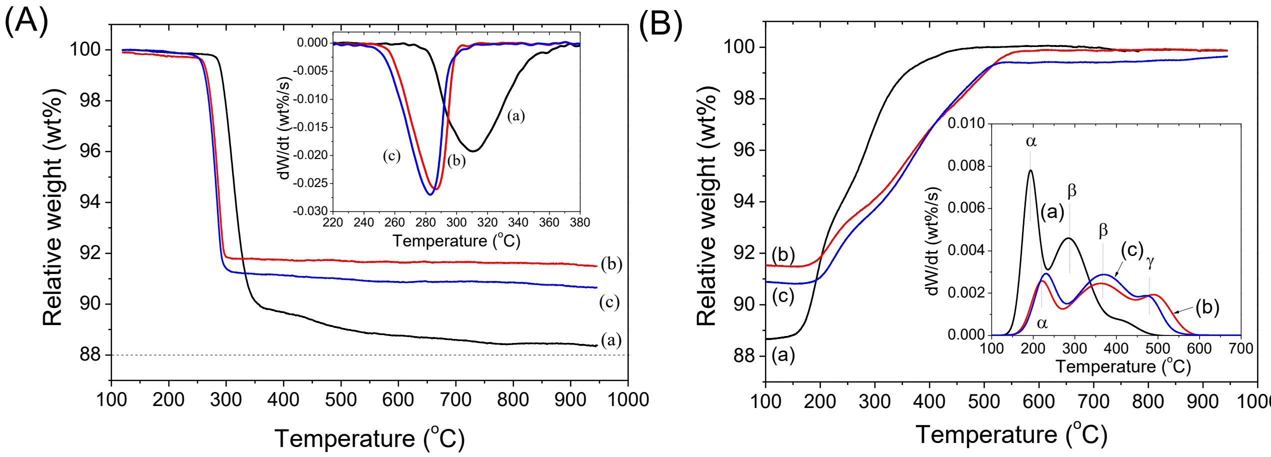

Figure S30. Temperature programmed reduction and oxidation of Cu-based oxygen carrier. (A) reduction in 5.0 vol% H_2/ N_2 (50 mL min^{-1}, STP) and (B) oxidation in air (50 mL min^{-1}, STP) for profiles for (a) fresh as-calcined oxygen carriers, (b) used samples with solid fuels over 850-985 °C in the presence of steam, and (c) used samples further exposed to 20 redox cycles at 950 °C.

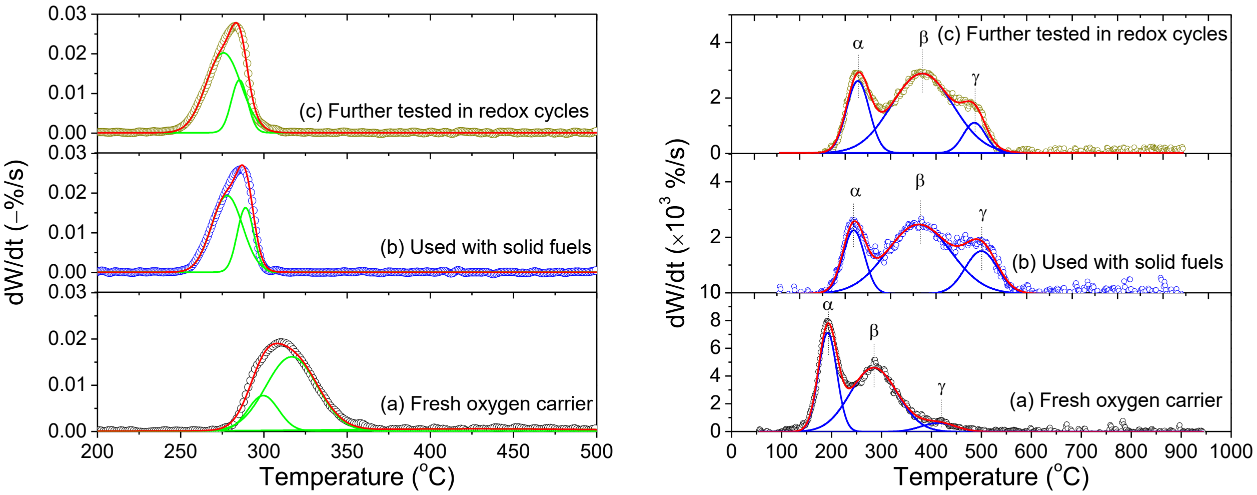

Figure S31. Fitting of TPR and TPO rate curves. Fitting of reduction (left) and oxidation (right) peaks for (a) fresh as-calcined oxygen carriers, (b) used samples with solid fuels over 850-985 °C, and (c) used samples further exposed to 20 redox cycles at 950 °C.

The weight loss rates curves were further processed and deconvoluted as shown in Fig. S31. For the fresh oxygen carriers, the reduction of highly dispersed CuO was not observed at low temperatures (100-200°C) because the high loading of CuO and calcination at a high temperature yielded crystalline CuO in the sodium-stabilized Al_2O_3 support. The fast weight loss rate was assigned to the consumption of O_2 by H_2 reduction of the dominant phase, mainly the bulk CuO. The derivative of weight loss presents a major peak at 311 °C but with a small shoulder peak on the right hand side. According to the literature, the reduction of CuO to Cu at this lower temperature (300 °C) may involve the two-step reduction: CuO→Cu_2O (around 300 °C) and Cu_2O→Cu (320 °C) with a small shoulder at 300 °C on the peak.[8] The slow reduction during the later period accompanied with a broad low-intensity peak at about 450 °C could be attributed to the reduction of $CuAl_2O_4$ with slow weight loss of about 1.4% from 400 °C until 800 °C. The further loss of weight could be due to reduction of delafossite $CuAlO_2$. Currently the species of the O_2 can not be clearly differentiated from lattice O_2 or $CuAlO_2$.

As for the used samples in solid fuels experiments and further tested in redox cycles, the major reduction peak appeared similar but significantly different from the fresh oxygen carriers. The major reduction peak could be deconvoluted into two peaks, corresponding to the two-step of reduction of CuO→Cu_2O (around 275 °C) and Cu_2O→Cu (285 °C).

Interestingly the reduction reactivity of the oxygen carriers did not deactivate and instead the weight loss rate increased from 0.019 wt%/s to 0.026 wt%/s and did not change even though the sample was further tested by 20 redox cycles.

However, the rates of oxidation during the temperature programmed oxidation of reduced oxygen carriers are quite different. Overall the rates of oxidation were much slower than that of reduction,

thus the peaks could be differentiated clearly. For the fresh oxygen carriers, there are evidently two major oxidation peaks corresponding to oxidation of Cu→Cu_2O and Cu_2O→CuO with center temperatures at 192 °C (α) and 285 °C (β), respectively. The third small peak at 414 °C (γ) is not very clear, but could be assigned to the consumption of lattice O_2, as evidenced by the used samples. For the used samples, the profiles are quite different from that of fresh oxygen carriers, with much lower rate of oxidation and complete oxidation until 550 °C. Interestingly, for the samples further exposed to redox cycles, γ emerged earlier at 485 °C.

The center temperatures calculated areas of fitting peaks of TPR and TPO are summarized in Table S4.

Table S4. Quantitative fitting of the peaks in TPR and TPO of oxygen carrier

Samples	Center temperature of reduction (°C)				Cumulative area percentage (wt%/oxygen carrier)					
	α	β	γ	η	α	β	γ	η	Total[a]	Total[b]
Fresh	300	316	447	710	2.48	7.31	1.15	0.20	11.14	11.60
Used with solid fuels	278	289	-		5.42	2.44	n.d.	n.d.	7.87	8.60
Further redox cycles	275	285	-		6.56	1.82	n.d.	n.d.	8.38	9.30

Samples	Center temperature of oxidation (°C)			Cumulative area percentage (wt%/oxygen carrier)				
	α	β	γ	α	β	γ	Total[a]	Total[c]
Fresh	192	285	414	4.10	6.57	0.64	11.31	11.40
Used with solid fuels	219	362	499	1.69	5.30	1.61	8.60	8.55
Further redox cycles	228	370	485	2.0	5.79	0.80	8.59	9.20

Note: [a] calculated by the integration of areas of fitting peaks.
[b] calculated by overall weight loss of TG curves in Fig. S28a, including the weight loss during the 1 h isothermal period at 950 °C.
[c] calculated by overall weight increase of TG curves in Fig. S28b, including the weight increase during the 1 h isothermal period at 950 °C.

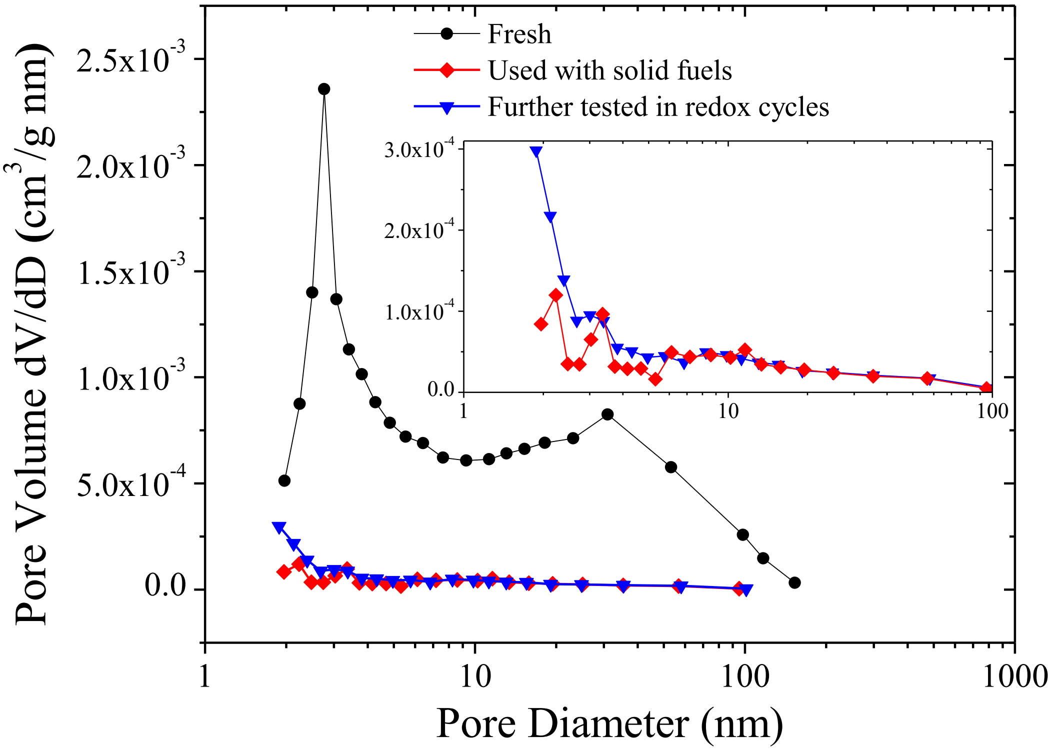

Figure S32. Pore size distribution of the fresh oxygen carriers and used samples in solid fuels experiments and samples further tested in redox cycles at 950 °C. Data derived from N_2 adsorption isotherms at 77K. For inset, ordinate has units of cm^3/g nm, abscissa is in nm.

References

1. Song, Q.; Liu, W.; Bohn, C. D.; Harper, R. N.; Sivaniah, E.; Scott, S. A.; Dennis, J. S., A high performance oxygen storage material for chemical looping processes with CO_2 capture. *Energy Environ. Sci.* **2013,** *6* (1), 288-298.
2. Wen, C. Y.; Yu, Y. H., A Generalized method for Predicting the Minimum Fluidization Velocity. *AIChE J.* **1996,** *12* (3), 610-612.
3. Xiao, R.; Song, Q.; Song, M.; Lu, Z.; Zhang, S.; Shen, L., Pressurized chemical-looping combustion of coal with an iron ore-based oxygen carrier. *Combust. Flame* **2010,** *157* (6), 1140-1153.
4. Weisz, P. B.; Prater, C. D.; W.G. Frankenburg, V. I. K.; Rideal, E. K., Interpretation of Measurements in Experimental Catalysis. In *Adv. Catal.*, Volume 6 ed.; Academic Press: 1954; pp 143-196.
5. Alejandre, A.; Medina, F.; Rodriguez, X.; Salagre, P.; Sueiras, J. E., Preparation and Activity of Cu-Al Mixed Oxides via Hydrotalcite-like Precursors for the Oxidation of Phenol Aqueous Solutions. *J. Catal.* **1999,** *188* (2), 311-324.
6. Behrens, M.; Kasatkin, I.; Kühl, S.; Weinberg, G., Phase-Pure Cu,Zn,Al Hydrotalcite-like Materials as Precursors for Copper rich $Cu/ZnO/Al_2O_3$ Catalysts. *Chem. Mater.* **2009,** *22* (2), 386-397.
7. Barin, I.; Platzki, G., *Thermochemical data of pure substances*. VCH: Weinheim; New York, 1995.
8. Bridier, B.; López, N.; Pérez-Ramírez, J., Partial hydrogenation of propyne over copper-based catalysts and comparison with nickel-based analogues. *J. Catal.* **2010,** *269* (1), 80-92.
